# Targeting Protein Tyrosine Phosphatase 1B (PTP1B) to Improve Insulin Sensitivity Using Indole-Fused Glycyrrhetinic Acid Conjugates with Amino Acids

**DOI:** 10.1021/acsmedchemlett.5c00701

**Published:** 2026-02-09

**Authors:** Ledy De-la-Cruz-Martínez, David Equihua-González, Diana Laura Torres-Chacón, Erandi Ortiz-Barragán, J. Martin Torres-Valencia, Rubria Marlen Martínez-Casares, Jaime Pérez-Villanueva, Martín González-Andrade, Julio César Almanza-Pérez, Francisco Cortés-Benítez

**Affiliations:** † Doctorado en Ciencias Farmacéuticas, División de Ciencias Biológicas y de la Salud, Universidad Autónoma Metropolitana − Unidad Xochimilco, Ciudad de México 04960, Mexico; ‡ Departamento de Sistemas Biológicos, División de Ciencias Biológicas y de la Salud, Universidad Autónoma Metropolitana − Unidad Xochimilco, Ciudad de México 04960, Mexico; § Área Académica de Química, Universidad Autónoma del Estado de Hidalgo, Hidalgo 42184, Mexico; ∥ Laboratorio de Biosensores y Modelaje Molecular, Departamento de Bioquímica, Facultad de Medicina, Universidad Nacional Autónoma de México, Ciudad de México 04510, Mexico; ⊥ Laboratorio de Farmacología, Departamento de Ciencias de la Salud, División de Ciencias Biológicas y de la Salud,Universidad Autónoma Metropolitana − Unidad Iztapalapa, Ciudad de México 09340, Mexico

**Keywords:** Protein Tyrosine Phosphatase 1B, Glycyrrhetinic acid, Amino acid conjugates, GLUT4 expression, Type 2 diabetes

## Abstract

Protein Tyrosine Phosphatase 1B (PTP1B) is a crucial enzyme that significantly modulates insulin and leptin signaling, making it a highly promising target for the treatment of type 2 diabetes (T2D). We previously reported the synthesis and inhibitory activity of FC-114, an indole-fused glycyrrhetinic acid derivative that potently inhibits PTP1B. In this study, we synthesized four FC-114 conjugates with amino acids at the C30 position to enhance their inhibitory activity against PTP1B *in vitro*. The results indicated that incorporating glycine (compound **5a**) and serine (compound **5d**) substantially enhanced inhibitory activity against PTP1B, achieving up to 4-fold greater potency, with submicromolar IC_50_ values of 0.64 and 0.54 μM, respectively. Inhibitory assessments of the short form (*h*PTP1B_1‑285_) and long form (*h*PTP1B_1‑400_) of PTP1B, along with enzymatic kinetics studies, molecular docking, and molecular dynamics analyses, suggested a mechanism consistent with uncompetitive inhibition, potentially involving a binding to the disordered C-terminal domain. Furthermore, both FC-114 conjugates with glycine (**5a**) and arginine (**5b**) significantly enhanced the mRNA expression of the GLUT4 receptor in C2C12 myoblast cells. Additionally, these compounds reduced glucose levels during the insulin tolerance test in streptozotocin-induced diabetic mice.

## Graphical abstract

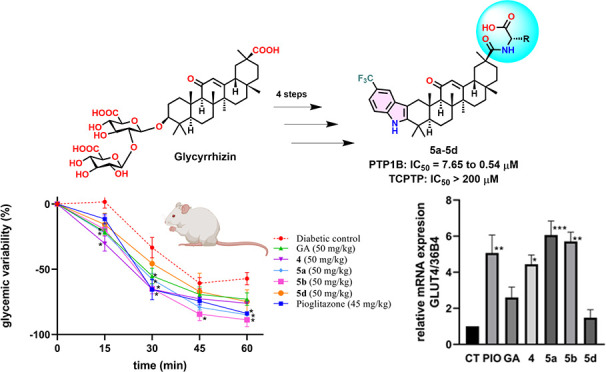

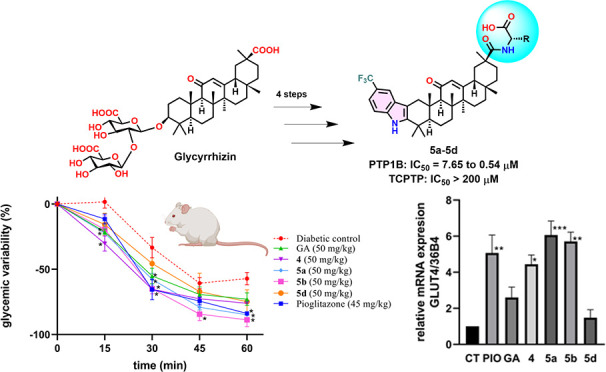

Diabetes represents one of the fastest-growing global health challenges, as evidenced by 2024 estimates showing 635 million individuals with impaired glucose tolerance and 488 million with impaired fasting glucose, along with over 3.4 million adults (aged 20–79) who died from related complications.^1^ The disorder is a chronic metabolic condition characterized by elevated blood sugar levels due to insufficient insulin production, insulin deficiency, or decreased insulin sensitivity. Type 2 diabetes (T2D), which accounts for over 90% of all global cases, is the primary concern and is projected to become the second leading cause of global disease burden by 2050.^1,2^ Its characteristic pathology begins with insulin resistance—the reduced responsiveness of peripheral tissues to insulin—which drives compensatory hyperinsulinemia. This excessive demand eventually stresses the system, progressing to irreversible pancreatic β-cell dysfunction and subsequent impaired insulin secretion.^1,3^ The treatments for T2D include sulphonylureas, α-glucosidase inhibitors, thiazolidinediones, dipeptidyl peptidase-4 (DPP-4) inhibitors, glucagon-like peptide-1 receptor (GLP-1R) and gastric inhibitory peptide (GIP) agonists, as well as sodium-glucose cotransporter 2 (SGLT2) inhibitors. However, adverse effects, suboptimal efficacy, or lack of selectivity limit their use.^4,5^

In this context, Protein Tyrosine Phosphatase 1B (PTP1B) is a well-recognized target of T2D, as its inhibition enhances insulin sensitivity and improves glucose homeostasis. Unfortunately, many PTP1B inhibitors lack selectivity and also inhibit closely related phosphatases, such as T-cell Protein Tyrosine Phosphatase (TCPTP), leading to undesirable side effects.^6–11^ Therefore, to date, no PTP1B inhibitors have progressed beyond Phase II clinical trials.^12,13^ We previously reported compound FC-114 (**4**), an indole-fused derivative of glycyrrhetinic acid, which demonstrated potent PTP1B inhibition *in vitro* and improved glucose metabolism in streptozotocin (STZ)-induced diabetic rats.^11,14,15^ However, the *in vivo* effect was less significant than expected, likely due to the compound’s high lipophilicity, which may have limited its aqueous solubility and overall bioavailability. To mitigate these limitations, we synthesized derivatives of compound **4** that are conjugated to amino acids at position C30, including Gly (**5a**), Arg (**5b**), Thr (**5c**), and Ser (**5d**). The strategy of amino acid conjugation is frequently employed in medicinal chemistry due to its numerous advantages: it enhances structural diversity to improve interactions with targets, increases aqueous solubility, and reduces toxicity.^16,17^ The compounds **5a**, **5b**, **5c**, and **5d** were synthesized following the procedure outlined in Scheme 1. Glycyrrhizin was used as a raw material and hydrolyzed to produce Glycyrrhetinic acid (GA, **2**). The secondary alcohol at the C3 position of GA was then oxidized to yield 3-oxoglycyrrhetinic acid (**3**). Subsequently, the Fischer indole synthesis was employed to react this intermediate with 4-(trifluoromethyl)­phenylhydrazine, yielding compound **4**. For the synthesis of amino acid conjugates, compound **4** was reacted with the methyl esters of l-amino acids Gly, Arg, Thr, and Ser using 1,1'-carbonyldiimidazole (CDI) as a coupling agent, followed by alkaline hydrolysis, affording compounds **5a**–**5d** in moderate yields.

**Scheme 1:**
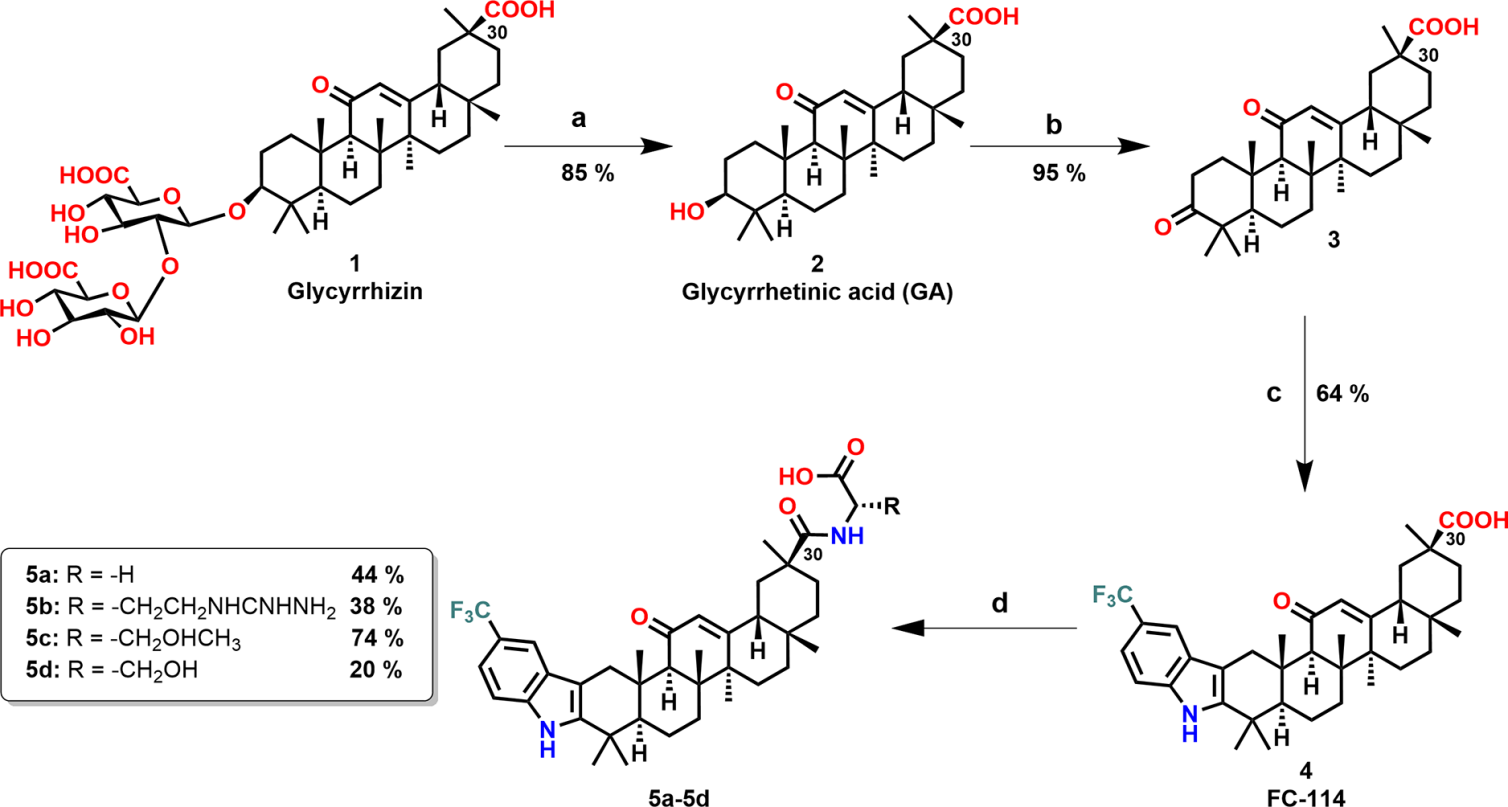
^a^

a Reagents and conditions for the synthesis of compounds **5a**–**5d** from glycyrrhizin: (a) HCl 1 M, reflux 18 h; (b) CrO_3_/H_2_SO_4_, THF, 0 °C to r.t., 2 h; (c) 4-(trifluoromethyl)­phenylhydrazine hydrochloride, AcOH, reflux; (d) 1. CDI, l-amino acid methyl ester, DCM or DMF, rt or 120 °C; 2. KOH/H_2_O, MeOH.

The structures of the final compounds were confirmed by ^1^H and ^13^C NMR, along with COSY, HMBC, and HSQC experiments (Supporting Information Figures S1–S20). The chemical shift data of the indole ring as well as the triterpenoid skeleton of these compounds are in accordance with the data of compound **4**.^11,14,15^ However, in the ^1^H NMR spectra, we found some representative signals in the aromatic region from 7.22 to 7.91 ppm characteristic of NH signal of amide group (**5a** (7.91 ppm, t, *J* = 5.8 Hz), **5b** (7.70, d, *J* = 7.6 Hz), **5c** (6.99 ppm, d, *J* = 6.1 Hz) and **5d** (7.22, d, *J* = 6.1 Hz)). On the other hand, doublets of doublets signals with *J* = 17.8, 5.3, and 17.3, 5.5 Hz at 3.80 and 3.67 ppm, respectively, were assigned to Hα from glycine in the **5a** compound. For the **5b** compound, there are two broad singlets at 7.81 and 7.00 ppm assigned to NH from the guanidine group. Additionally, a multiplet at 4.23 ppm is assigned to Cα, and a signal at 156.88 ppm was assigned to Cε of arginine. Furthermore, multiple and singlet signals at 3.95 (1H) and 1.78 (3H) ppm, respectively, corresponding to Cα and Cγ from threonine in the compound **5c**. Finally, a broad singlet (4.56 ppm) and a multiple signal (4.06–3.96 ppm) were assigned to OH and Cα from serine in derivative **5d**.

The compounds **5a**–**5d**, and positive controls GA, Ursolic acid (UA), Ertiprotafib, and Sodium Orthovanadate (SOV) were assessed for their inhibitory effect on the long form of PTP1B enzyme (*h*PTP1B_1‑400_), to prevent the formation of *p*-nitrophenol from *p*-nitrophenylphosphate (pNPP). Several concentrations of new derivatives and controls were assessed, giving IC_50_ values ranging from 7.65 to 0.54 μM (Table 1). The potency order of the indole derivatives of GA is as follows: **5d** > **5a** > **5c** > **5b**. Notably, compounds **5a** (IC_50_ = 0.64 μM) and **5d** (IC_50_ = 0.54 μM) are 4-fold more potent than compound **4** (IC_50_ = 2.28 μM) and are also significantly more potent than GA (IC_50_ = 56.49 μM), being 105-fold and 88-fold more potent, respectively. Additionally, both compounds are 12-fold and 10-fold more potent than UA, a pentacyclic triterpene widely reported as a PTP1B inhibitor. Moreover, they demonstrated 3-fold greater potency than the gold-standard PTP1B inhibitors Ertiprotafib (IC_50_ = 1.73 μM) and SOV (IC_50_ = 1.87 μM). Ertiprotafib is a PTP1B inhibitor that reached phase II clinical trials for the treatment of T2D^18,19^ while SOV is a potent, nonselective, and commonly used competitive inhibitor of PTP1B.^20^ In contrast, compounds **5b** (IC_50_ = 7.65 μM) and **5c** (IC_50_ = 3.42 μM) exhibited 3.3-fold and 1.5-fold lower potency than **4**, respectively.

**Table 1:** Inhibitory Activity of PTP1B and TCPTP by GA Derivatives and Positive Controls^a^

Compound	*h*PTP1B_1‑400_ IC_50_ (μM ± S.D)	*h*PTP1B_1‑285_ IC_50_ (μM ± S.D)	*h*TCPTP_1‑415_ IC_50_ (μM ± S.D)
**4**	2.28 ± 0.13	13.88 ± 0.34 ^14^	>100 ^14^
**5a**	0.64 ± 0.08	3.93 ± 0.61	>200
**5b**	7.65 ± 0.68	2.18 ± 0.18	>200
**5c**	3.42 ± 0.12	ND	ND
**5d**	0.54 ± 0.05	2.91 ± 0.34	>200
GA	56.49 ± 3.32	ND	ND
UA	6.33 ± 0.22	ND	ND
Ertiprotafib	1.73 ± 0.23	ND	>100 ^24^
SOV	1.87 ± 0.09	ND	0.52 ± 0.053 ^14^

a ND: not determined.

Several interesting observations emerged from the results regarding structure–activity relationships. For example, the addition of an amino acid at position C30 affects the inhibitory activity against PTP1B. Specifically, when glycine or serine is added, inhibitory activity increases, whereas the addition of threonine or arginine decreases activity. This indicates that smaller amino acid side chains are more favorable for enhancing inhibitory activity. Furthermore, arginine contains an ionizable guanidino group at physiological pH, which may contribute to its reduced inhibitory activity against PTP1B. In contrast, serine and threonine are polar amino acids containing primary and secondary alcohols, respectively. However, threonine also carries a methyl group that introduces steric hindrance, which likely contributes to its reduced inhibitory activity against PTP1B compared to serine.^21^

To indirectly explore the role of the disordered C-terminal region of PTP1B in the binding of synthesized compounds, we evaluated compounds **5a**, **5b**, and **5d** against the short form of PTP1B (*h*PTP1B_1–285_), which lacks this region (Supporting Information, Figure S29). This intrinsically disordered C-terminal domain of PTP1B (residues 301–400) is specific to PTP1B.^22^ In contrast, the catalytic domain of PTP1B shares a high degree of homology with other protein tyrosine phosphatases (PTPs), such as TCPTP. The results indicated that compounds **4**, **5a**, and **5d** displayed a 6- to 5-fold greater potency against *h*PTP1B_1‑400_ compared to against *h*PTP1B_1‑285_. This suggests that the disordered C-terminus of PTP1B is essential for the inhibitory activity, indicating that the primary binding region for these compounds is likely located at this site. This observation aligns with previous research, including studies on Trodusquemine (MSI-1436), an aminosterol known for its ability to inhibit PTP1B by binding to the C-terminal site.^23^ In contrast, compound **5b** was found to be 3.5-fold more potent against *h*PTP1B_1‑285_ than against *h*PTP1B_1‑400_. The presence of Arg at the C30 position of FC-114 suggests a reduced affinity of the compound for the longer form of PTP1B. The guanidine group of Arg carries a positive charge at physiological pH and can interact with negatively charged regions, particularly those around Glu or Asp residues, within the catalytic domain of PTP1B.

The catalytic domain of PTP1B shares 74% sequence homology with that of TCPTP, which plays a crucial role in immune function.^22^ To evaluate the selectivity of compounds **5a**–**5d**, we tested them against *h*TCPTP_1‑415_. As shown in Figure S30 of the Supporting Information, the results indicated that none of the synthesized compounds displayed any inhibitory activity against TCPTP, even at concentrations of up to 200 μM. Consequently, these compounds are at least 26- to 370-fold selective for PTP1B over TCPTP.

To assess their mode of inhibition on *h*PTP1B_1–400_, compounds **5a**, **5b**, and **5d** were selected for enzyme kinetic assays (Table 2). The Lineweaver–Burk plots are illustrated in Figure S31 of the Supporting Information. The PTP1B inhibitors **5a**, **5b**, and **5d** exhibited *K*_i_ values of 1.15, 0.79, and 1.91 μM, respectively. Furthermore, these compounds demonstrated uncompetitive inhibition, suggesting that they may bind to an allosteric site on PTP1B.

**Table 2:** Kinetic Parameters of *h*PTP1B_1‑400_ with Different Inhibitors

Compound	*K*_i_ (μM ± S.D)	*V*_max_ (mM/min ± S.D)	*K*_m_ (mM ± S.D)	Inhibition type
**5a**	1.15 ± 0.08	0.37 ± 0.02	0.22 ± 0.03	uncompetitive
**5b**	0.79 ± 0.12	1.11 ± 0.15	0.63 ± 0.12	uncompetitive
**5d**	1.91 ± 0.25	0.74 ± 0.07	0.41 ± 0.06	uncompetitive

Based on the inhibition types observed for compounds **5a**–**5d**, we explored their potential binding modes using a three-dimensional model of the PTP1B_1‑400_ enzyme complexed with the pNPP substrate (PTP1B_1‑400_-pNPP) previously reported by our group.^14,24^ Given that the C-terminal region of PTP1B is intrinsically disordered and that the full-length structure is based on an AlphaFold prediction, the following docking analyses are intended to provide qualitative, hypothesis-generating insights rather than definitive structural conclusions. Molecular docking simulations were conducted using three software programs: AutoDock 4.2 (The Scripps Research Institute, La Jolla, CA, USA), Vina (The Scripps Research Institute, La Jolla, CA, USA), and GOLD (The Cambridge Crystallographic Data Centre, Cambridge, UK).

To identify the preferred binding sites for the synthesized compounds within the PTP1B_1–400_-pNPP complex, a blind docking simulation was conducted using AutoDock. The resulting docking poses suggested that the compounds may preferentially sample two main regions within the enzyme, with a higher frequency of poses located in the disordered C-terminal region rather than in the catalytic domain (Figure 1A). The first site (Site 1) is a pocket that includes a portion of the catalytic domain and a significant portion of the unstructured C-terminal region, featuring the residues Lys^128^, Ile^134^, Glu^147^, Ile^149^, Pro^303^, Glu^304^, Ile^306^, Pro^307^, Arg^380^, Ala^382^, Gln^383^, Ala^384^, Pro^387^, Pro^395^, Lys^397^, and Asp^400^. The second site (Site 2) is situated in the unstructured C-terminal region, consisting of the residues: His^320^, Glu^337^, Asp^341^, Cys^344^, Pro^345^, Lys^350^, Ser^352^, Pro^353^, Ala^356^, Pro^358^, and Arg^371^.

**Figure 1:**
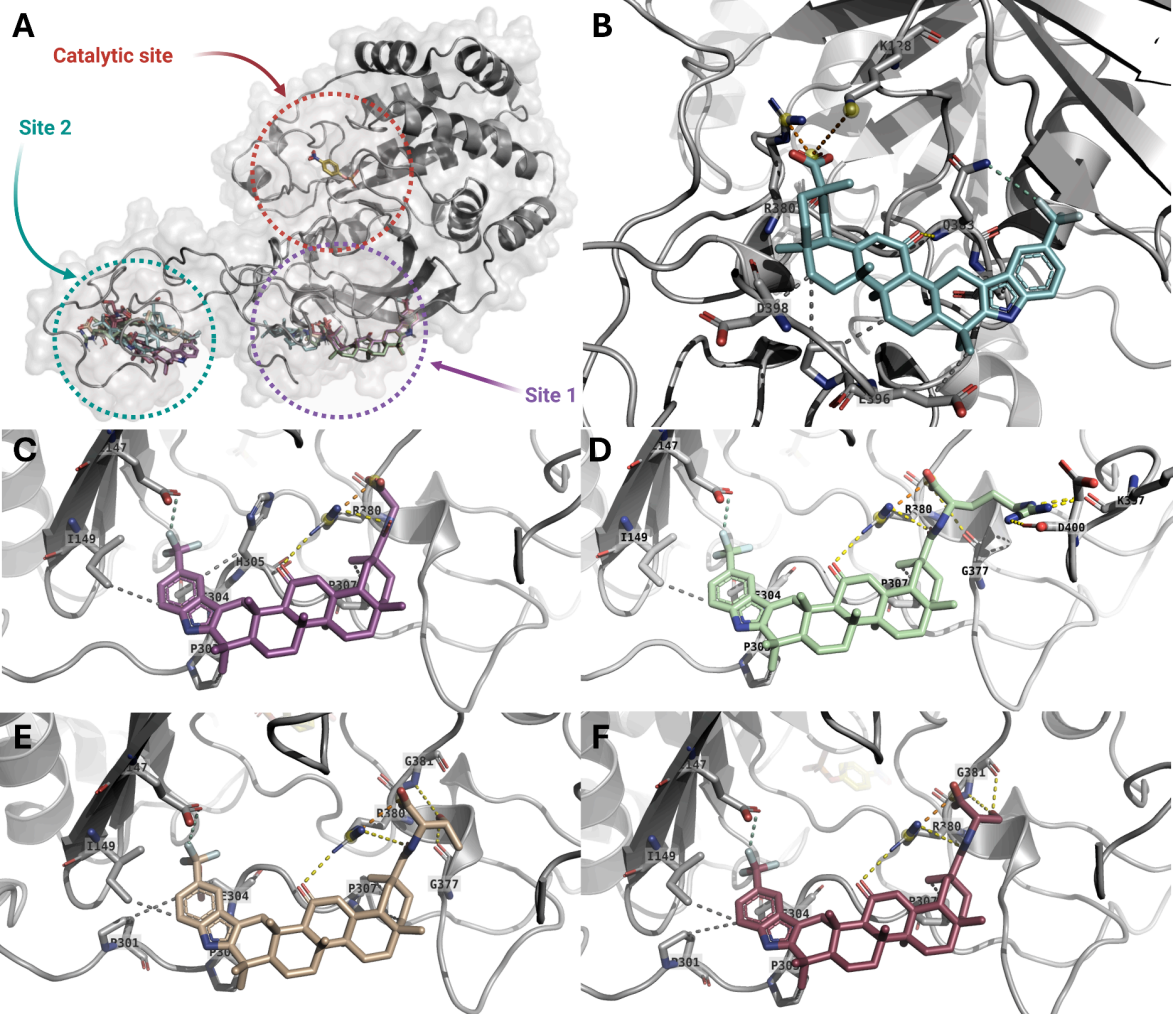
(A) Predicted binding modes of **4** (cyan) and its amino acid conjugates **5a** (violet), **5b** (green), **5c** (wheat), and **5d** (raspberry), after blind docking against PTP1B_1‑400_-pNPP complex (gray). The pNPP substrate is highlighted in yellow. (B–F) Predicted binding mode of compounds **4** and **5a**–**5d** within site 1 of the PTP1B_1‑400_-pNPP complex. Dotted yellow lines indicate hydrogen bond interactions, while orange, gray, and cyan dotted lines represent salt bridge interactions, hydrophobic contacts, and halogen bonds, respectively.

Subsequently, site-directed docking simulations of the GA derivatives were performed at Sites 1 and 2 using AutoDock, Vina, and GOLD (Supporting Information Table S1). The docking analyses indicated that the newly synthesized compounds generally achieved more favorable docking scores at Site 1 (particularly with AutoDock and ChemPLP) compared to compound **4**. Across all three docking programs, the top-ranked poses of compounds **5a**–**5d** at Site 1 adopted broadly similar orientations, with the main differences arising from the orientation of the amino acid side chains attached at the C30 position (Figure 1C-1F and Supporting Information, Figures S32A–S32E). In contrast, compound **4** adopted a distinct docking pose within this region (Figure 1B).

Inspection of the top-ranked docking poses suggested a network of potential hydrophobic, hydrogen-bonding, and ionic interactions that may contribute to the observed binding trends. For compounds **5a**–**5d**, the triterpenoid scaffold and indole moiety were positioned in proximity to Pro^303^, Pro^307^, and Ile^149^, consistent with hydrophobic contacts. In addition, the trifluoromethyl group was oriented toward Glu^147^ and Glu^304^, allowing for potential halogen interactions, while the carbonyl groups at C11 and C30 were positioned to act as hydrogen bond acceptors for Arg^380^. In contrast, compound **4** was oriented such that its triterpenoid scaffold performed hydrophobic interactions with Ile^134^, Ala^383^, Pro^384^, Pro^387^, and Pro^395^, while its trifluoromethyl and C11 carbonyl groups were positioned near Gln^393^ to form H-bonds. The incorporation of amino acids at C30 in compounds **5a**–**5d** increased the number of potential hydrogen-bonding or ionic interactions; for example, the amide group in compound **5a** form an H-bond with Gly^377^, the hydroxyl groups in compounds **5c** and **5d** acted as H-bond donors with Gly^377^ and Gly^381^, respectively, and the guanidinium group in compound **5b** was oriented toward Asp^400^ and Lys^397^ forming salt bridge interactions.

At Site 2, the docking analyses suggested that the amino acid-conjugated derivatives generally achieved more favorable docking scores with AutoDock and GOLD (ChemPLP), with the exception of compound **5c**, compared to compound **4**. The top-ranked poses of compounds **5a**–**5d** adopted orientations opposite to that of compound **4** within this region (Supporting Information, Figures S32F–S32J). As observed for Site 1, amino acid conjugation increased the number of potential hydrogen-bonding and ionic interactions. For instance, the carboxylate group of compound **4** was positioned near His^320^ and Asn^321^, whereas in compound **5a**, the newly introduced amide group forms H-bonds with Asn^321^ and Pro^358^. In compounds **5c** and **5d**, the hydroxyl groups were positioned to form H-bonds with Glu^336^, Glu^337^, and Asp^341^, as well as Gln^332^, Val^334^, and Glu^336^, respectively, while the guanidinium group of compound **5b** was oriented toward Glu^336^ and Asp^341^ to form salt-bridge interactions as well as an H-Bond with Ser^352^.

To gain a better understanding of the stability of the PTP1B_1–400_-pNPP-ligand complexes resulting from molecular docking simulations, we conducted molecular dynamics simulations using YASARA Structure^25,26^ program and AMBER 11 force field (Figures 2A–2G and Supporting Information Table S2). Overall, the trajectory of 300 ns simulations reveals a clear site-dependent modulation of protein dynamics and ligand accommodation, highlighting differences that are not fully captured by binding free-energy estimates. At Site 2, ligand binding induced variable degrees of global conformational deviation. While **5b** displayed the lowest average Cα RMSD, suggesting limited structural perturbation, this ligand was associated with comparatively weak binding energetics. In contrast, **5a**, **5c**, and **5d** induced larger conformational adjustments, reflected by higher RMSD values, consistent with a binding mode that requires protein reorganization. Among these ligands, **5c** exhibited the most favorable MM/PBSA binding free energy; however, this energetic advantage was accompanied by increased structural deviation and did not translate into a clear stabilization of protein dynamics, as indicated by RMSF analysis. These observations suggest that strong binding energetics at Site 2 may arise from transient or highly adaptive interactions rather than from a consistently stable binding mode.

**Figure 2:**
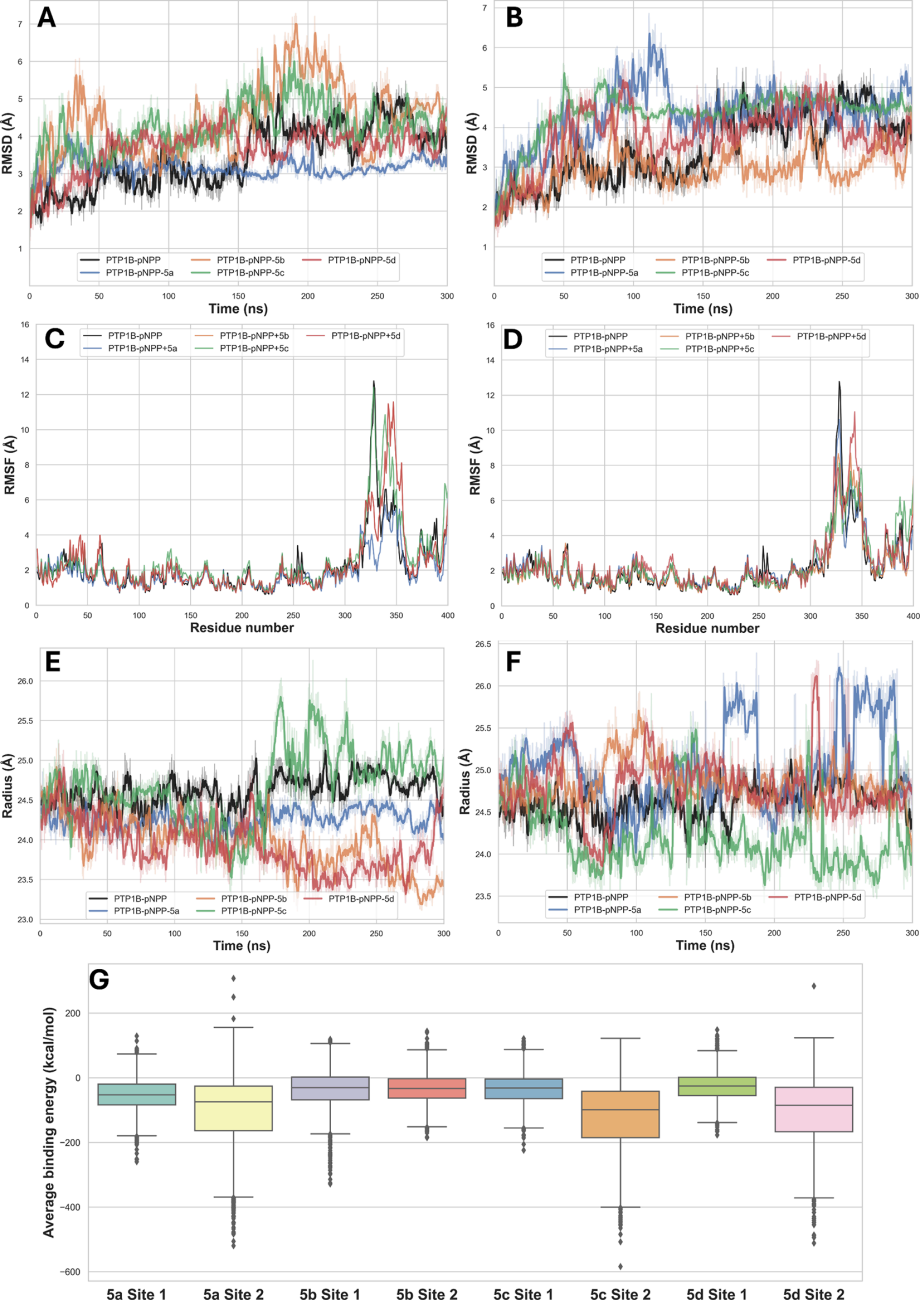
Standard RMSD of PTP1B_1‑400_-pNPP complex, as well as PTP1B_1‑400_-pNPP-ligand systems from docking complexes at site 1 (A) and site 2 (B). RMSF of PTP1B_1‑400_-pNPP complex, as well as PTP1B_1‑400_-pNPP-ligand systems from docking complexes at site 1 (C) and site 2 (D). Radius of gyration (*R*_g_) of PTP1B_1‑400_-pNPP complex, as well as PTP1B_1–400_-pNPP-ligand systems from docking complexes at site 1 (E) and site 2 (F). Boxplot of average binding energy of ligands using the MM/PBSA method as a function of 300 ns of MD simulation within the PTP1B_1‑400_-pNPP complex (G). The central line within the box represents the median of the binding energy. The lower and upper edges of the box correspond to the first (Q1) and third quartiles (Q3), respectively, defining the interquartile range (IQR). The whiskers extend to the minimum and maximum values that do not exceed 1.5 times the IQR. Points beyond the whiskers represent outliers.

In contrast, Site 1 displayed a distinct dynamic profile characterized by improved structural stabilization upon ligand binding. Notably, complexes formed with **5a** and **5d** displayed radius of gyration values comparable to that of the PTP1B_1–400_-pNPP protein, indicating preservation of the overall protein compactness upon ligand binding and suggesting minimal disruption of the global fold. Moreover, **5a** showed the lowest RMSD and the lowest RMSF among the evaluated ligands at this site, indicating a stabilizing effect on both global and local protein motions. This behavior was accompanied by the most favorable binding free energy at Site 1, suggesting a binding mode that is energetically favorable while minimizing conformational strain on the protein. **5d**, although associated with less favorable MM/PBSA energies, exhibited a dynamic behavior comparable to that of **5a**, maintaining moderate RMSD values and avoiding excessive local flexibility. This combination suggests a binding mode that is dynamically well tolerated by the protein, potentially enabling effective inhibition through sustained interactions rather than maximal static affinity.

Comparative analysis of the two sites indicates that ligands that favor binding at Site 1 tend to reduce protein flexibility and promote more coherent dynamic behavior, features commonly associated with functionally relevant inhibition of PTP1B. In this context, compounds **5a** and **5d** emerge as the most balanced ligands, combining acceptable binding energetics with favorable dynamic stabilization of the enzyme. Conversely, ligands primarily optimized for Site 1 binding appear to rely on stronger energetic contributions that are not necessarily accompanied by optimal dynamic stabilization.

Taken together, these results suggest that dynamic stabilization of PTP1B, rather than maximal binding free energy alone, is a key determinant of effective ligand behavior, and support Site 1 as a relevant target region for further optimization of this compound series. However, orthogonal experimental validation (e.g., mutagenesis, biophysical binding assays, or competition studies) will be required in future work to confirm the proposed interaction site.

To assess whether amino acid conjugates exhibit favorable drug-like properties, including pharmacokinetic properties, they were analyzed using the SwissADME server.^27^ In Table S3 and Figures S33−S37 of the Supporting Information, the physicochemical properties are predicted (lipophilicity, size, polarity, solubility, flexibility, and saturation). Solubility is indicated by Log *S*, where insoluble (<−10), poorly (<−6), moderately (<−4), soluble (<−2), and highly (<0).

The predicted aqueous solubility shows a favorable trend when Gly (−9.21) or Arg (−9.22) is added at C30 of compound **4** (−9.48). On the other hand, unsaturation is determined by the fraction of carbons in the *sp^3^* hybridization, which should be no less than 0.25. At the same time, that flexibility is assessed by the number of rotatable bonds, which should not exceed 9.^28^ Compound **4** and derivatives **5a**–**5d** demonstrated *sp* ^*3*^ fractions greater than 0.25. Notably, **5a** (0.67) and **5b** (0.65) showed lower values than compounds **4** (0.68) and **5c** (0.68), respectively. Furthermore, TPSA is the sum of the contributions to the molecular (usually van der Waals) surface area of polar atoms such as oxygen, nitrogen, and their attached hydrogens; the values of TPSA in Å^2^ within the range 140 > TPSA > 60 are indications of excellent intestinal absorption and good blood-brain barrier penetration. All compounds demonstrated TPSA values >60 and <140 Å^2^. Additionally, the pharmacokinetic analysis predicted that compound **5b**, but not compounds **4**, **5a**, **5c**, and **5d**, are substrates of the permeability glycoprotein (P-gp), an important protein in cell membranes that actively pumps many extraneous substances out of cells.^29^ Despite the low absolute solubility values, the structural modifications significantly improved the polar surface area (TPSA). However, further experimental solubility assays and pharmacokinetic (PK) studies are necessary to validate these *in silico* predictions and confirm the impact of these structural modifications on the compounds’ bioavailability.

Based on these predictions and their favorable *in vitro* profiles, compounds **5a**, **5b**, and **5d** were selected to assess their effects on GLUT4 expression and to conduct *in vivo* evaluations to further investigate their potential antihyperglycemic and insulin-sensitizing effects.

To assess whether inhibiting PTP1B with the new compounds enhances GLUT4 expression through increased insulin sensitivity, we evaluated GLUT4 levels in C2C12 myoblasts in the presence or absence of insulin (Figure 3). Pioglitazone, an insulin sensitizer and PPAR-γ agonist that enhances GLUT4 expression,^30–32^ was used as a positive control. In the absence of insulin, only Pioglitazone induced a significant GLUT4 expression. Interestingly, in the presence of insulin (0.8 μM), compounds **4**, **5a**, **5b**, and **5d** at 1 μM significantly increased GLUT4 expression, suggesting their potential to enhance insulin-stimulated glucose uptake. Moreover, despite being tested at concentrations five times lower, GLUT4 expression levels were comparable to those observed with pioglitazone.

**Figure 3:**
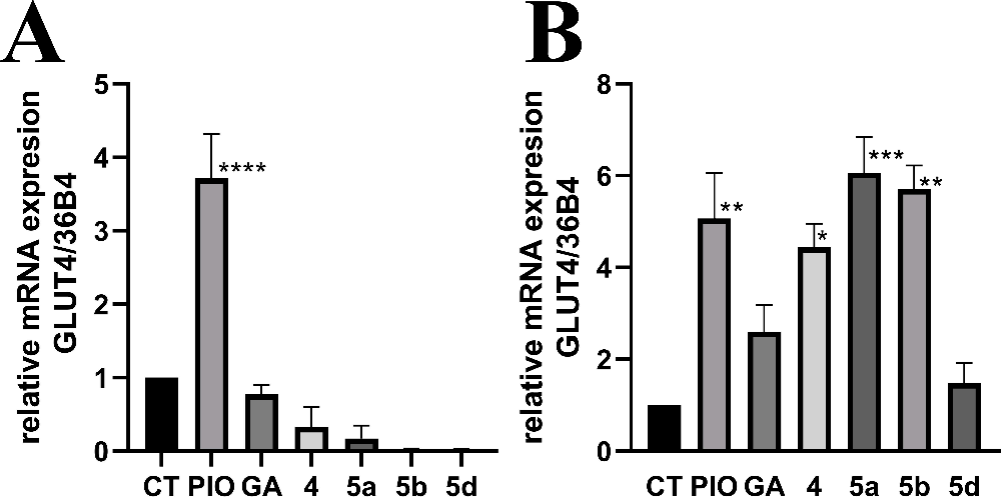
Effects of compounds **5a**, **5b**, and **5d** on GLUT4 expression in C2C12 myoblasts. (A) Relative mRNA expression levels of GLUT4 under treatments without insulin. (B) Relative mRNA expression levels of GLUT4 under treatments with insulin (0.8 μM). CT = control group; Pioglitazone = PIO (5 μM). Compounds **5a**, **5b**, and **5d** were assessed at 1 μM. Data are presented as mean ± SEM (*n* = 3). Significant difference vs control *p* ≤ 0.05­(*), *p* ≤ 0.01 (**), *p* ≤ 0.001 (***), and *p* ≤ 0.0001 (****).

Lastly, to evaluate the antihyperglycemic effect of compounds **4**, **5a**, **5b**, and **5d**, an oral glucose tolerance test (OGTT) was performed in nondiabetic mice (Figure 4A). CD-1 mice with STZ-induced diabetes are known to exhibit insulin resistance;^33^ therefore, an insulin tolerance test (ITT) was conducted in this model to investigate the insulin-sensitizing effects of compounds **4**, **5a**, **5b**, and **5d** (Figure 4B). Pioglitazone was used as a positive control. The results of this study indicated that both pioglitazone and compound **5a** significantly reduced the maximum blood glucose concentration in the OGTT compared to the nondiabetic control group. Although the effect of **5a** was less pronounced than that of pioglitazone, the observed reduction indicates a moderate improvement in glucose tolerance, suggesting partial activation of insulin-sensitizing pathways.

**Figure 4:**
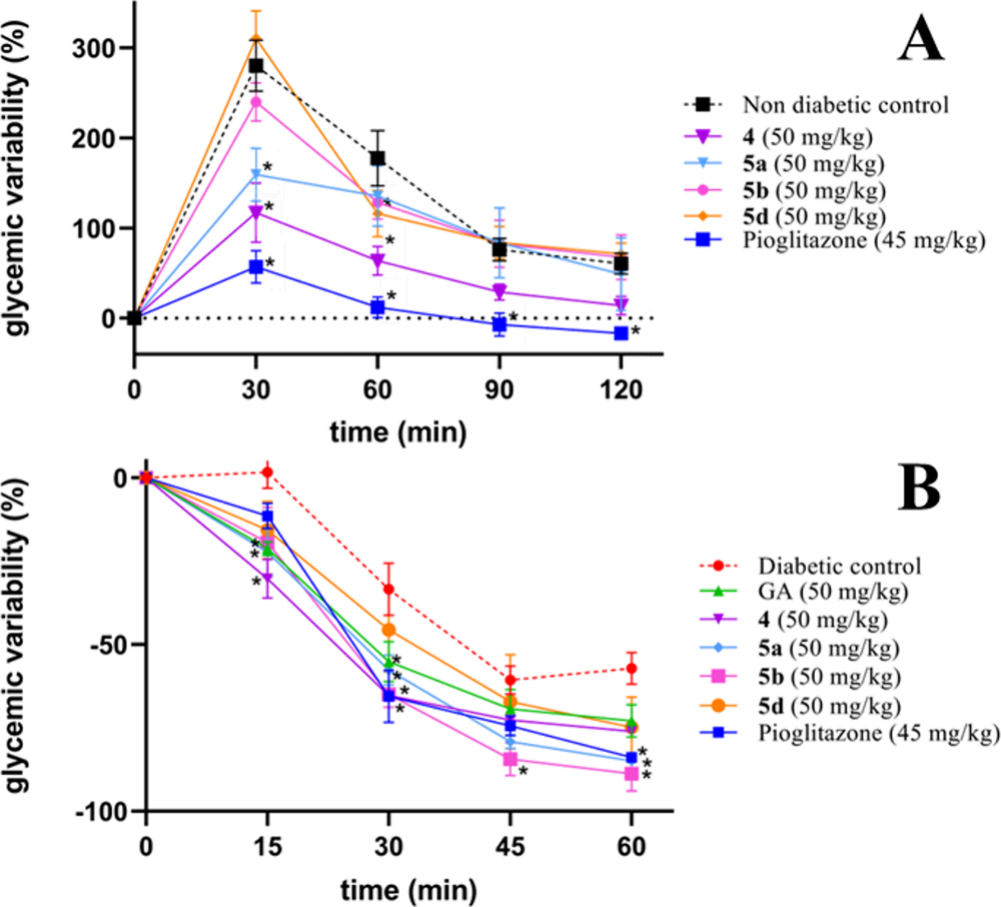
(A) Glycemic variability induced by compound **4** and its derivatives (**5a**, **5b**, and **5d**) at 50 mg/kg during oral glucose tolerance tests (OGTTs) in normal male CD1 mice. Treatments were administered 30 min before glucose administration. (B) Insulin tolerance tests (ITTs) in STZ-diabetic mice were performed after intraperitoneal insulin administration (0.75 U/kg). Pioglitazone (45 mg/kg) and GA (50 mg/kg) were used as positive controls. Data are presented as mean ± SEM (*n* = 4). *Significant difference vs the diabetic control group (*p* ≤ 0.05).

In contrast, the ITT in STZ-diabetic mice showed that compound **4** (50 mg/kg) and its amino acid conjugate **5a** (50 mg/kg) markedly decreased glucose levels, suggesting a positive impact on insulin sensitivity. Notably, compound **5b** (50 mg/kg) reduced blood glucose levels comparable to those of pioglitazone (45 mg/kg) in this acute assay, highlighting its insulin-sensitizing potential. However, further investigations involving other *in vivo* insulin-resistant models are necessary.

Collectively, these findings provide a preliminary proof-of-concept that compound **5b**, while exhibiting moderate antihyperglycemic activity under nondiabetic conditions, can exert a significant insulin-sensitizing effect in a diabetic model. The observed activity supports its potential as a promising lead candidate for further investigation as an insulin-sensitizing agent.

## Supplementary Material



## Notes

Synthesis of compounds **5a**–**5d**, including ^1^H and ^13^C NMR spectra, HPLC, and ESI-MS data for these compounds, binding modes obtained from molecular docking simulations, molecular dynamics data, and cell viability results for C2C12 cells, along with additional figures and tables as mentioned in the text (PDF)

## Abbreviations

GLUT4: glucose transporter type 4
ITT: insulin tolerance test
pNPP: *p*-nitrophenylphosphate
PI3K: phosphatidylinositol 3-kinase
PPAR−γ: peroxisome proliferator-activated receptor gamma
PTP1B: protein tyrosine phosphatase 1B
TCPTP: T-cell protein tyrosine phosphatase
SGLT2: sodium-glucose cotransporter 2
T2D: type 2 diabetes
