# Supplementary material for: Targeting Protein Tyrosine Phosphatase 1B (PTP1B) to Improve Insulin Sensitivity Using Indole-Fused Glycyrrhetinic Acid Conjugates with Amino Acids
Source: ACS Med Chem Lett. 2026 Feb 9;17(3):679–87. doi: 10.1021/acsmedchemlett.5c00701 (PMC12989875; doi:10.1021/acsmedchemlett.5c00701)
Supplement: Supplementary file 1 [file ml5c00701_si_001.pdf]

Supporting Information

Targeting Protein Tyrosine Phosphatase 1B (PTP1B) to Improve Insulin Sensitivity Using Indole-Fused Glycyrrhetinic Acid Conjugates with Amino Acids

Ledy De-la-Cruz-Martínez,[a,b,d,e] *David Equihua-González,*[b] *Diana-Laura Torres-Chacón,*[e] *Erandi Ortiz-Barragán,*[e] *J. Martin Torres-Valencia,*[c] *Rubria Marlen Martínez-Casares,*[b] *Jaime Pérez-Villanueva,*[b] *Martín González-Andrade,*[*d] *Julio César Almanza-Pérez,*[*e] *Francisco Cortés-Benítez*[*b]

[a]Doctorado en Ciencias Farmacéuticas, División de Ciencias Biológicas y de la Salud, Universidad Autónoma Metropolitana – Unidad Xochimilco, Ciudad de México 04960, Mexico.

[b]Departamento de Sistemas Biológicos, División de Ciencias Biológicas y de la Salud, Universidad Autónoma Metropolitana – Unidad Xochimilco, Ciudad de México 04960, Mexico. [c]Área Académica de Química, Universidad Autónoma del Estado de Hidalgo, Hidalgo 42184, Mexico.

[d]Laboratorio de Biosensores y Modelaje Molecular, Departamento de Bioquímica, Facultad de Medicina, Universidad Nacional Autónoma de México, Ciudad de México 04510, Mexico.

[e]Laboratorio de Farmacología, Departamento de Ciencias de la Salud, D.C.B.S., Universidad Autónoma Metropolitana – Unidad Iztapalapa, Ciudad de México 09340, Mexico.

* Corresponding Author:

Francisco Cortés-Benítez: jcortesb@correo.xoc.uam.mx
Julio-César Almanza-Pérez: jcap@xanum.uam.mx
Martín González-Andrade: martin@bq.unam.mx

Table content

Figure S1. ^{1}H NMR (400 MHz) spectrum of **5a** in DMSO-d_6

Figure S2. ^{13}C NMR (101 MHz) spectrum of **5a** in DMSO-d_6

Figure S3. A) Full COSY spectra of **5a** in DMSO-d_6 and highfield spectra

Figure S4. A) Full HMBC spectra of **5a** in DMSO-d_6, low-field spectra and high-field spectra

Figure S5. A) Full HSQC spectra of **5a** in DMSO-d_6, low-field spectra and high-field spectra

Figure S6. ^{1}H NMR (400 MHz) spectrum of **5b** in DMSO-d_6

Figure S7. ^{13}C NMR (101 MHz) spectrum of **5b** in DMSO-d_6

Figure S8. A) Full COSY spectra of **5b** in DMSO-d_6 and highfield spectra.

Figure S9. A) Full HMBC spectra of **5b** in DMSO-d_6, low-field spectra, and high-field spectra

Figure S10. A) Full HSQC spectra of **5b** in DMSO-d_6 and highfield spectra

Figure S11. ^{1}H NMR (400 MHz) spectrum of **5c** in DMSO-d_6

Figure S12. ^{13}C NMR (101 MHz) spectrum of **5c** in DMSO-d_6

Figure S13. A) Full COSY spectra of **5c** in DMSO-d_6 and highfield spectra

Figure S14. A) Full HMBC spectra of **5c** in DMSO-d_6, low-field spectra and high-field spectra

Figure S15. A) Full HSQC spectra of **5c** in DMSO-d_6 and highfield spectra

Figure S16. ^{1}H NMR (400 MHz) spectrum of **5d** in DMSO-d_6

Figure S17. ^{13}C NMR (101 MHz) spectrum of **5d** in DMSO-d_6

Figure S18. A) Full COSY spectra of **5d** in DMSO-d_6 and highfield spectra

Figure S19. A) Full HMBC spectra of **5d** in DMSO-d_6, low-field spectra, and high-field spectra

Figure S20. A) Full HSQC spectra of **5d** in DMSO-d_6, low-field spectra and high-field spectra

Figure S21. Mass spectrum formula report of compound **5a**

Figure S22. Mass spectrum formula report of compound **5b**

Figure S23. Mass spectrum formula report of compound **5c**

Figure S24. Mass spectrum formula report of compound **5d**

Figure S25. Chromatogram of compound **5a**

Figure S26. Chromatogram of compound **5b**

Figure S27. Chromatogram of compound **5c**

Figure S28. Chromatogram of compound **5d**

Figure S29. Inhibition of $PTP1B_{1-285}$ for amide derivatives. (**A**) compound **5a**, **5b,** and **5d**

Figure S30. Selectivity of the inhibition for PTP1B over TCPTP for amide derivatives at 200 μM

Figure S31. Lineweaver–Burk plots for *h*$PTP1B_{1-400}$ inhibition

Table S1. Results of molecular docking simulations using the long form of $PTP1B_{1–400}$

Figure S32. 2D-diagram for the interactions performed by compounds **4**, **5a**-**5d** within the site 2 of $PTP1B_{1–400}$-pNPP complex.

Table S2. Average RMSD, RMSF, and Binding Energy values for 300 ns of MD simulations on the $PTP1B_{1-400}$-pNPP and $PTP1B_{1-400}$-pNPP-ligand systems

Table S3. Results of the physicochemical properties predicted SwissADME web tool

Figure S33. Physicochemical and pharmacokinetic properties of compound **4** calculated using the free web tool SwissADME

Figure S34. Physicochemical and pharmacokinetic properties of **5a** calculated using the free web tool SwissADME

Figure S35. Physicochemical and pharmacokinetic properties of **5b** calculated using the free web tool SwissADME

Figure S36. Physicochemical and pharmacokinetic properties of **5c** calculated using the free web tool SwissADME

Figure S37. Physicochemical and pharmacokinetic properties of **5d** calculated using the free web tool SwissADME

Figure S38. Effect of **GA**, **4**, and its amide derivatives (**5a**, **5b,** and **5d**) on cell functionality of C2C12 myoblasts

EXPERIMENTAL SECTION

Chemicals and Reagents

All reagents and starting materials were obtained from Sigma–Aldrich (Toluca, MEX, Mexico, and St. Louis, MO, USA). Reactions were monitored by thin-layer chromatography on 0.2 mm silica gel-coated 60 F254 plates (Sigma–Aldrich) and visualized under UV light. Melting points were determined using a Buchi Melting Point M-565 melting point apparatus without correction. ^{1}H, ^{13}C, and 2D NMR spectra were recorded on Bruker Ascend spectrometers (Bruker, Billerica, MA, USA) operating at 400 MHz for ^{1}H, and at 151 MHz for ^{13}C, respectively. Chemical shifts are reported in parts per million (ppm) relative to tetramethylsilane ($Me_4Si = 0$); coupling constants (*J* values) are expressed in Hertz (Hz). Multiple patterns are indicated as follows: s, singlet; d, doublet; q, quartet; dd, doublet of doublets; t, triplet; m, multiplet; bs, broad singlet. High-resolution mass spectra (HRMS) were obtained using a micrOTOF-ESI-TOF-MS mass spectrometer by direct infusion and in a positive mode using nitrogen (4 mL min^{-1}) as nebulizer gas, spray voltage (4.5 kV) at 150 °C, within a mass range of *m*/*z* 50−3000. The results are expressed as *m*/*z*. All data spectra are reported in the Supporting Information. According to IUPAC rules, compounds were named using the automatic generator tool implemented in ChemDraw Professional 22.0.0 software (PerkinElmer, Waltham, MA, USA). The purity of the compounds was determined by HPLC using an Agilent 1260 series chromatograph equipped with an autosampler, a thermostat, and a diode-array detector (DAD). Data for each peak, including area, retention time, symmetry (Symm), and peak purity factor (PP), were calculated using OpenLAB CDS software (Agilent Technologies®). The column used was an Eclipse XDB with octyl chains as the stationary phase (C8), with dimensions of 150 × 4.6 mm and a particle size of 5 µm. The mobile phase consisted of water (0.05% formic acid), acetonitrile (CH_3CN), and methanol (MeOH), in various ratios depending on the sample (see Table 1). The flow rate was set at 1.0 mL/min, the oven temperature was maintained at 25°C, and the injection volume was 5 µL. The absorbance of the compounds was measured at wavelengths (λ) of 230, 254, and 280 nm. Ultraviolet/visible (UV/Vis) spectra were recorded from 200 to 800 nm at an acquisition rate of 10 scans per second. For sample preparation, 2 mg of each compound was weighed and placed in 2 mL volumetric flasks. The compounds were dissolved in a minimal amount of mobile phase, and the samples were sonicated for 2 minutes. Following sonication, 100 µL of each sample was taken and diluted to 1 mL with the mobile phase, resulting in a final concentration of 0.1 mg/mL. The compound **5c** sample was prepared by dissolving it in a 50:50 mixture of acetone and acetonitrile, while a second aliquot was dissolved in the mobile phase. No unexpected or unusually high safety hazards were encountered

Synthesis

Compound 5a

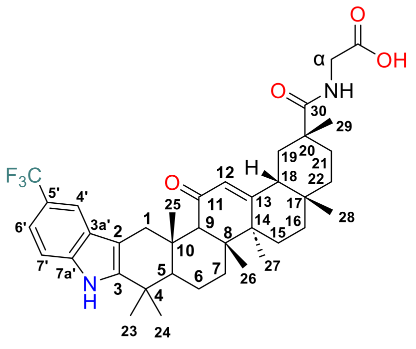

5a

A solution of compound **4** (100 mg, 0.163 mmol) and 1,1-carbonyldiimidazole (CDI) (80 mg, 0.49 mmol) in methylene chloride (5 mL) was stirred at room temperature for 30 minutes. Immediately, glycine methyl ester hydrochloride (61 mg, 0.49 mmol) and triethylamine (TEA) (27.6 µL, 0.164 mmol) were added to the mixture, and the resulting solution was stirred at room temperature overnight. Then, the mixture was precipitated in an ice-brine solution, and the obtained solid was hydrolyzed with KOH (5 eq.) in methanol at room temperature. The crude

product was purified by flash chromatography using methylene chloride/MeOH (95:5) to give 48.5 mg (44 %) of compound **5a**; m.p. 349-354 °C. ^{1}H NMR (400 MHz, DMSO-d$_6$): δ_H 12.37 (s, 1H, -COOH), 11.23 (s, 1H, NH (indole)), 7.91 (t, *J* = 5.8 Hz, 1H, NH (amide)), 7.58 (s, 1H, H-4'), 7.42 (d, *J* = 8.4 Hz, 1H, H-6'), 7.28 (d, *J* = 8.6 Hz, 1H, H-7') 5.68 (s, 1H, H-12), 3.80 (dd, *J* = 17.8, 5.3 Hz, 1H, H-α) 3.76 (d, *J* = 15.8 Hz, 1H, H-1), 3.67 (dd, *J* = 17.3, 5.5 Hz, 1H, H-α'), 2.67 (s, 1H, H-9), 2.28 (s, 1H, H-18), 2.26 (d, *J* = 15.3 Hz, 1H, H-1'), 2.11 (dd, *J* = 13.3, 9.6 Hz, 1H, H-16), 1.89-1.62 (m, 6H, H-19, H-15, H-7, H-21, H-19' and H-6), 1.59-1.44 (m, 4H, H-6', H-7', H-21'and H-22), 1.43 (s, 1H, H-5), 1.40 (s, 3H, CH$_3$-29), 1.31 (s, 3H, CH$_3$-24), 1.26 (m, 1H, H-22'), 1.23 (s, 3H, CH$_3$-23), 1.18 (m, 1H, H-15'), 1.12 (s, 3H, CH$_3$-27), 1.06 (s, 3H, CH$_3$-26), 1.05 (s, 3H, CH$_3$-25), 0.96 (d, *J* = 13.3 Hz, 1H, H-16'), 0.76 (s, 3H, CH$_3$-28). ^{13}C NMR (101 MHz, DMSO-d$_6$): δ_C 198.93 (C11), 175.76 (COOH), 171.66 (C13), 170.35 (C30), 143.59 (C3), 137.89 (C3a'), 127.64 (C12), 126.87 (C7a'), 124.50 (CF$_3$, *J* = 271.69 Hz), 118.58 (C5'), 116.52 (C7'), 114.45 (C4'), 111.08 (C6'), 106.15 (C2), 59.61 (C9), 52.22 (C5), 47.13 (C18), 44.82 (C20), 43.05 (C8), 42.78 (C14), 41.14 (C19), 40.88 (Cα), 37.49 (C10), 36.99 (C22), 36.66 (C1), 33.97 (C4), 31.44 (C17), 31.35 (C21), 30.52 (C7), 30.41 (C24), 28.51 (C26), 28.29 (C28), 26.21 (C15), 26.01 (C16), 22.94 (C29), 22.86 (C23), 18.04 (C6), 17.95 (C27), 15.75 (C25). ESI-MS for $C_{39}H_{50}F_3N_2O_4^+$ [M+H]$^+$: cal. 667.3718 found 667.3722. HPLC purity of 99.9% (retention time = 6.2 min, isocratic elution of 70% CH$_3$CN/ 30% aqueous formic acid 0.05%, Agilent Eclipse XDB-C8 column).

Compound 5b

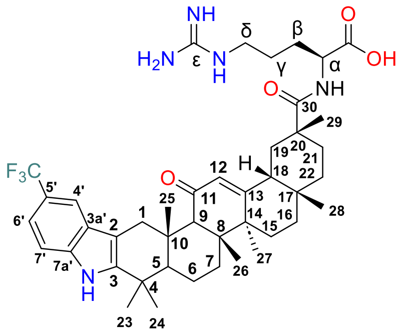

5b

A solution of compound **4** and CDI (80 mg, 0.49 mmol) in anhydrous THF (2 mL) was stirred at room temperature for 30 minutes. Immediately, L-arginine methyl ester dihydrochloride (128.5 mg, 0.49 mmol) and TEA (54 µL, 0.32 mmol) were added to the mixture, and the resulting solution was stirred at 120 °C overnight. Then the mixture was precipitated on ice-brine solution, and it was purified by flash chromatography using hexanes/ethyl acetate (from 7:3 to 6:4). Afterwards, the solid obtained was hydrolyzed with KOH (5 eq) in MeOH at room temperature and the reaction mixture was evaporated under reduced pressure and the product was precipitated with a HCl solution (10 %) and collected by filtration to give 47.7 mg (38 %) of compound **5b** as light yellow solid; m.p. >380 °C. ^{1}H NMR (400 MHz, DMSO-d$_6$): δ_H 12.58 (s, 1H, -COOH), 11.30 (s, 1H, NH (indole)), 7.81 (bs, 1H, NH guanidine group), 7.70 (d, *J* = 7.6 Hz, 1H, NH (amide)), 7.57 (s, 1H, H-4'), 7.43 (d, *J* = 8.4 Hz, 1H, H-6'), 7.29 (d, *J* = 8.6 Hz, 1H, H-7'), 7.0 (bs, 2H, NH$_2$ guanidine group), 5.74 (s, 1H, H-12), 4.23 (m, 1H, H-α), 3.75 (d, *J* = 15.8 Hz, 1H, H-1), 3.11 (m, 2H, H-δ and H-δ'), 2.67 (s, 1H, H-9), 2.27 (bs, 1H, H-18), 2.25 (d, *J* = 15.7 Hz, 1H, H-1'), 2.13 (m, 1H, H-16), 2.02-1.87 (m, 2H, H-19, H-15), 1.86-1.60 (m, 8H, H-β, H-15, H-7, H-21, H-19', H-6, H-6'and H-β'), 1.60-1.51 (m, 2H, H-7' and H-21'), 1.50 (m, 2H, H-γ and H-γ'), 1.41 (s, 3H, CH$_3$-29), 1.36 (m,1H, H-22), 1.31 (s, 3H, CH$_3$-24), 1.30-1.26 (m, 2H, H-22' and H-15'), 1.23 (s, 3H, CH$_3$-23), 1.13 (s, 3H, CH$_3$-27), 1.08 (s, 3H, CH$_3$-26), 1.05 (s, 3H, CH$_3$-25), 0.98 (d, *J* = 9.8 Hz, 1H, H-16'), 0.75 (s, 3H, CH$_3$-28). ^{13}C NMR (101 MHz, DMSO-d$_6$): δ_C 199.00 (C11), 175.45 (COOH), 173.98 (C13), 170.24 (C30), 156.88 (Cε), 143.65 (C3), 137.90 (C3a'), 127.62 (C12), 126.83 (C7a'), 124.52 (CF$_3$, *J* = 271.98 Hz), 118.52 (C5'), 116.52 (C7'), 114.46 (C4'), 111.11 (C6'), 106.07 (C2), 59.61 (C9), 52.18 (C5), 51.45 (Cα), 47.31 (C18), 44.84 (C20), 43.03 (C8), 42.86 (C14), 40.88 (C19), 40.31 (Cδ), 37.46 (C10), 37.19 (C22), 36.60 (C1), 33.97 (C4), 31.47 (C17), 31.37 (C7), 30.64 (C21), 30.44 (C24), 28.39 (C26), 28.34 (C28), 27.84 (Cβ), 26.27 (C15), 25.93 (C16), 25.42 (Cγ), 22.91 (C29), 22.87 (C23), 18.05 (C6), 17.96 (C27), 15.75 (C25). ESI-MS for $C_{43}H_{59}F_3N_5O_4^+$ [M + H]$^+$ calc. 766.4514 found 766.4510. HPLC purity of 99.9% (retention time = 2.1 min, isocratic elution of 50% CH$_3$CN/35 MeOH/15% aqueous formic acid 0.05%, Agilent Eclipse XDB-C8 column).

Compound 5c

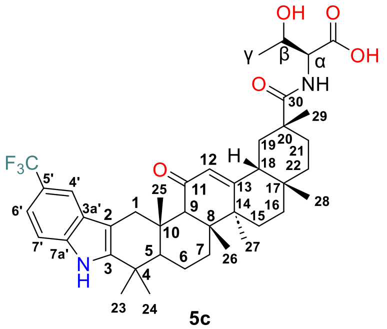

A solution of **4** in THF anhydrous (2 mL) and CDI (83.5 mg, 0.49 mmol) was stirred at room temperature for 30 minutes. Immediately, L-threonine methyl ester hydrochloride (61 mg, 0.49 mmol) and TEA (27.6 µL, 0.164 mmol) were added to the resulting solution, which was stirred at 120°C overnight. Then the mixture was precipitated on ice-brine solution, and it was purified by flash chromatography using hexanes/ethyl acetate (from 7:3 to 6:4). Afterwards, the solid obtained was hydrolyzed with KOH (5 eq) in MeOH at room temperature and the reaction mixture was evaporated under reduced pressure and the product was precipitated with a HCl solution (10 %) and collected by filtration for to give 85.6 mg (74 %) of compound **5c** as a light yellow solid; m.p. 284-286 °C. ^{1}H NMR (400 MHz, DMSO-d$_6$): δ_H 11.35 (s, 1H, NH (indole)), 7.55 (s, 1H, H-4'), 7.43 (d, J = 8.4 Hz, 1H, H-6'), 7.28 (d, J = 8.5 Hz, 1H, H-7'), 6.99 (d, J = 6.1 Hz, 1H, NH (amide)), 5.79 (s, 1H, H-12), 4.08 – 3.98 (m, 1H, H-β), 3.95 (m, 1H, H-α), 3.75 (d, J = 15.5 Hz, 1H, H-1), 2.68 (s, 1H, H-9), 2.26 (d, J = 15.2 Hz, 1H, H-1'), 2.24 (s, 1H, H-18), 2.18 – 2.07 (m, 1H, H-16), 1.81 (m, 1H, H-7), 1.78 (s, 3H, CH_3-γ), 1.75 – 1.45 (m, 6H, H-19, H-15, H-21, H-6, H-6' and H-21), 1.40 (s, 3H, CH_3-29), 1.39 – 1.35 (m, 2H, H-5 and H-7'), 1.31 (s, 3H, CH_3-24), 1.30 (m, 2H, H-22 and H-22'), 1.23 (s, 3H, CH_3-23), 1.19 (m, 1H, H-15'), 1.12 (s, 3H, CH_3-27), 1.07 (s, 3H, CH_3-26), 1.04 (s, 3H, CH_3-25), 0.95 (d, J = 6.2 Hz, 1H, H-16'), 0.76 (s, 3H, CH_3-28). ^{13}C NMR (101 MHz, DMSO-d$_6$): δ_C 199.46 (C11), 174.47 (COOH), 173.98, (C13), 170.45 (C30), 144.08 (C3), 138.36 (3a'), 128.16 (C12), 127.28 (C7a'), 124.96 (CF_3, J = 271.69 Hz), 119.28 (C5'), 116.93 (C7'), 114.84 (C4'), 111.59 (C6'), 106.52 (C2), 66.63 (Cβ), 60.06 (C9), 57.77 (Cα) , 52.65 (C5), 47.89 (C18), 45.28 (C20), 43.52 (C14), 43.48 (C8), 41.54 (C19), 37.90 (C10), 37.74 (C22), 37.09 (C1), 34.43 (C4), 31.99 (C17), 31.81 (C21), 31.17 (C7), 30.87 (C24), 29.38 (C26), 28.95 (C28), 26.63 (C15), 26.32 (C16), 23.43 (Cγ), 23.32 (C29 and C23), 18.40 (C27 and C6), 16.21 (C25).). ESI-MS for $C_{41}H_{54}F_3N_2O_5^+$ [M + H]$^+$ calc. 711.3979 found 711.3979. Diastereomeric ratio by HPLC = 16.4:83.6%. HPLC purity of 99.4:98.5% (retention time = 5.3 and 5.8 min, isocratic elution of 70% CH_3CN/30% aqueous formic acid 0.05%, Agilent Eclipse XDB-C8 column).

Compound 5d

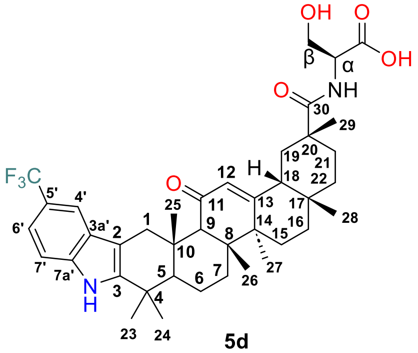

A solution of **4** and CDI (83.5 mg, 0.49 mmol) in THF anhydrous (2 mL) was stirred at room temperature for 30 minutes. Immediately, L-serine methyl ester hydrochloride (74.5 mg, 0.49 mmol) and TEA (27.6 µL, 0.164 mmol) were added to the resulting solution, and it was stirred at 120°C overnight. Then the mixture was precipitated from an ice-brine solution, and the resulting solid was hydrolyzed with KOH (5 eq) in MeOH at room temperature. The crude product was purified by flash chromatography using methylene chloride/MeOH (95:5) to give 23.5 mg (20%)

of compound **5d** as a light yellow solid; m.p. 291-296 °C. ^{1}H NMR (400 MHz, DMSO-d$_6$): δ_H 11.26 (s, 1H, NH (indole)), 7.56 (s, 1H, H-4'), 7.43 (d, *J* = 8.4 Hz, 1H, H-6'), 7.28 (d, *J* = 7.7 Hz, 1H, H-7'), 7.22 (d, *J* = 6.1 Hz, 1H, NH (amide)), 5.77 (s, 1H, H-12), 4.56 (bs, 1H, OH), 4.06 – 3.96 (m, 1H, H-α), 3.76 (d, *J* = 15.4 Hz, 1H, H-1), 3.67 (dd, *J* = 9.9, 4.9 Hz, 1H, H-β), 3.51 (dd, *J* = 10.1, 6.1 Hz, 1H, H-β'), 2.67 (s, 1H, H-9), 2.30 (m, 1H, H-18), 2.25 (d, *J* = 16.3 Hz, 1H, H-1'), 2.12 (m, 1H, H-16), 1.85 (d, *J* = 8.8 Hz, 1H, H-21), 1.78 (d, *J* = 12.7 Hz, 1H, H-15), 1.77 (m, 1H, H-7), 1.71 (m, 1H, H-19), 1.67 (m, 1H, H-6), 1.46-1.44 (m, 2H, H-6 and H-7'), 1.43 (s, 1H, H-5), 1.41, (s, 3H, H-29), 1.35 (m, 1H, H-21), 1.33 (m, 1H, H-22), 1.31 (s, 3H, CH_3-24), 1.29 (m, 1H, H-6), 1.28 (m, 1H, H-22'), 1.22 (s, 3H, CH_3-23), 1.18 (m, 1H, H-15'), 1.11 (s, 3H, CH_3-27), 1.08 (s, 3H, CH_3-26), 1.04 (s, 3H, CH_3-25), 0.97 (d, *J* = 10.9 Hz, 1H, H-16'), 0.76 (s, 3H, CH_3-28). ^{13}C NMR (101 MHz, DMSO) δ 198.97 (C11), 174.66 (C13), 173.47 (COOH), 170.09 (C30), 143.60 (C3), 137.90 (C3a'), 127.20 (C12), 126.85 (C7a'), 124.50 (CF_3 *J*= 272.15 Hz), 118.56 (C5'), 116.51 (C7'), 114.43 (C4'), 111.11 (C6'), 106.13 (C2), 62.25 (Cβ), 59.61 (C9), 54.74 (Cα), 52.21 (C5), 47.26 (C18), 44.83 (C20), 43.01 (C8), 42.95 (C14), 41.09 (C19), 37.46 (C10), 37.22 (C22), 36.64 (C1), 33.97 (C4), 31.51 (C17), 31.37 (C7), 30.76 (C21), 30.41 (C24), 28.76 (C26), 28.42 (C28), 26.20 (C15), 25.91 (C16), 22.97 (C29), 22.86 (C23), 18.04 (C6) 17.94 (C27), 15.75 (C25). ESI-MS for $C_{40}H_{52}F_3N_2O_5^+$ $[M+H]^+$: cal. 697.3823 found 697.3825. Diastereomeric ratio by HPLC = 18.5:81.5%. HPLC purity of 99.9:99.8% (retention time = 10.0 and 10.7 min, isocratic elution of 70% CH_3CN/30% aqueous formic acid 0.05%, Agilent Eclipse XDB-C8 column).

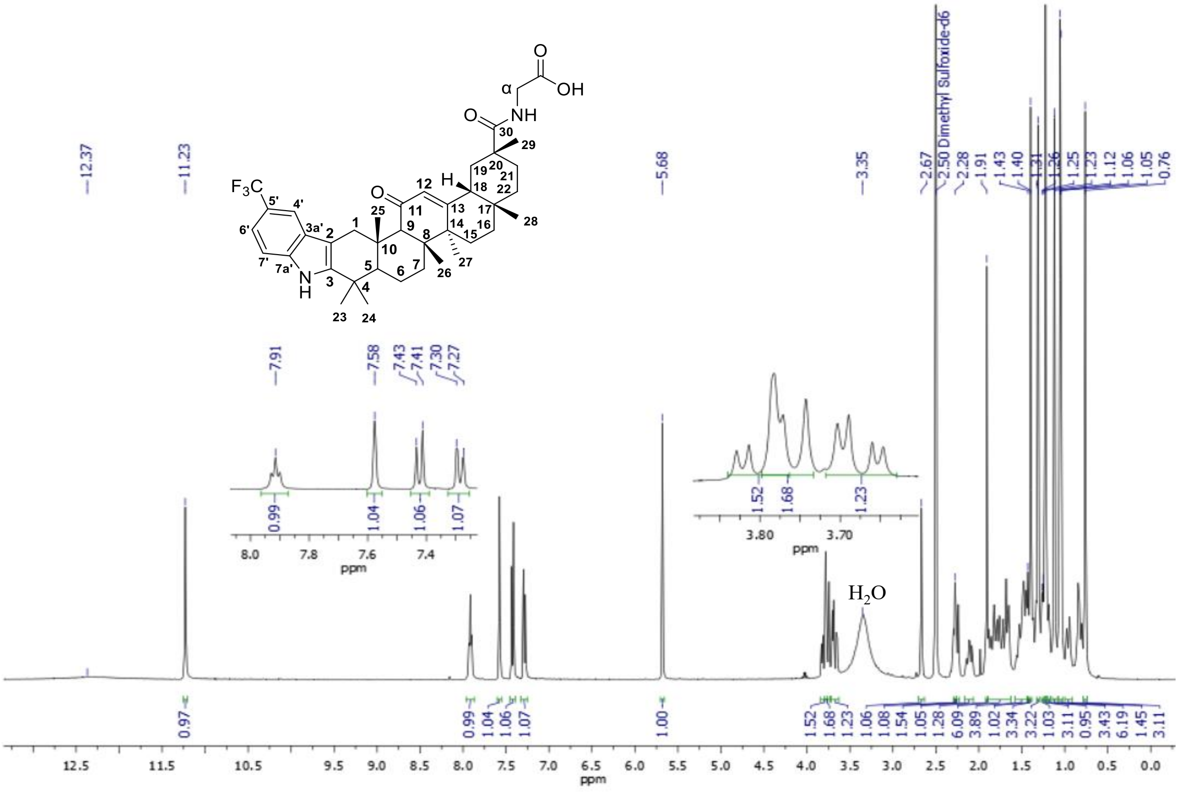

Figure S1. ^{1}H NMR (400 MHz) spectrum of **5a** in DMSO-d$_6$

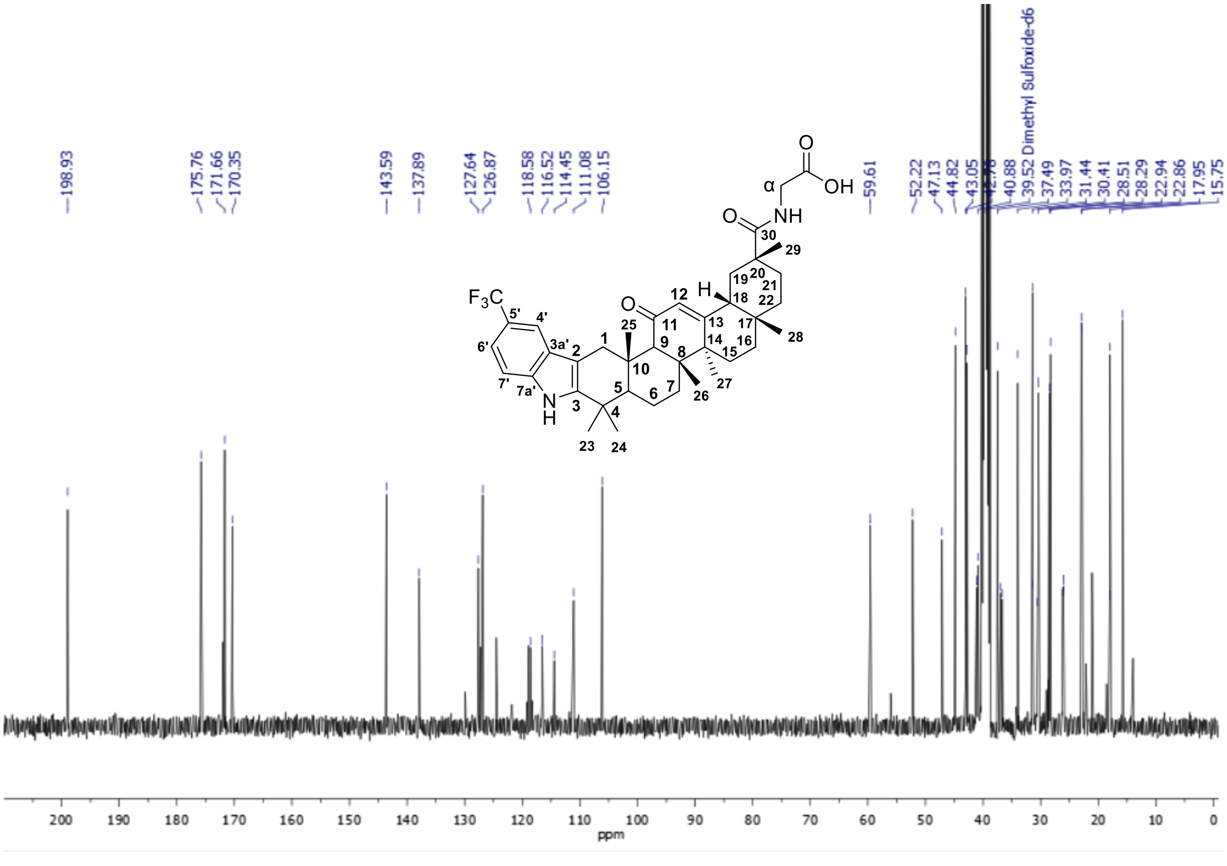

Figure S2. ^{13}C NMR (101 MHz) spectrum of **5a** in DMSO-d$_6$

A

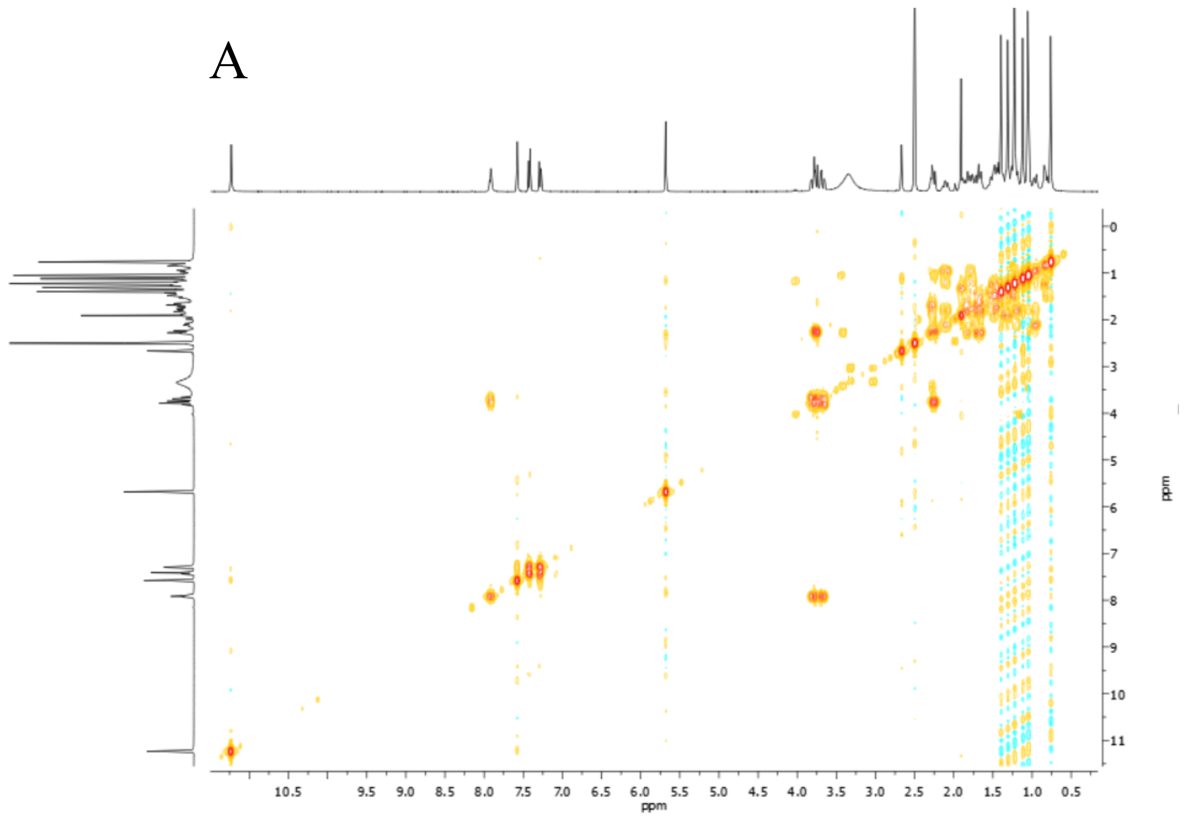

B

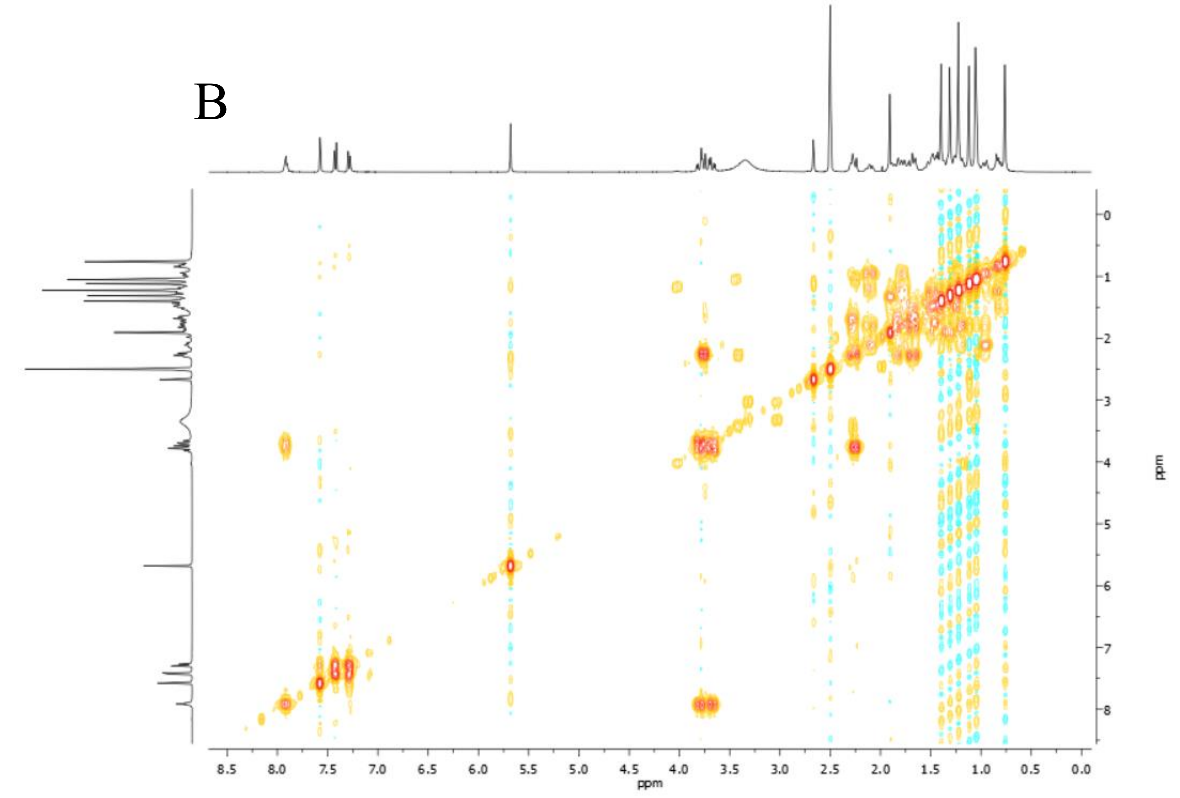

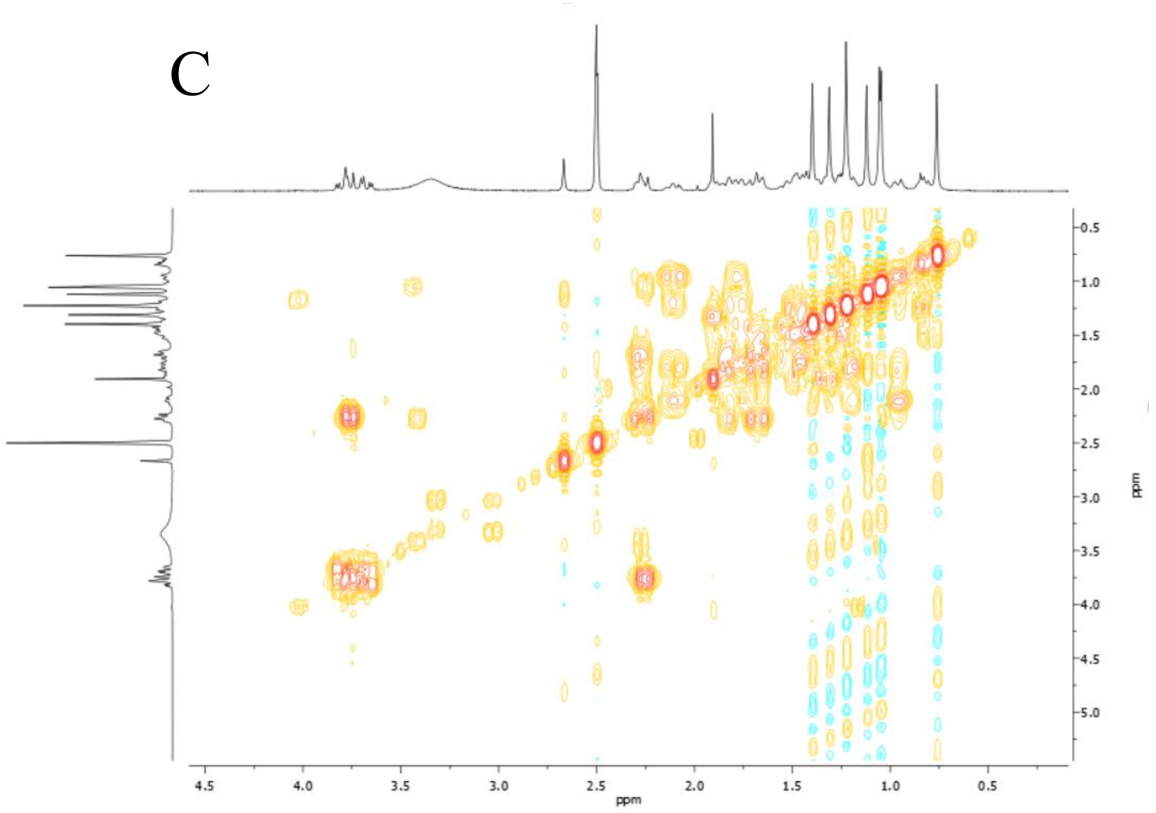

Figure S3. A) Full COSY spectra of **5a** in DMSO-d_6. **B)** and **C).** Highfield spectra.

A

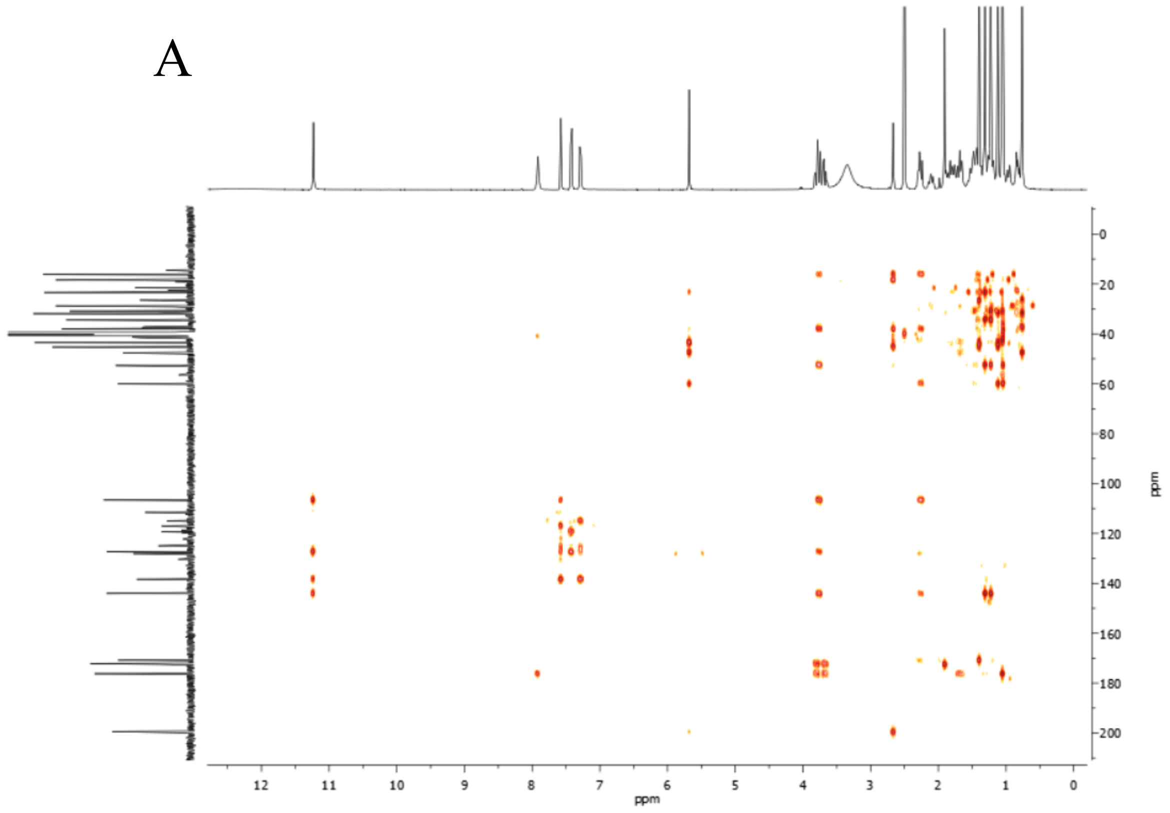

ppm
12
11
10
9
8
7
6
5
4
3
2
1
0
20
40
60
80
100
120
140
160
180
200

B

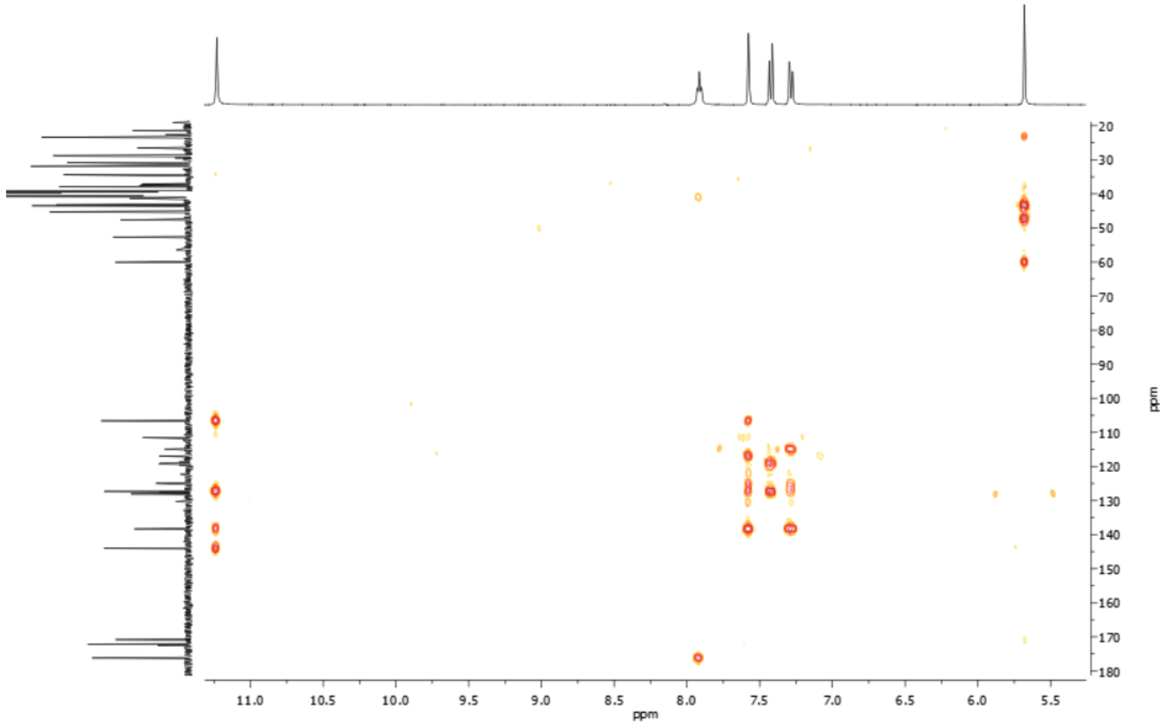

ppm
11.0
10.5
10.0
9.5
9.0
8.5
8.0
7.5
7.0
6.5
6.0
5.5
20
30
40
50
60
70
80
90
100
110
120
130
140
150
160
170
180

C

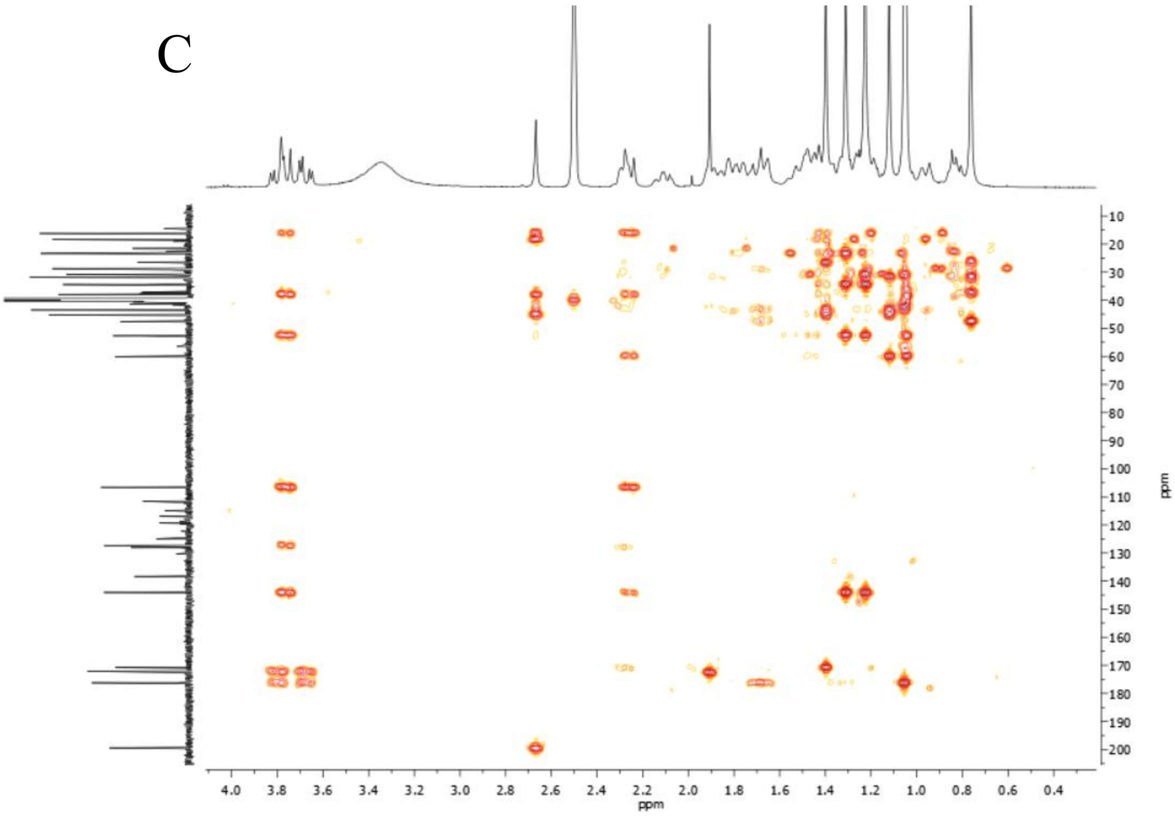

Figure S4. A) Full HMBC spectra of **5a** in DMSO-d$_6$. **B)** low-field spectra and **C).** Highfield spectra.

A

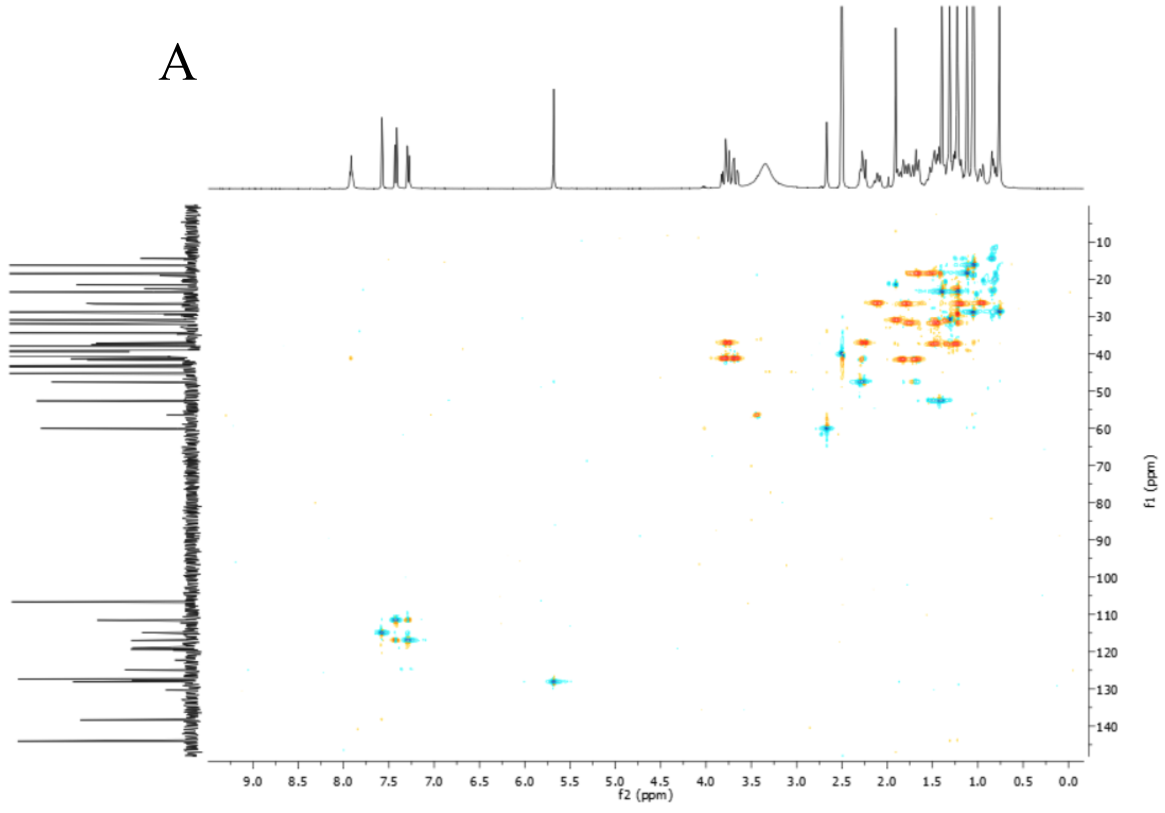

B

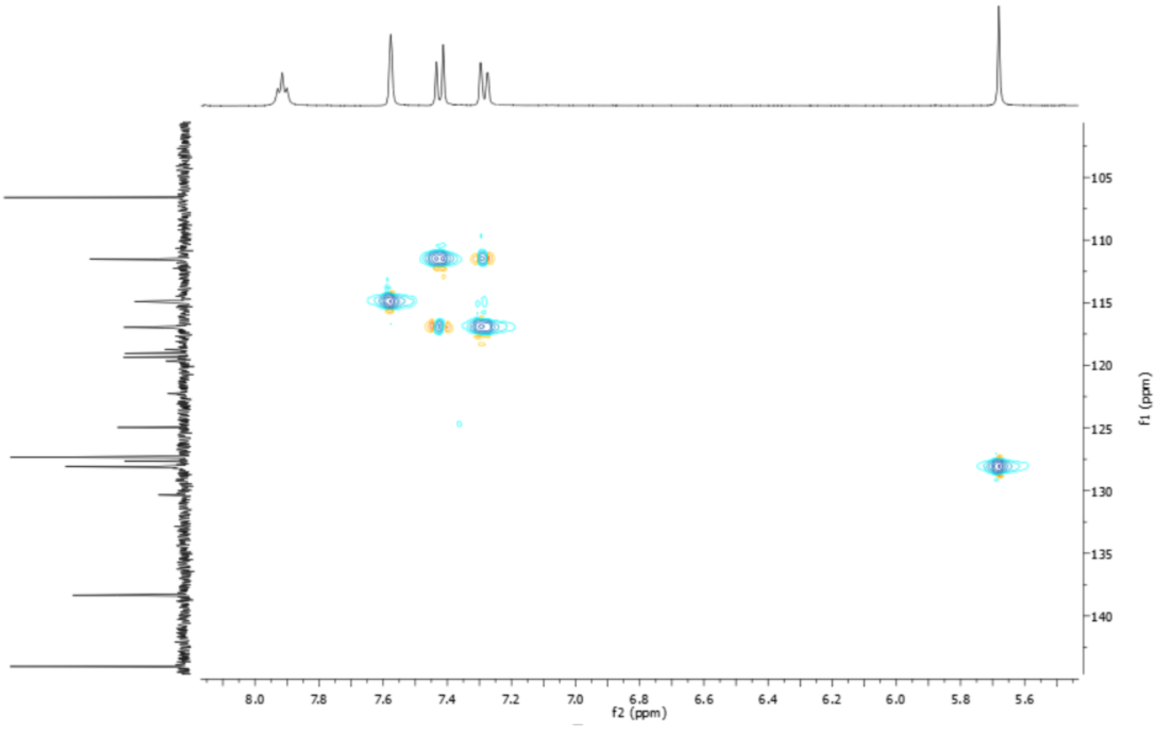

C

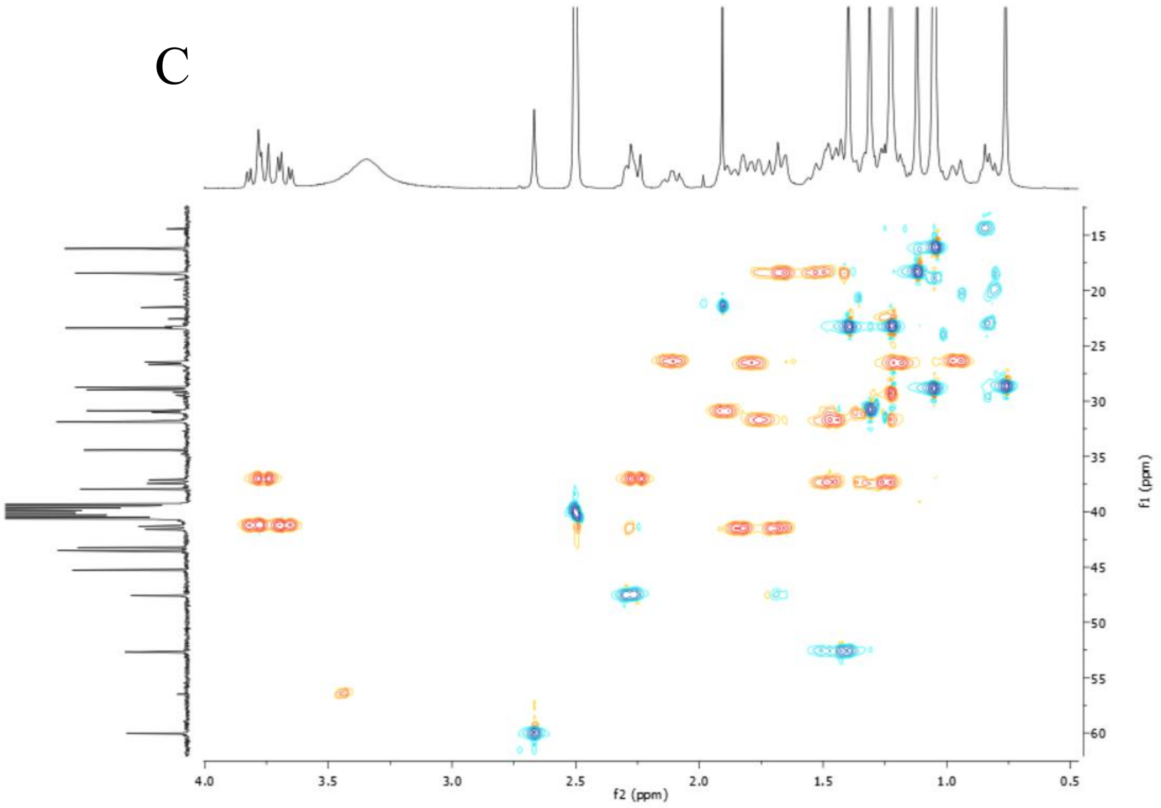

Figure S5. A) Full HSQC spectra of **5a** in DMSO-d_6. **B)** low-field spectra and **C).** Highfield spectra.

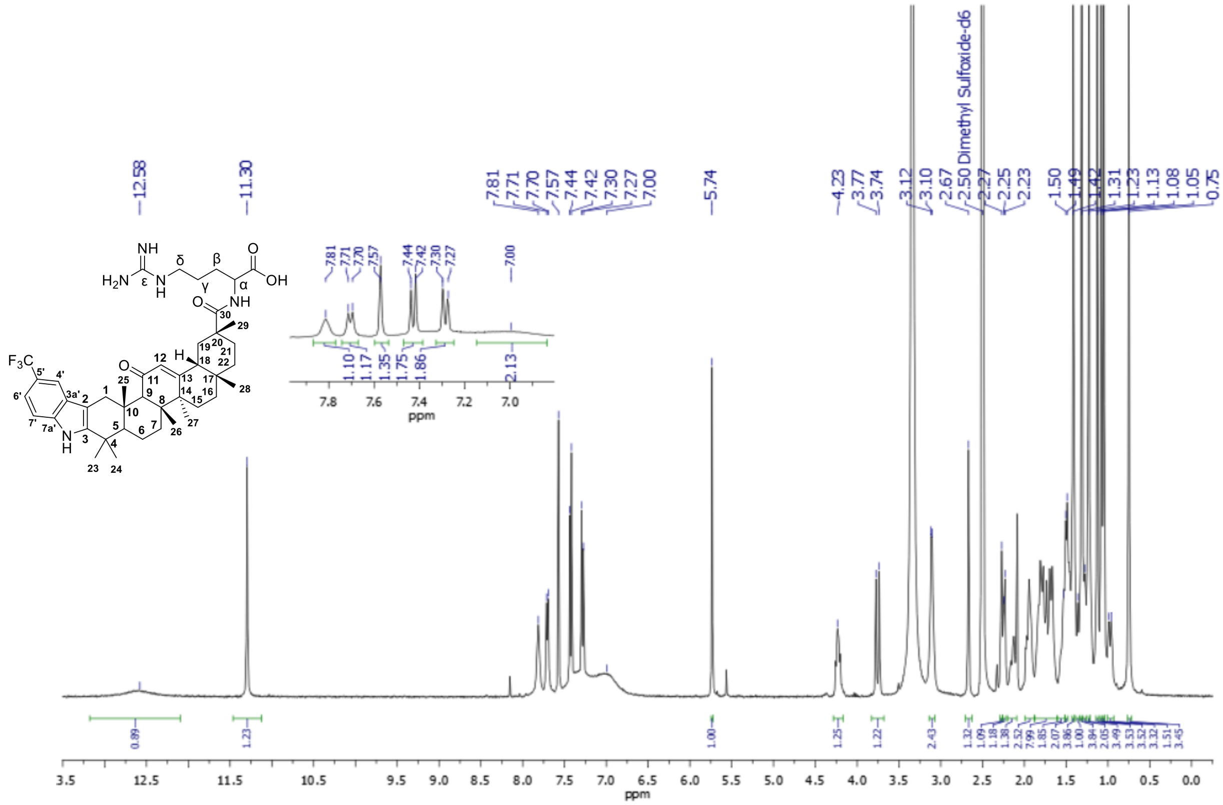

Figure S6. ^{1}H NMR (400 MHz) spectrum of **5b** in DMSO-d_6

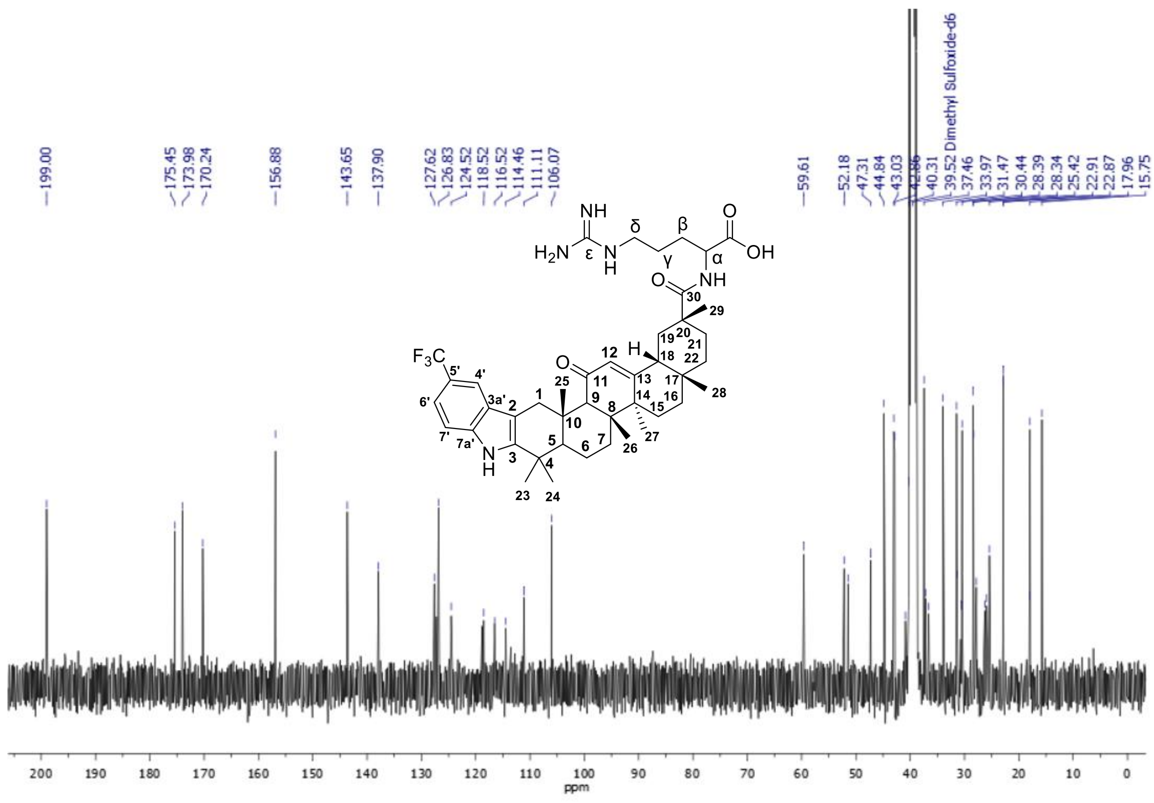

Figure S7. ^{13}C NMR (101 MHz) spectrum of **5b** in DMSO-d_6

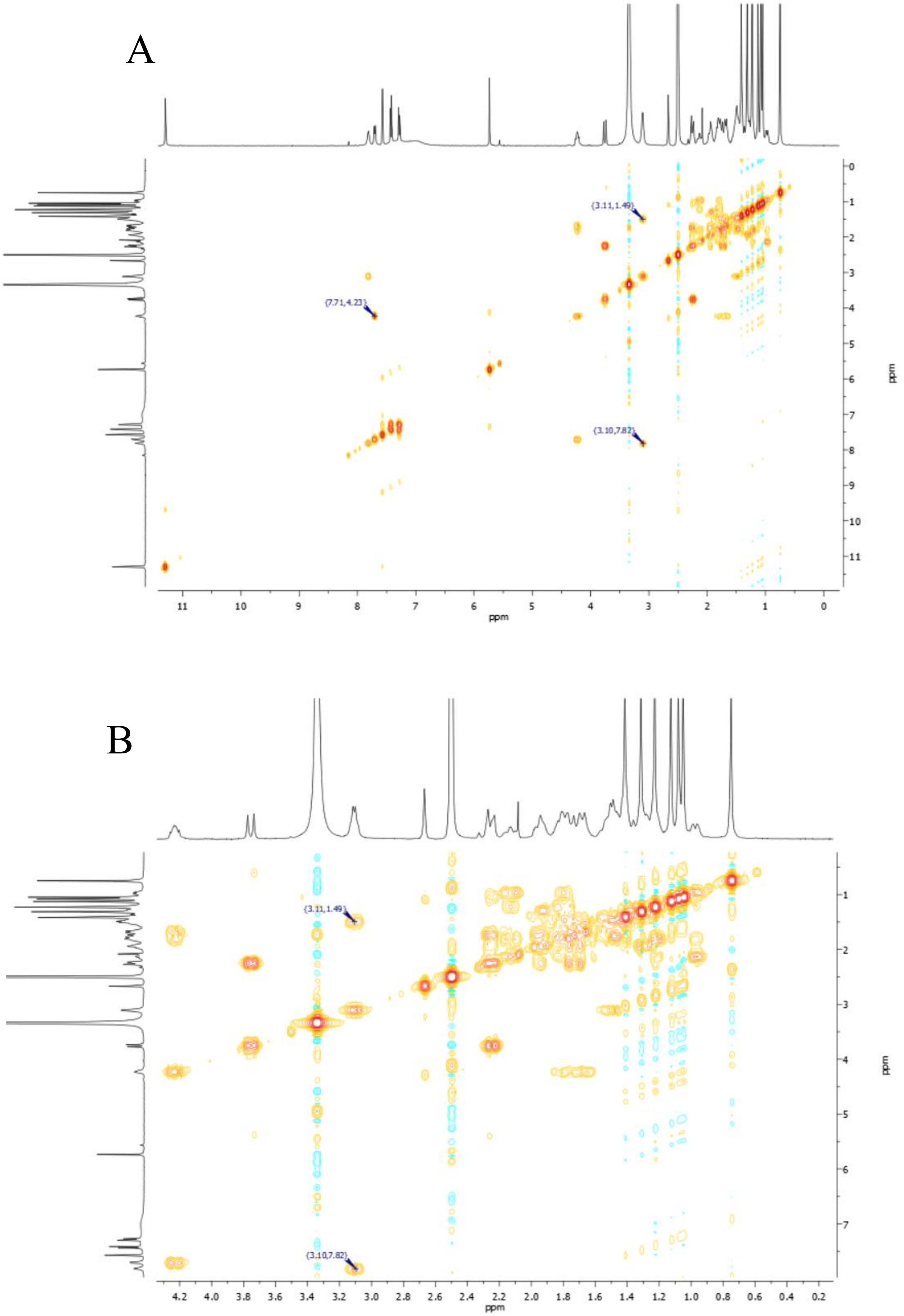

Figure S8. A) Full COSY spectra of **5b** in DMSO-d_6. **B)** Highfield spectra.

A

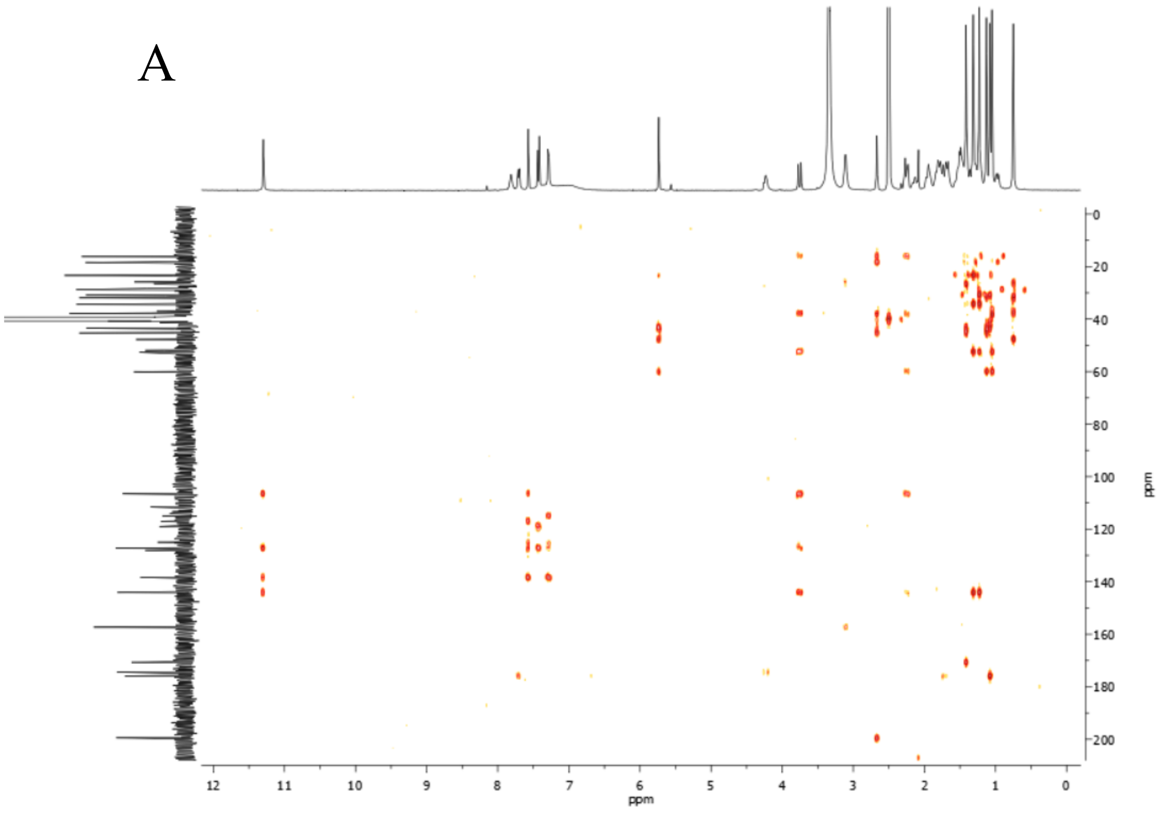

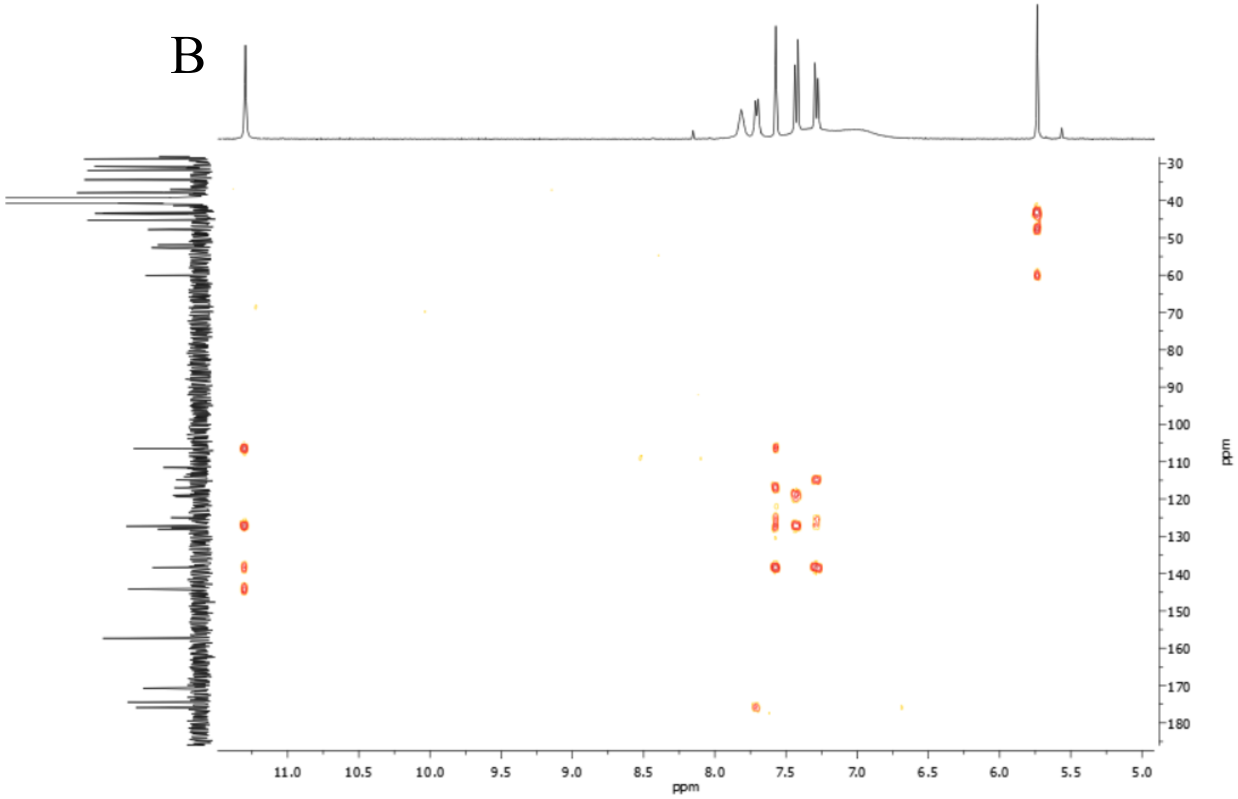

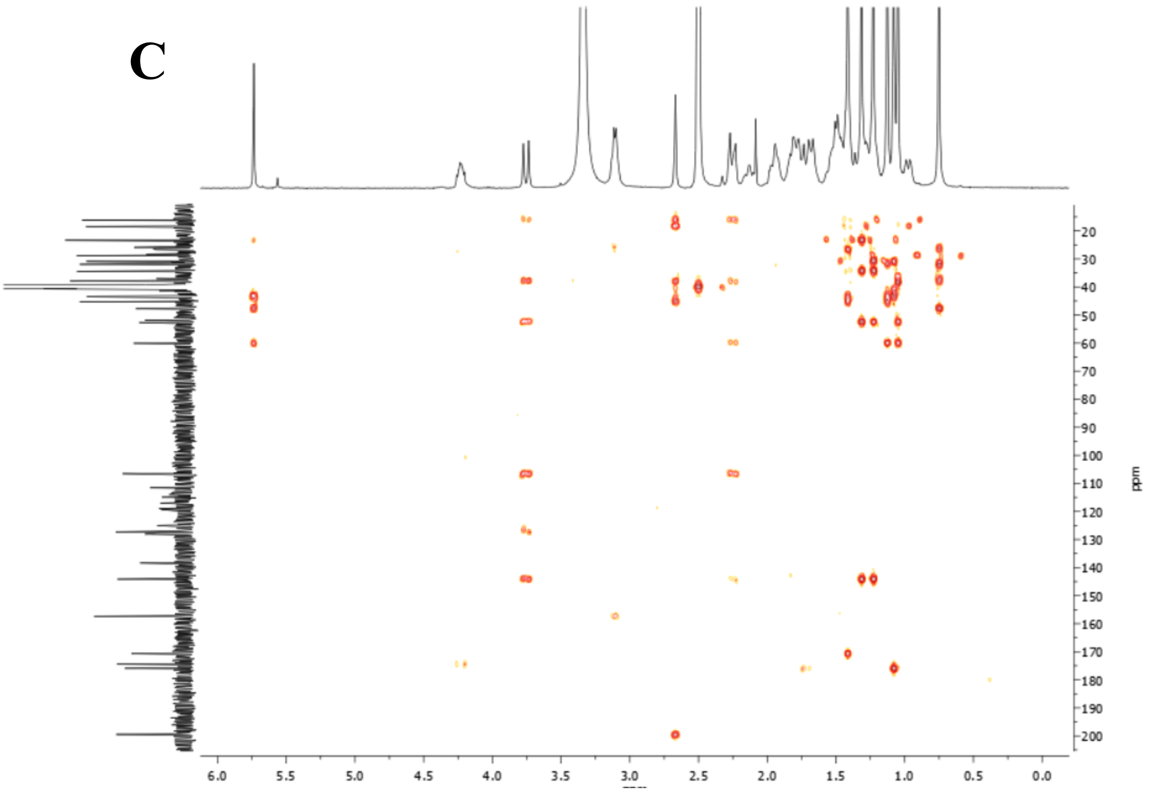

Figure S9. A) Full HMBC spectra of **5b** in DMSO-d_6. **B)** low-field spectra and **C).** Highfield spectra.

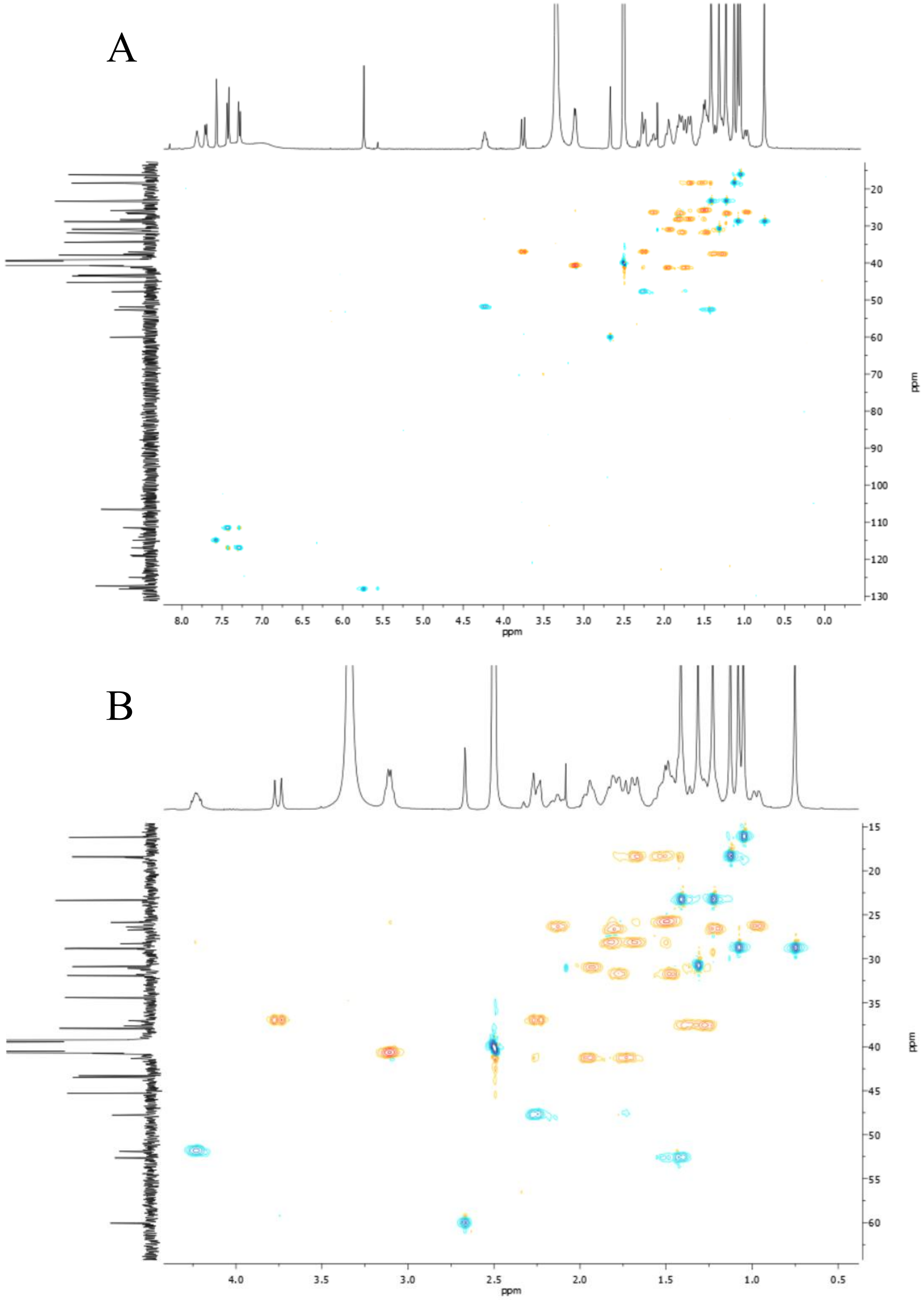

Figure S10. A) Full HSQC spectra of **5b** in DMSO-d$_6$. **B).** Highfield spectra.

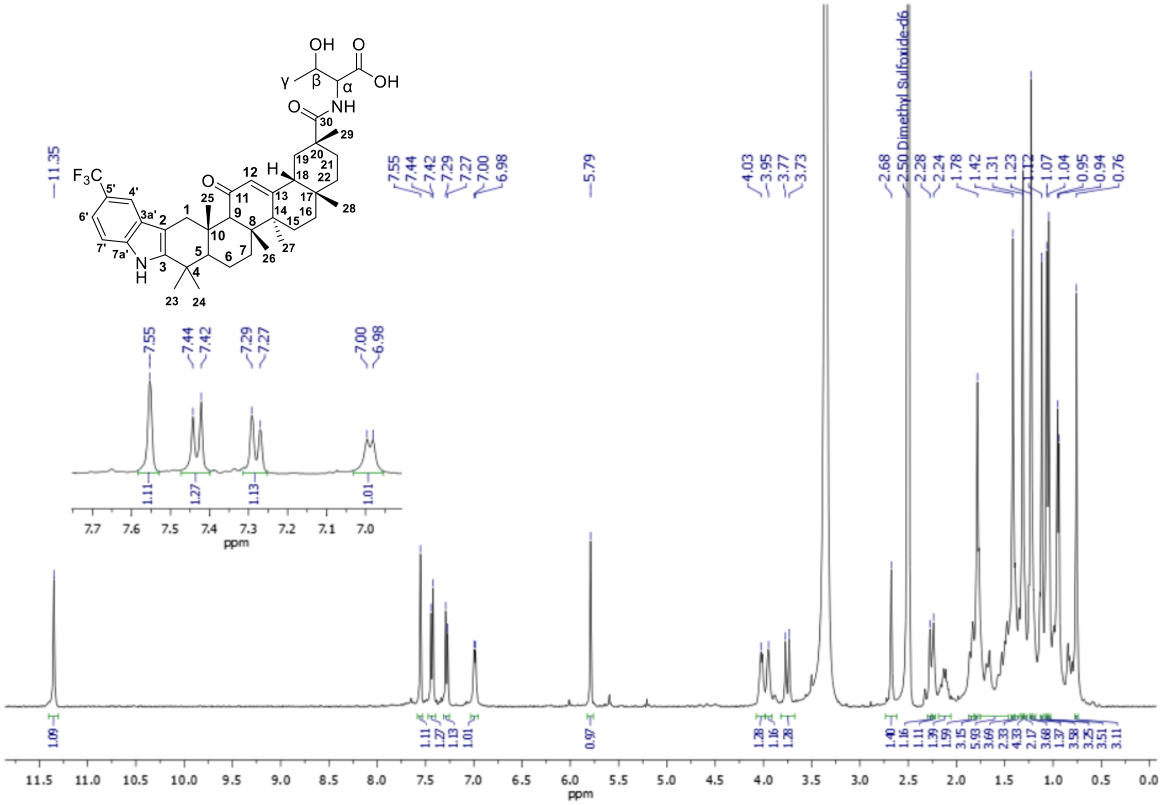

Figure S11. ^{1}H NMR (400 MHz) spectrum of **5c** in DMSO-d$_6$

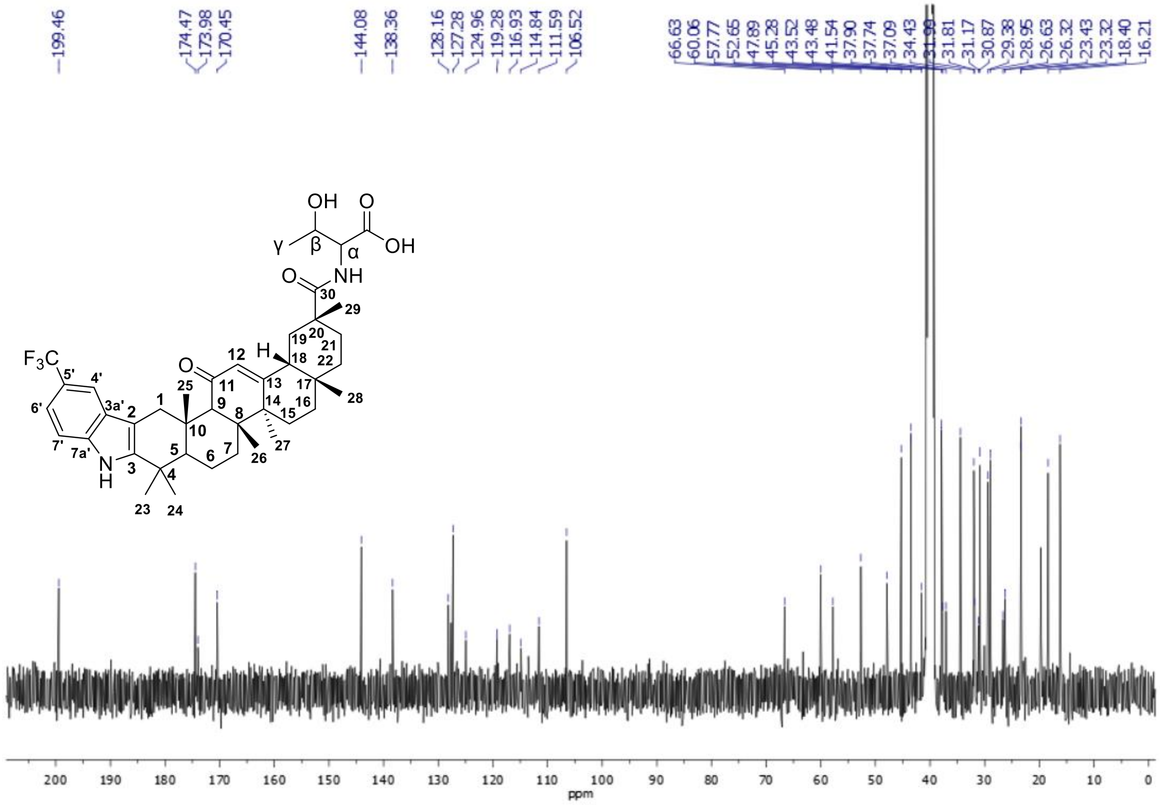

Figure S12. ^{13}C NMR (101 MHz) spectrum of **5c** in DMSO-d$_6$

A

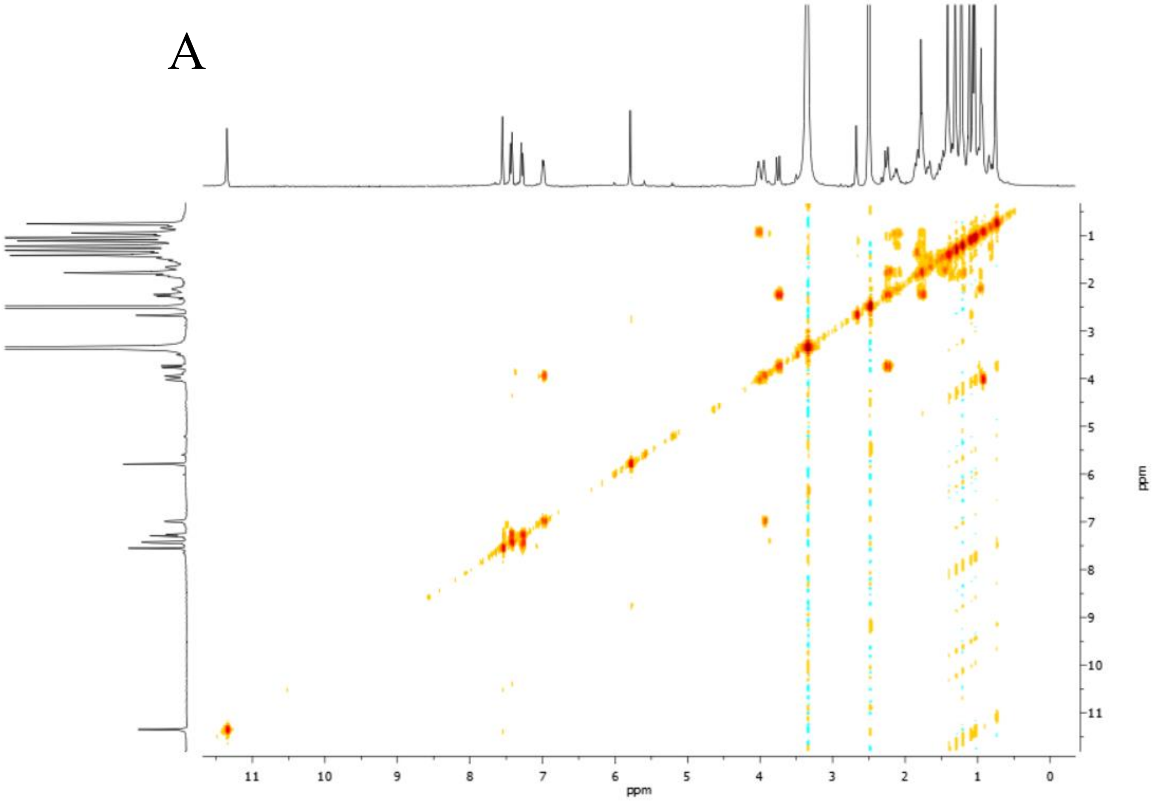

B

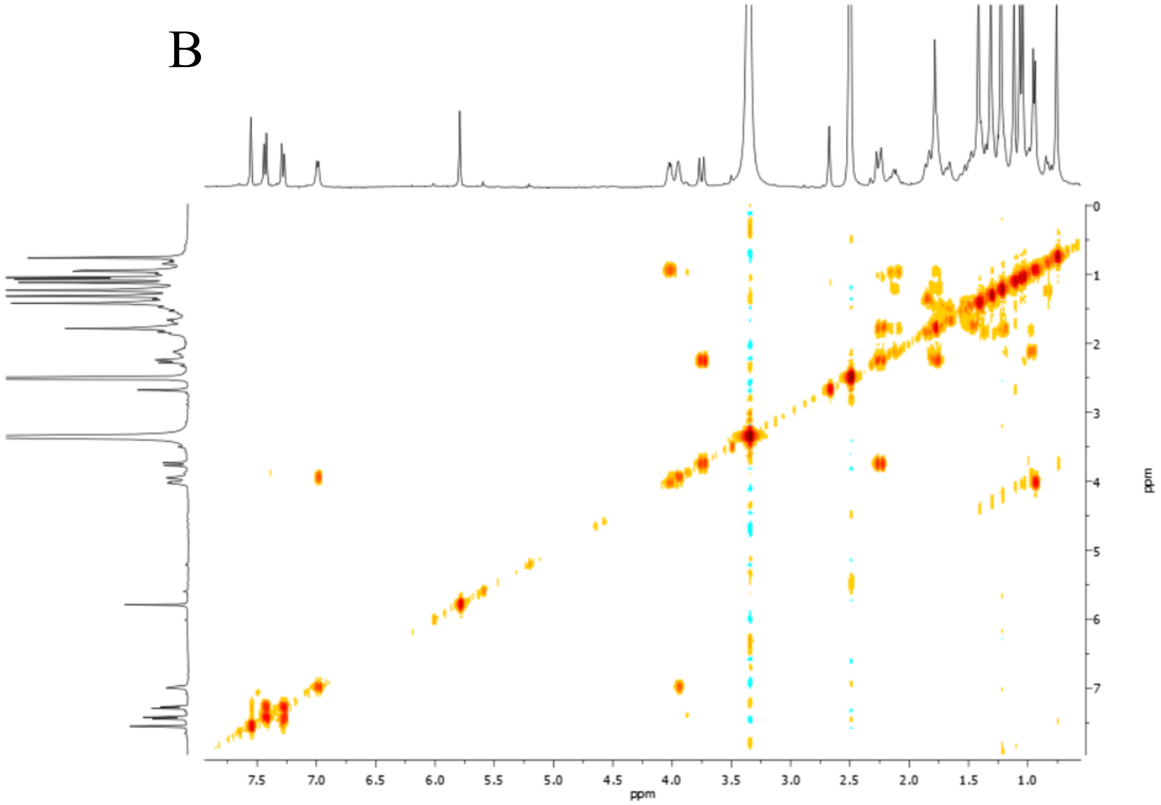

Figure S13. A) Full COSY spectra of **5c** in DMSO-d_6. **B)** Highfield spectra.

A

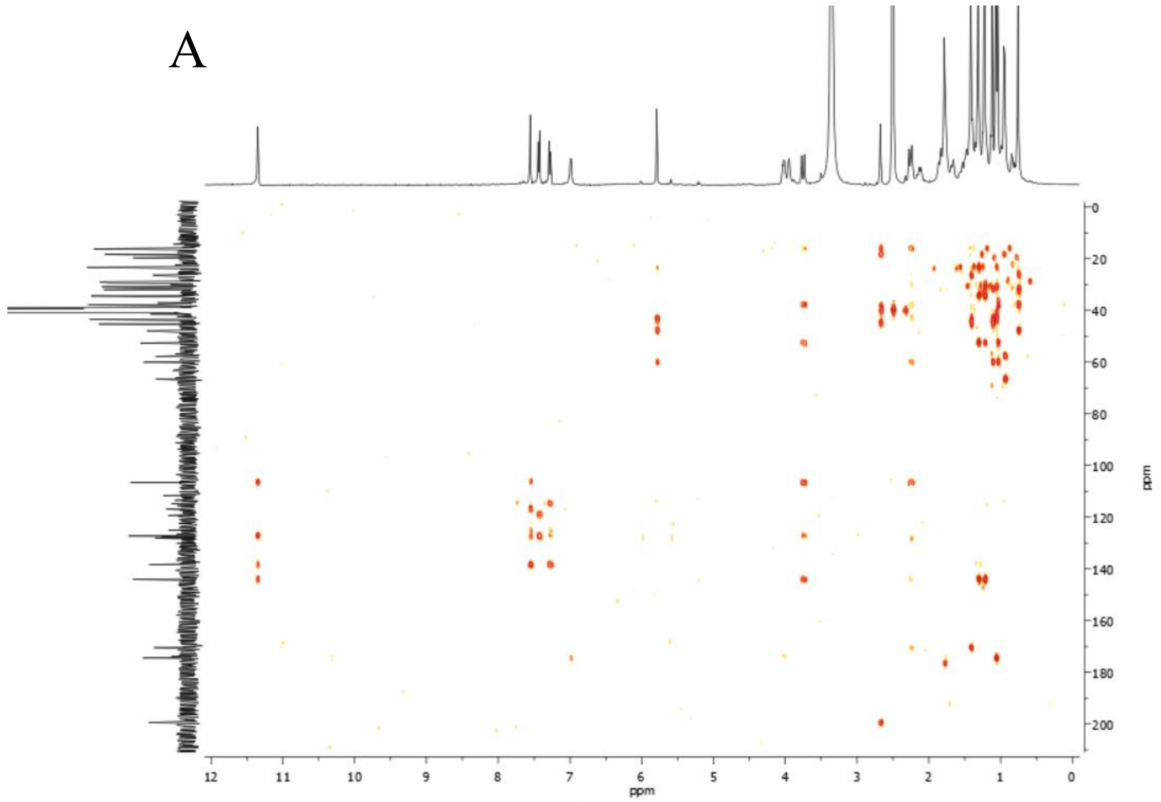

B

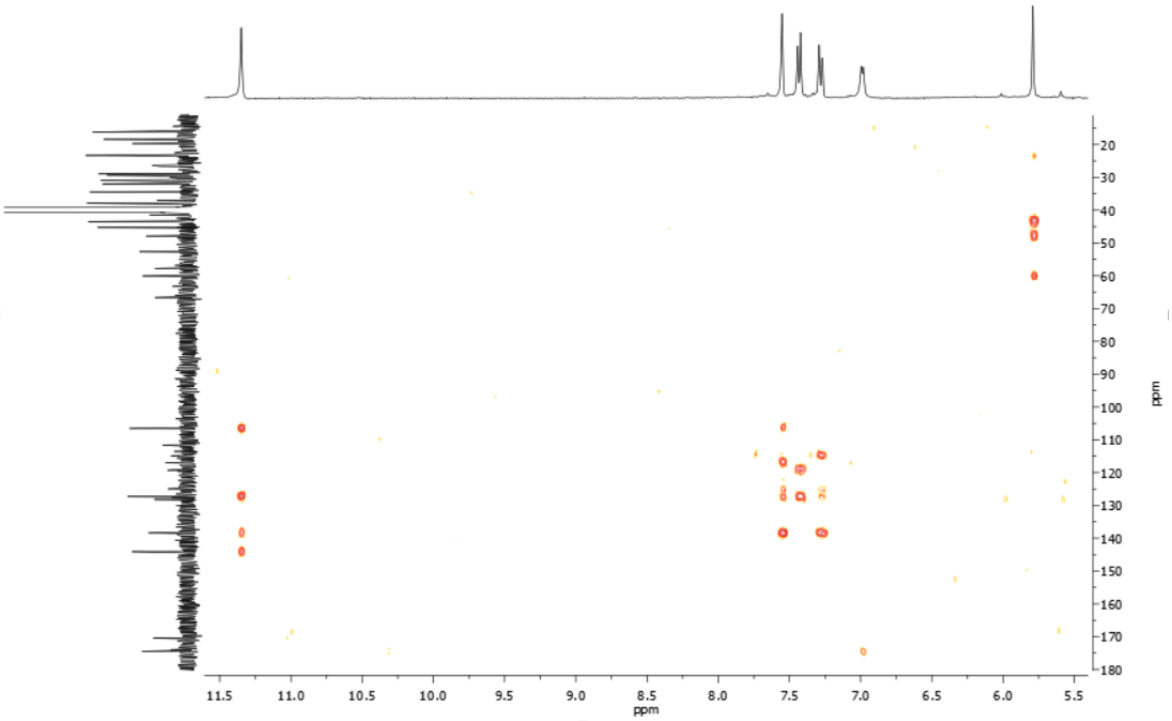

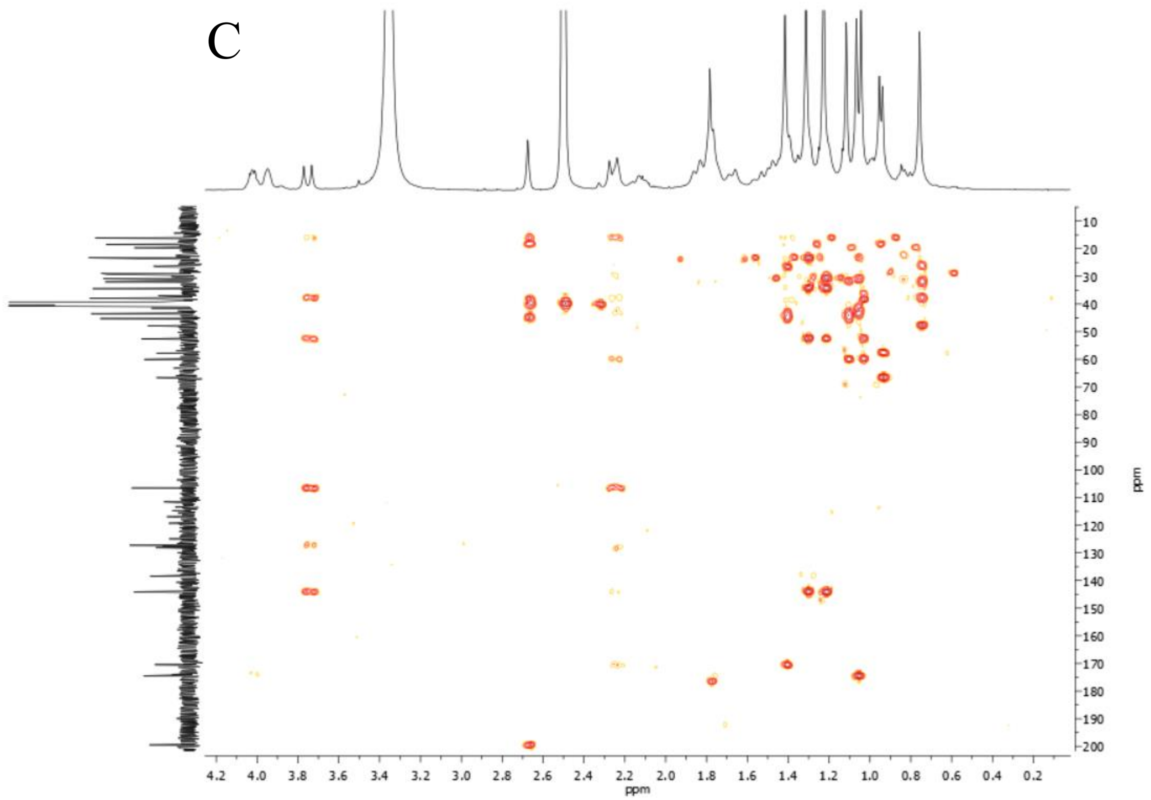

Figure S14. A) Full HMBC spectra of **5c** in DMSO-d_6. **B)** low-field spectra and **C).** Highfield spectra.

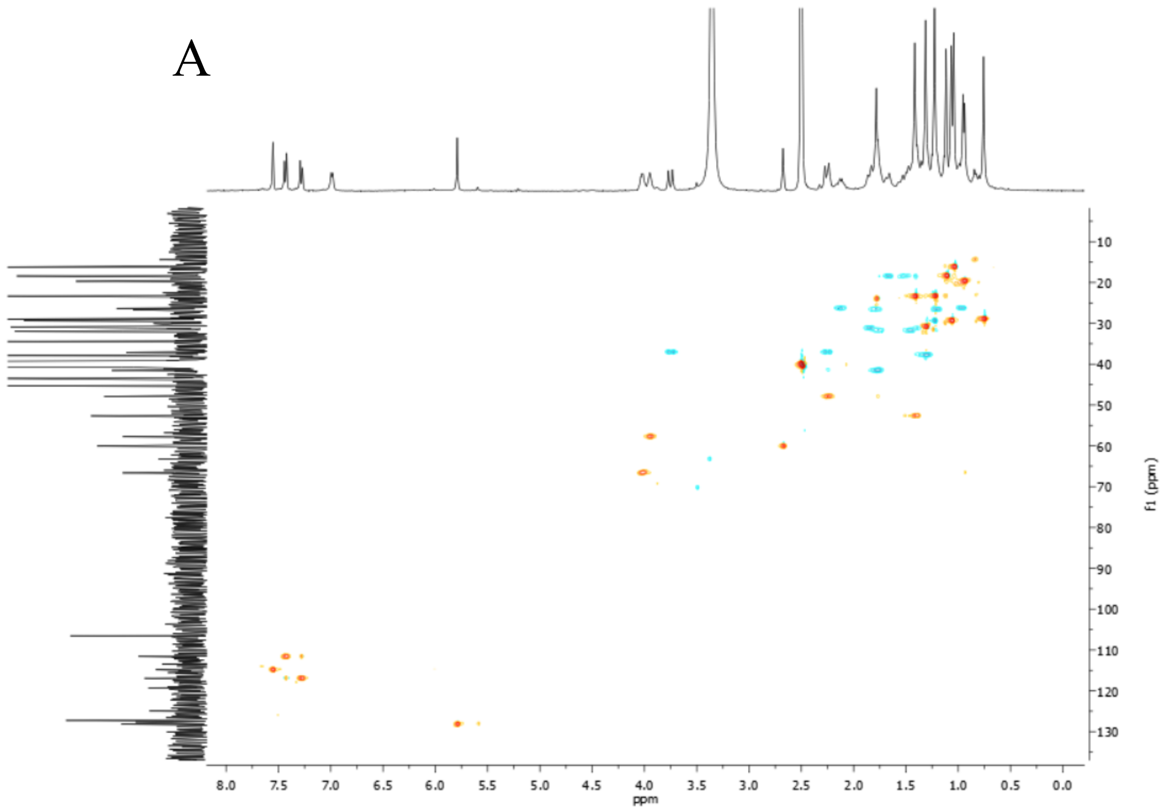

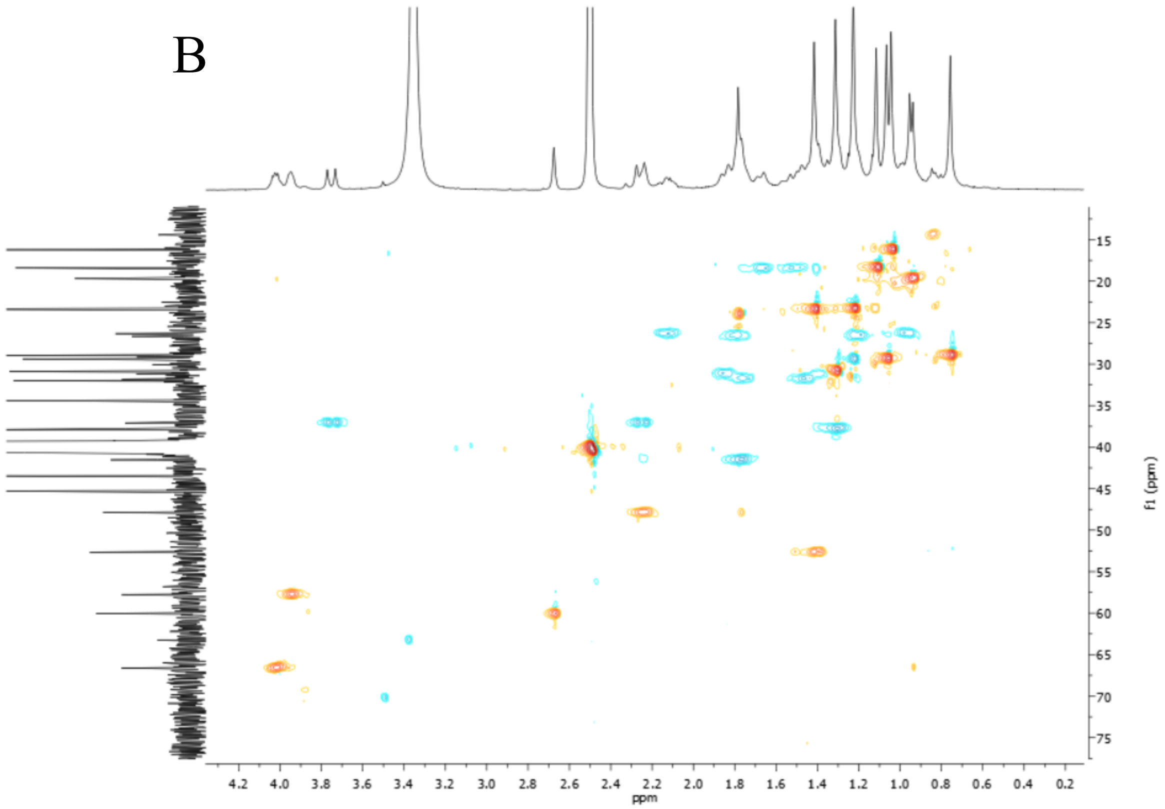

Figure S15. A) Full HSQC spectra of **5c** in DMSO-d$_6$. **B)** Highfield spectra.

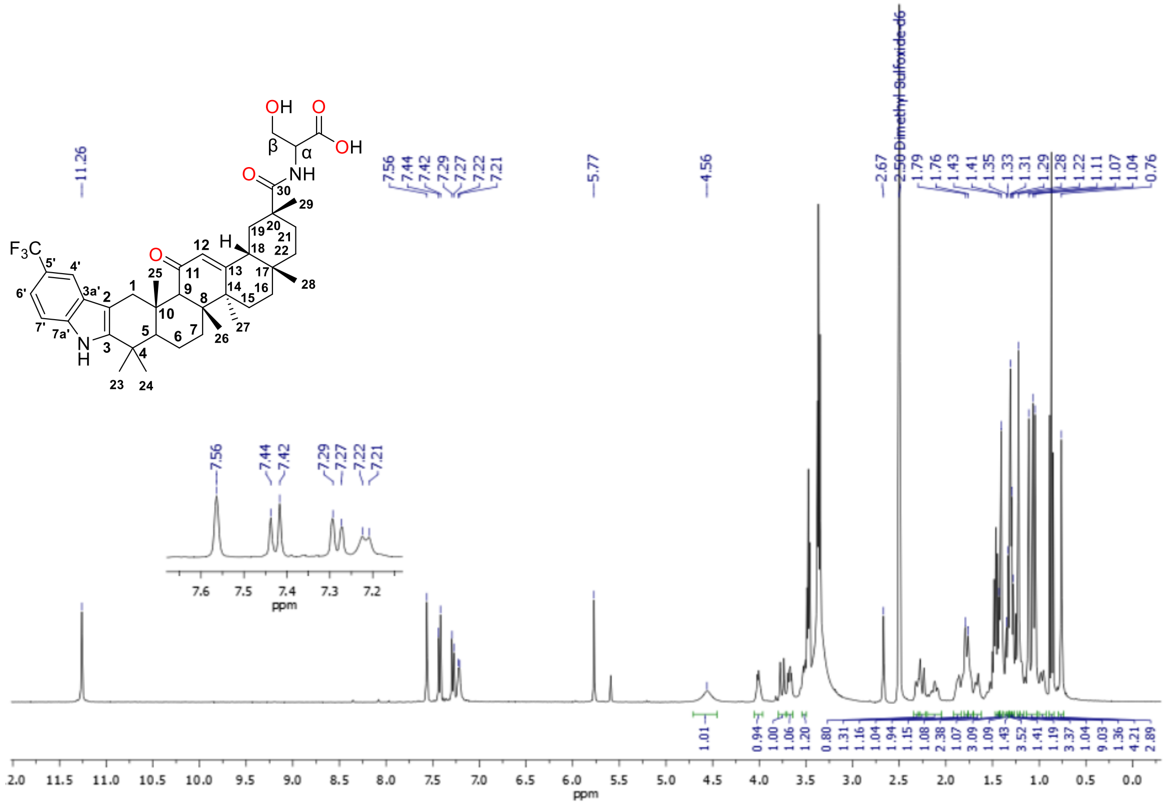

Figure S16. ^{1}H NMR (400 MHz) spectrum of **5d** in DMSO-d$_6$

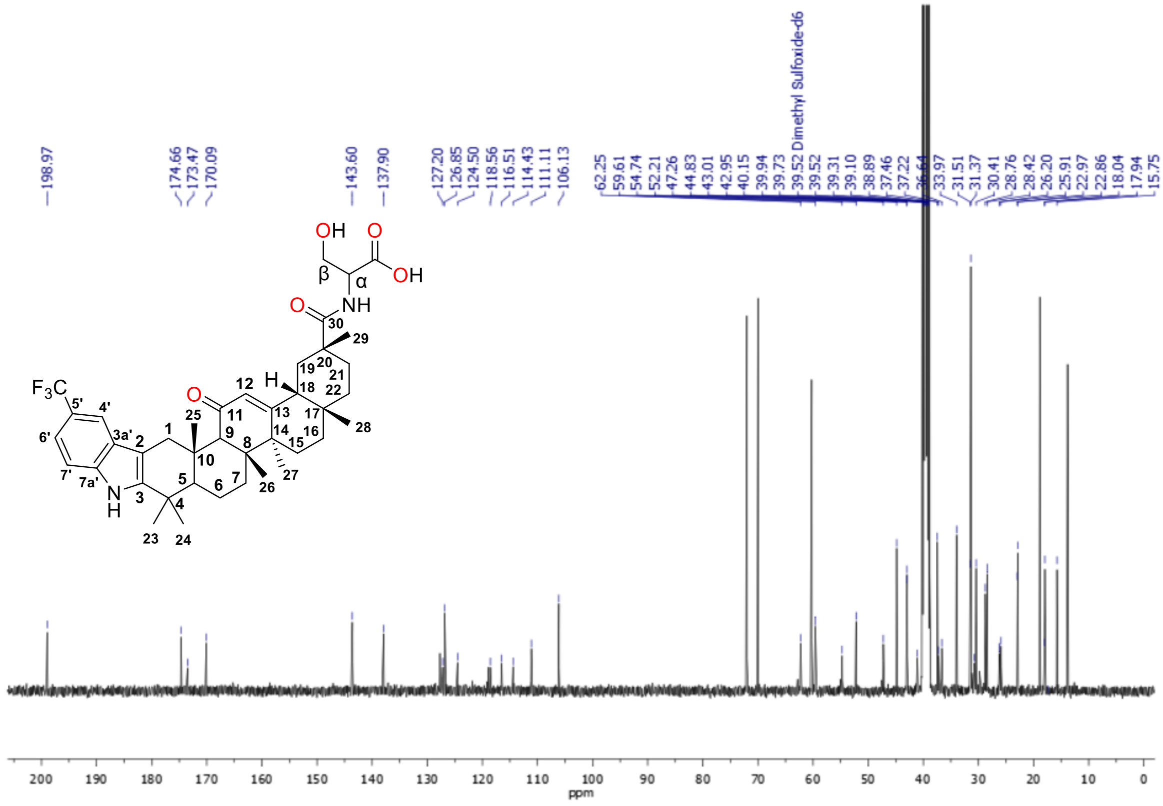

Figure S17. ^{13}C NMR (101 MHz) spectrum of **5d** in DMSO-d_6

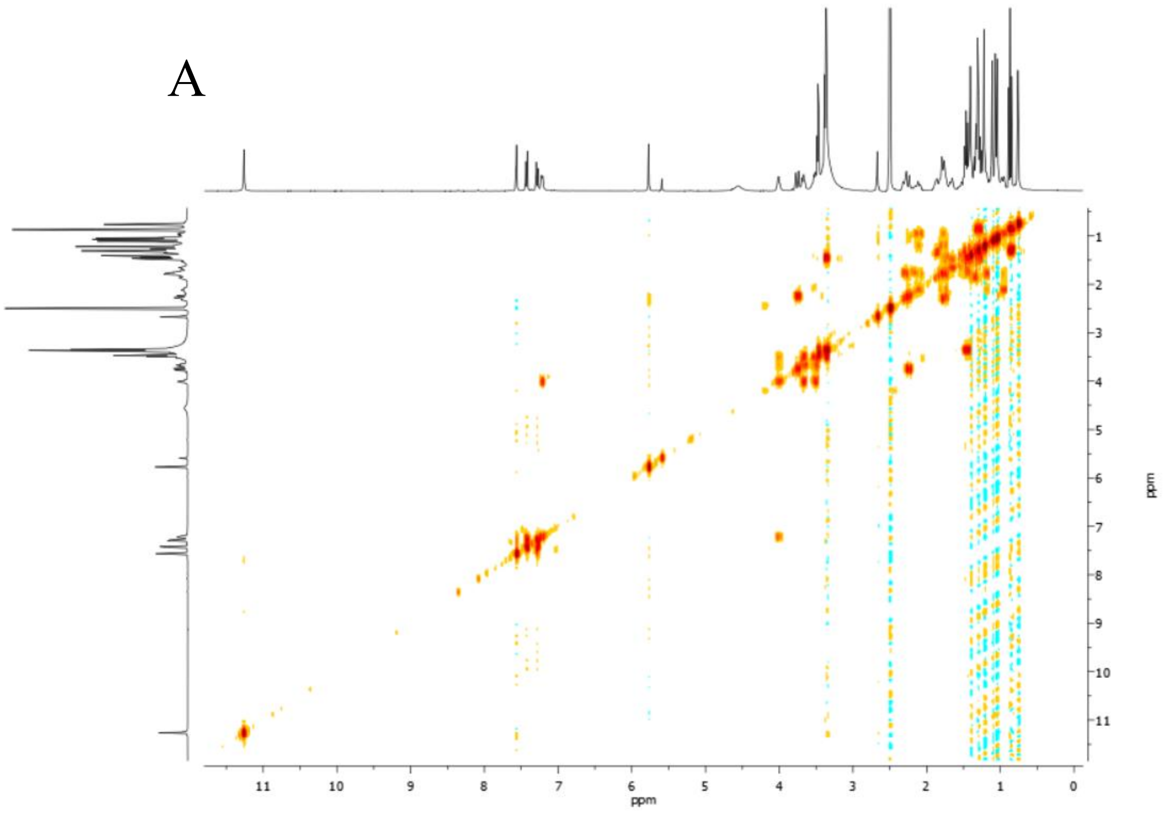

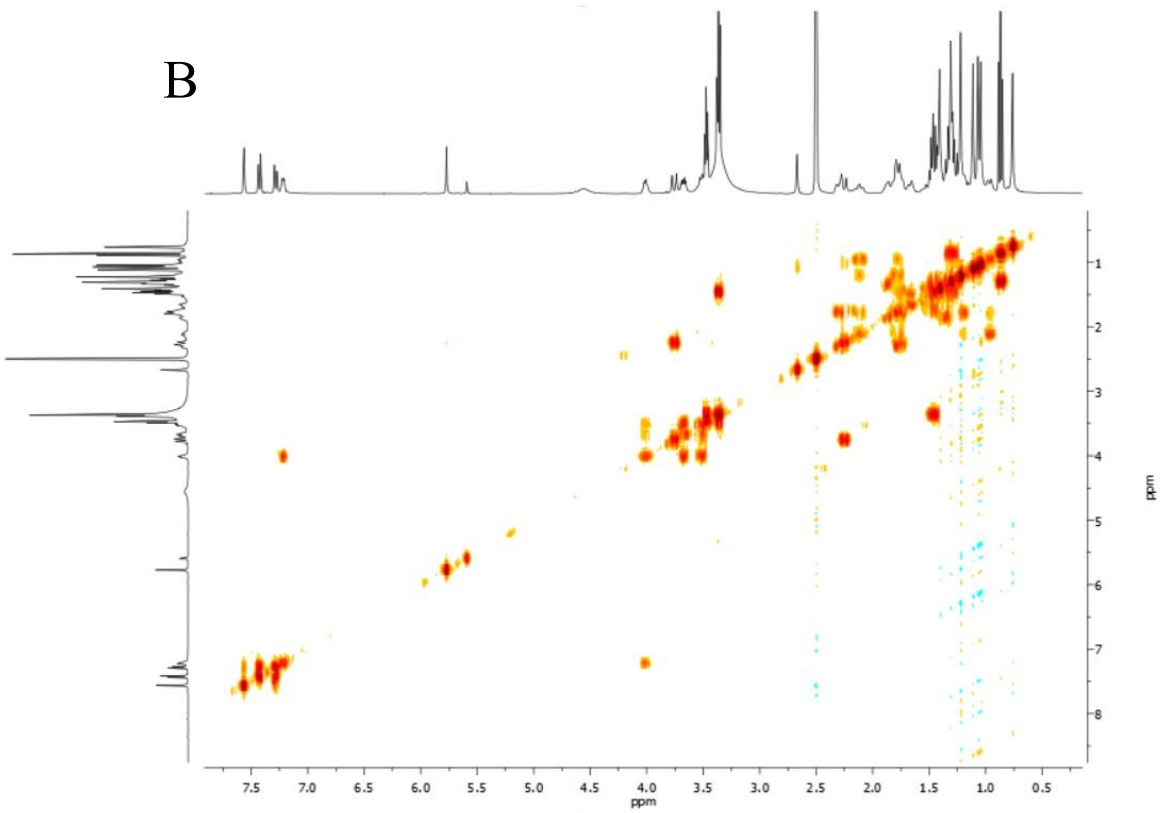

Figure S18. A) Full COSY spectra of **5d** in DMSO-d_6. **B)** Highfield spectra.

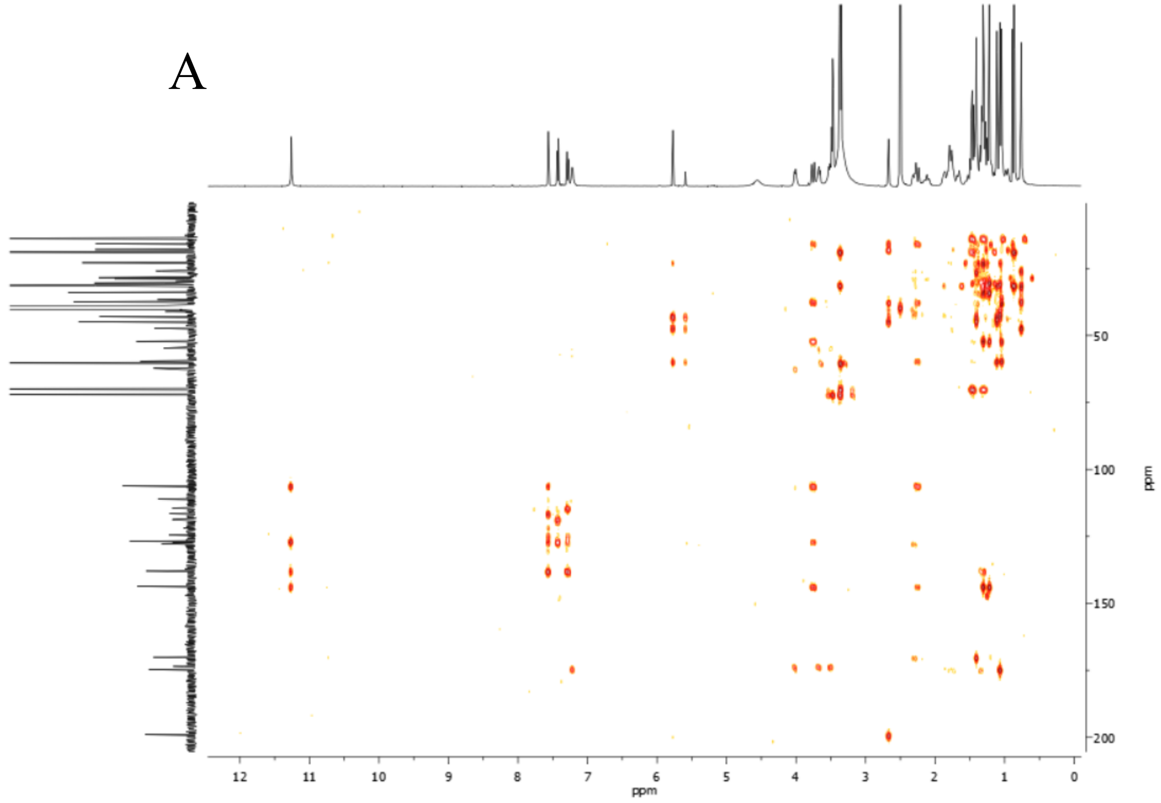

B

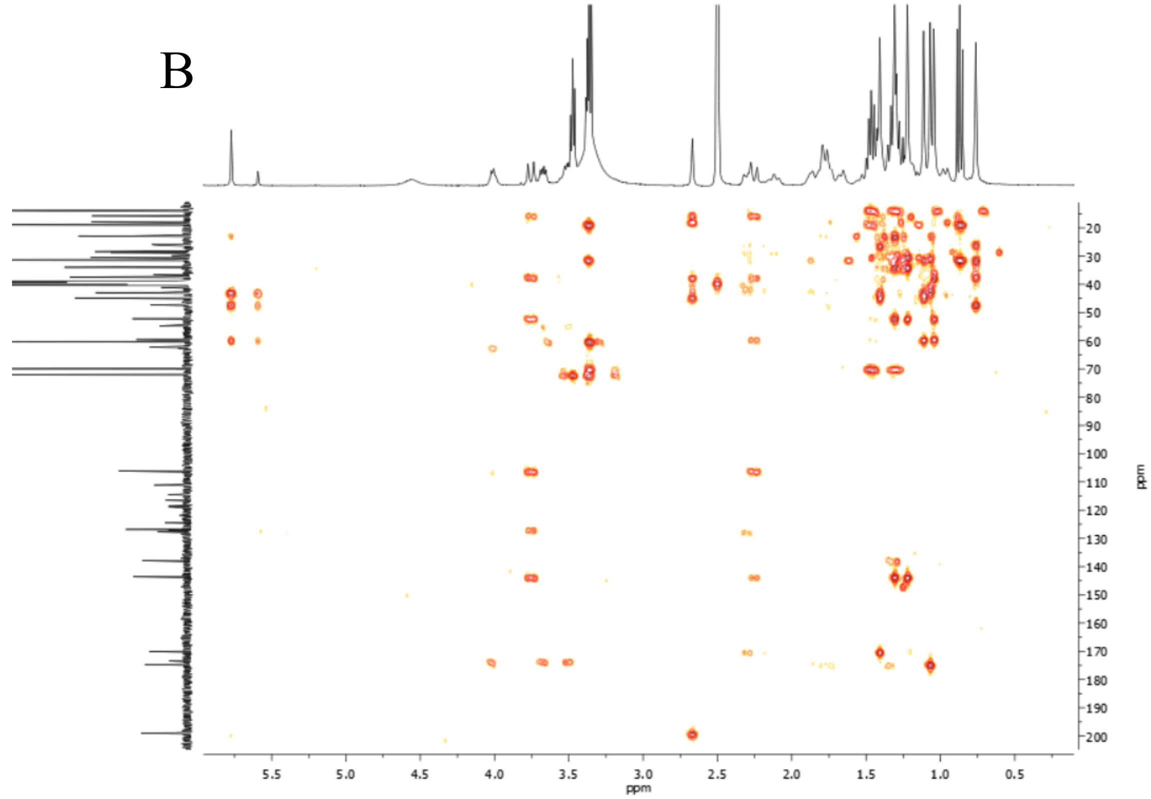

C

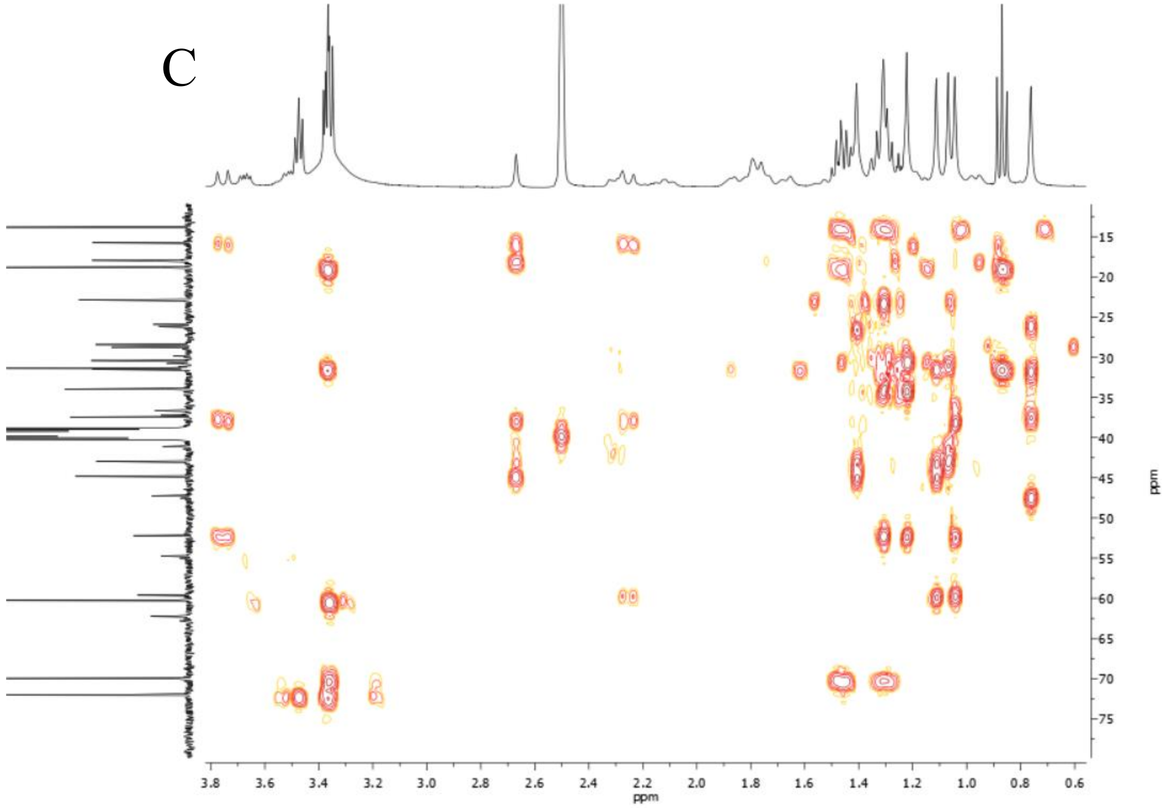

Figure S19. A) Full HMBC spectra of **5d** in DMSO-d_6. **B)** low-field spectra and **C).** Highfield spectra.

A

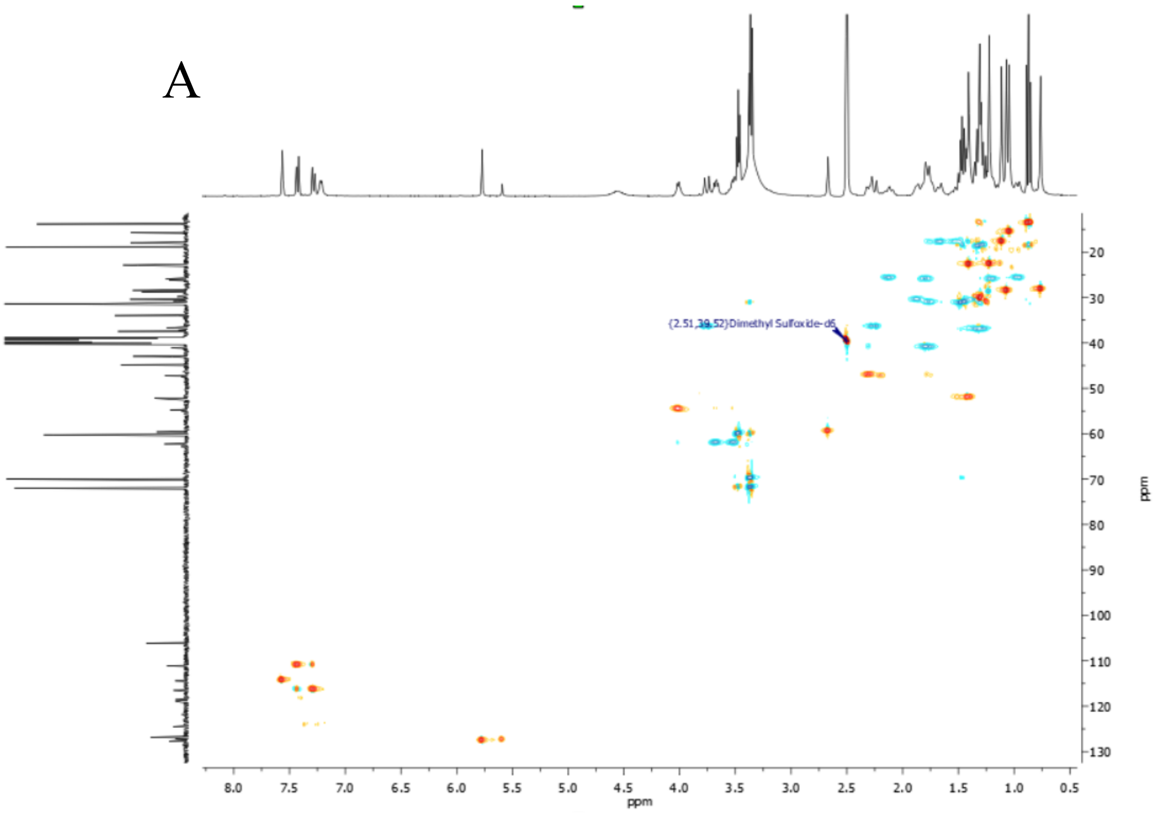

B

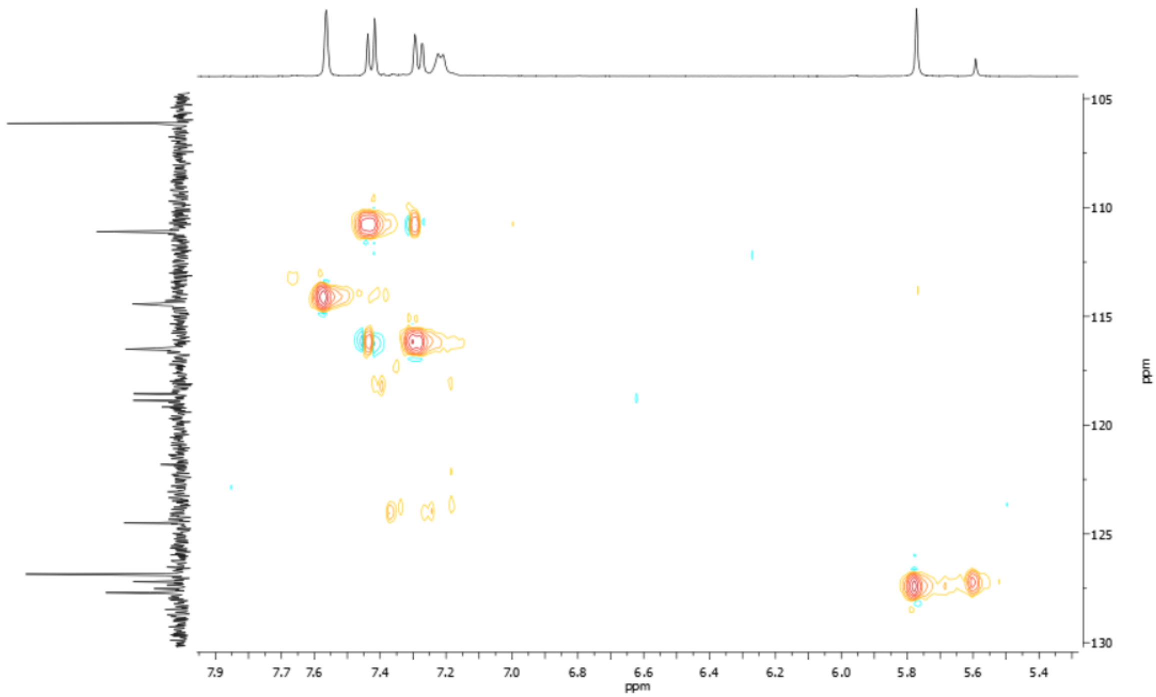

C

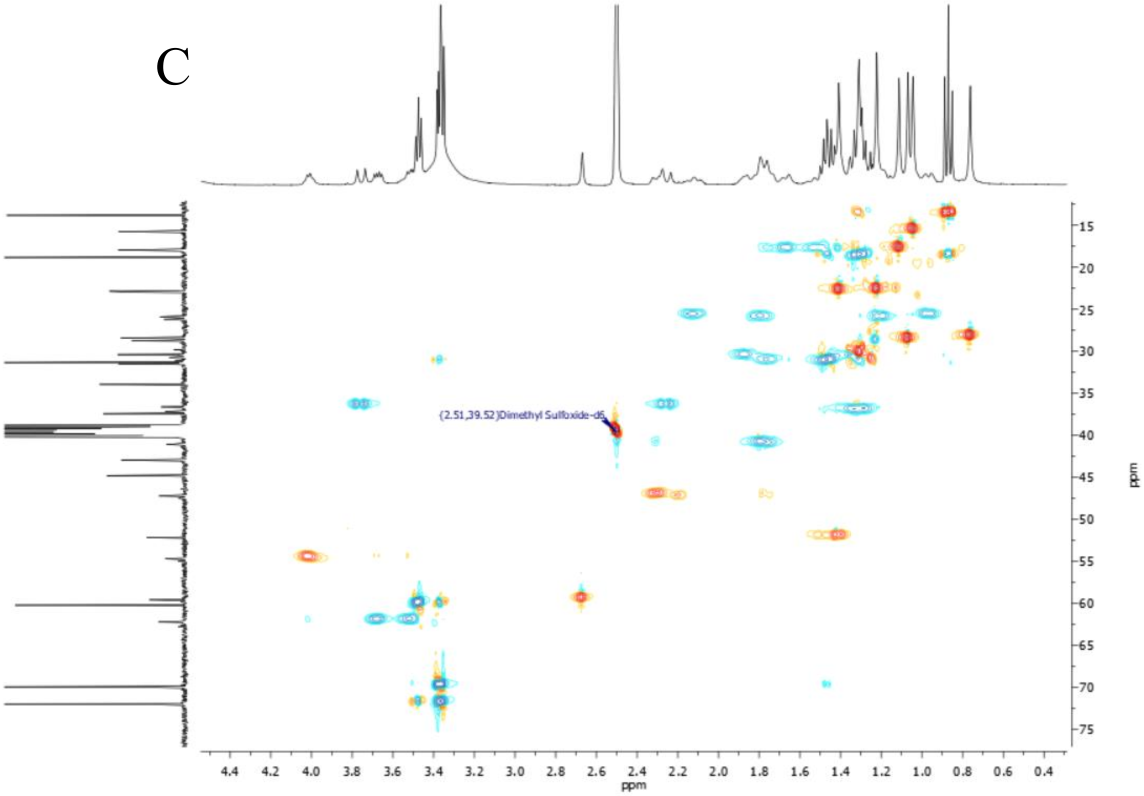

Figure S20. A) Full HSQC spectra of **5d** in DMSO-d$_6$. **B)** low-field spectra and **C).** Highfield spectra.

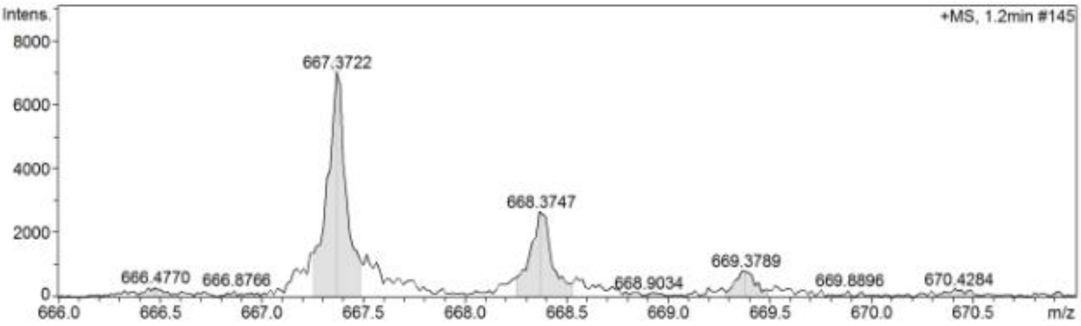

Mass Spectrum SmartFormula Report

Analysis Info

Analysis Name	D:\Data\Monica Rincon\Francisco Cortes Benitez\20240819_FC114-Glicina_.d	Acquisition Date	8/21/2024 3:31:04 AM
Method	Tune Low extendido.m	Operator	Admin
Sample Name	20240819_FC114-Glicina_	Instrument	micrOTOF 213750.00410
Comment			

Acquisition Parameter

Source Type	ESI	Ion Polarity	Positive	Set Nebulizer	0.5 Bar
Focus	Active			Set Dry Heater	150 °C
Scan Begin	50 m/z	Set Capillary	4500 V	Set Dry Gas	4.0 l/min
Scan End	3000 m/z	Set End Plate Offset	-500 V	Set Divert Valve	Waste

Meas. m/z	#	Ion Formula	Sum Formula	m/z	err [ppm]	|err| [mDa]	z	mSigma	# mSigma	e⁻ Conf
667.3722	1	C39H50F3N2O4	C39H49F3N2O4	667.3717	-0.8	0.5	1+	30.7	1	even
689.3563	1	C39H49F3N2NaO4		689.3537	-3.9	2.7	1+	31.5	1	even

Figure S21. Mass spectrum formula report of compound **5a**

Mass Spectrum SmartFormula Report

Analysis Info

Analysis Name	D:\Data\Monica Rincon\Francisco Cortes Benitez\20251113_FC114-ARG.d	Acquisition Date	11/14/2025 5:33:50 AM
Method	Tune Low extendido.m	Operator	Admin
Sample Name	20251113_FC114-ARG	Instrument	micrOTOF 213750.00410
Comment			

Acquisition Parameter

Source Type	ESI	Ion Polarity	Positive	Set Nebulizer	0.5 Bar
Focus	Active			Set Dry Heater	150 °C
Scan Begin	50 m/z	Set Capillary	4500 V	Set Dry Gas	4.0 l/min
Scan End	3000 m/z	Set End Plate Offset	-500 V	Set Divert Valve	Waste

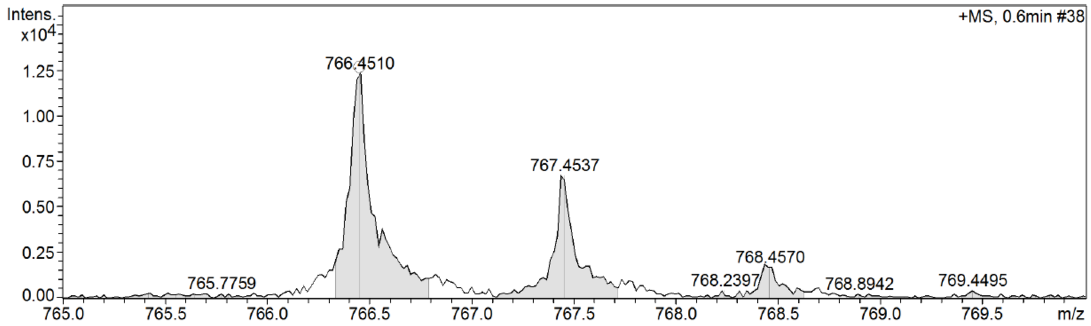

Meas. m/z	#	Ion Formula	Sum Formula	m/z	err [ppm]	|err| [mDa]	mSigma	# mSigma	elem. comp.
766.4510	1	C43H59F3N5O4	C43H58F3N5O4	766.4514	0.4	0.3	30.7	1	C 67.34 % H 7.75 % F 7.43 % N 9.13 % O 8.34 %

Figure S22. Mass spectrum formula report of compound **5b**

Mass Spectrum SmartFormula Report

Analysis Info

Analysis Name	D:\Data\Monica Rincon\Francisco Cortes Benitez\20251113_FC114-THR.d	Acquisition Date	11/14/2025 5:12:43 AM
Method	Tune Low extendido.m	Operator	Admin
Sample Name	20251113_FC114-THR	Instrument	micrOTOF 213750.00410
Comment			

Acquisition Parameter

Source Type	ESI	Ion Polarity	Positive	Set Nebulizer	0.5 Bar
Focus	Active			Set Dry Heater	150 °C
Scan Begin	50 m/z	Set Capillary	4500 V	Set Dry Gas	4.0 l/min
Scan End	3000 m/z	Set End Plate Offset	-500 V	Set Divert Valve	Waste

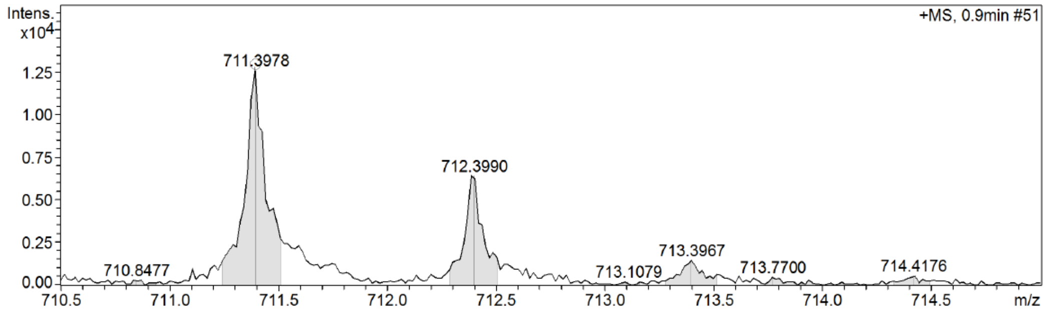

Meas. m/z	#	Ion Formula	Sum Formula	m/z	err [ppm]	|err| [mDa]	mSigma	# mSigma	elem. comp.
711.3978	1	C41H54F3N2O5	C41H53F3N2O5	711.3979	0.2	0.1	27.1	2	C 69.18 % H 7.65 % F 8.01 % N 3.94 % O 11.24 %
733.3806	1	C41H53F3N2NaO5		733.3799	-0.9	0.7	18.5	1	C 67.10 % H 7.28 % F 7.77 % N 3.82 % Na 3.13 % O 10.90 %

Figure S23. Mass spectrum formula report of compound **5c**

Mass Spectrum SmartFormula Report

Analysis Info

Analysis Name	D:\Data\Monica Rincon\Francisco Cortes Benitez\20240819_FC114-Serina.d	Acquisition Date	8/21/2024 2:55:12 AM
Method	Tune Low extendido.m	Operator	Admin
Sample Name	20240819_FC114-Serina	Instrument	micrOTOF 213750.00410
Comment			

Acquisition Parameter

Source Type	ESI	Ion Polarity	Positive	Set Nebulizer	0.5 Bar
Focus	Active			Set Dry Heater	150 °C
Scan Begin	50 m/z	Set Capillary	4500 V	Set Dry Gas	4.0 l/min
Scan End	3000 m/z	Set End Plate Offset	-500 V	Set Divert Valve	Waste

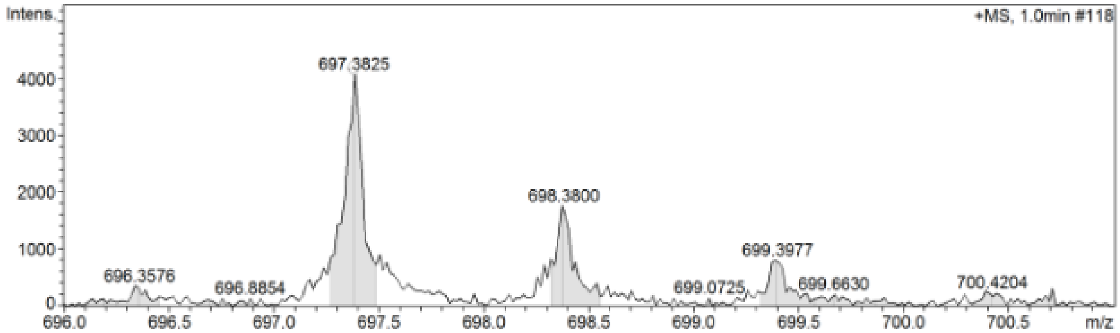

Meas. m/z	#	Ion Formula	Sum Formula	m/z	err [ppm]	|err| [mDa]	z	mSigma	# mSigma	e⁻ Conf
697.3825	1	C40H52F3N2O5	C40H51F3N2O5	697.3823	-0.2	0.2	1+	46.9	1	even
719.3636	1	C40H51F3N2NaO5		719.3642	0.9	0.6	1+	71.5	1	even

Figure S24. Mass spectrum formula report of compound **5d**

Signal DAD1 A, Sig=230,4 Ref=360,100 (DRCORTES\DERIVADOSAMINO 2005-01-19 23-24-24\FC-114-GLYC-4.D

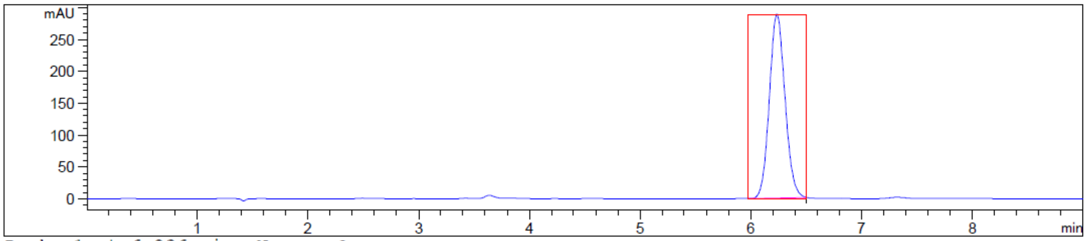

Peak :1 at 6.236 min Name : ?

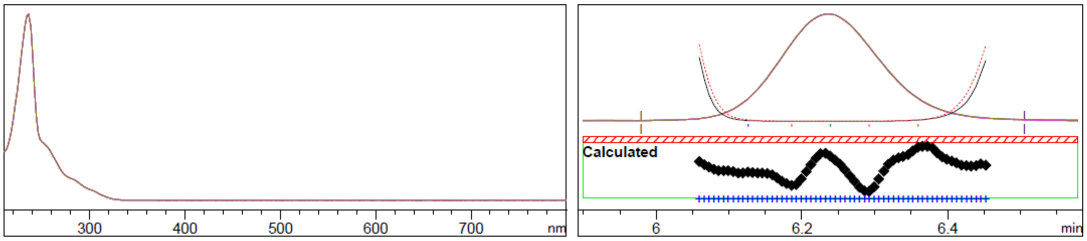

-> The purity factor is within the calculated threshold limit. <-

```
Purity factor : 999.883 (60 of 60 spectra are within the calculated threshold limit.)
Threshold     : 999.822  (Calculated with 60 of 60 spectra)
Reference     : Peak start and end spectra (integrated) (5.979 / 6.506)
Spectra       : 5  (Selection automatic, 5)
Noise Threshol: 0.041  (12 spectra, St.Dev 0.0202 + 3 * 0.007)
```

Figure S25. Chromatogram of compound **5a**

Signal DAD1 A, Sig=230,4 Ref=360,100 (DRCORTES\FC-114-ARGM7.D)

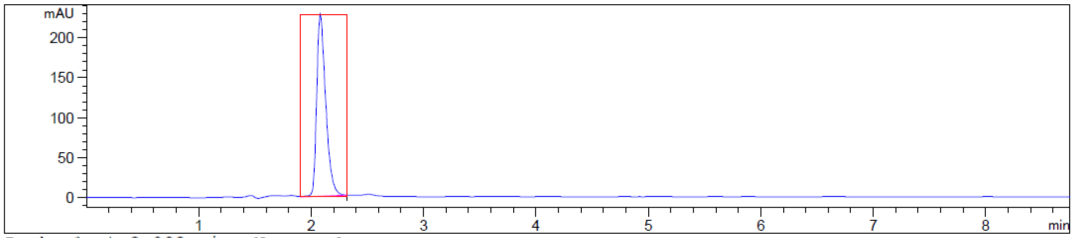

Peak :1 at 2.082 min Name : ?

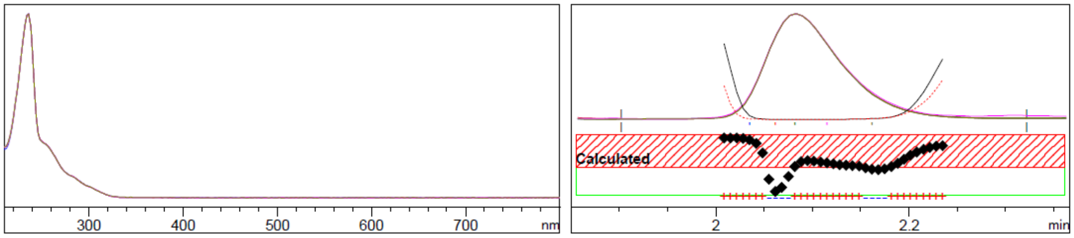

-> The purity factor exceeds the calculated threshold limit. <-

```
Purity factor : 998.753 (27 of 35 spectra exceed the calculated threshold limit.)
Threshold     : 999.694  (Calculated with 27 of 35 spectra)
Reference     : Peak start and end spectra (integrated) (1.902 / 2.322)
Spectra       : 5  (Selection automatic, 5)
Noise Threshol: 0.042  (12 spectra, St.Dev 0.0202 + 3 * 0.0071)
```

Figure S26. Chromatogram of compound **5b**

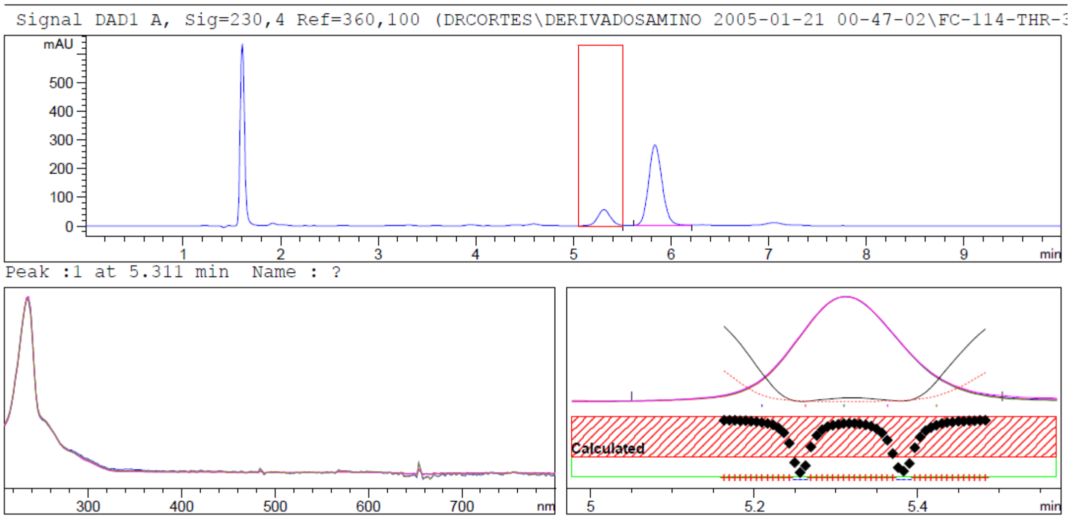

-> The purity factor exceeds the calculated threshold limit. <-

```
Purity factor : 993.846 (43 of 49 spectra exceed the calculated threshold limit.)
Threshold     : 999.532  (Calculated with 43 of 49 spectra)
Reference     : No Reference
Spectra       : 5  (Selection automatic, 5)
Noise Threshol: 0.040  (12 spectra, St.Dev 0.0195 + 3 * 0.0068)
```

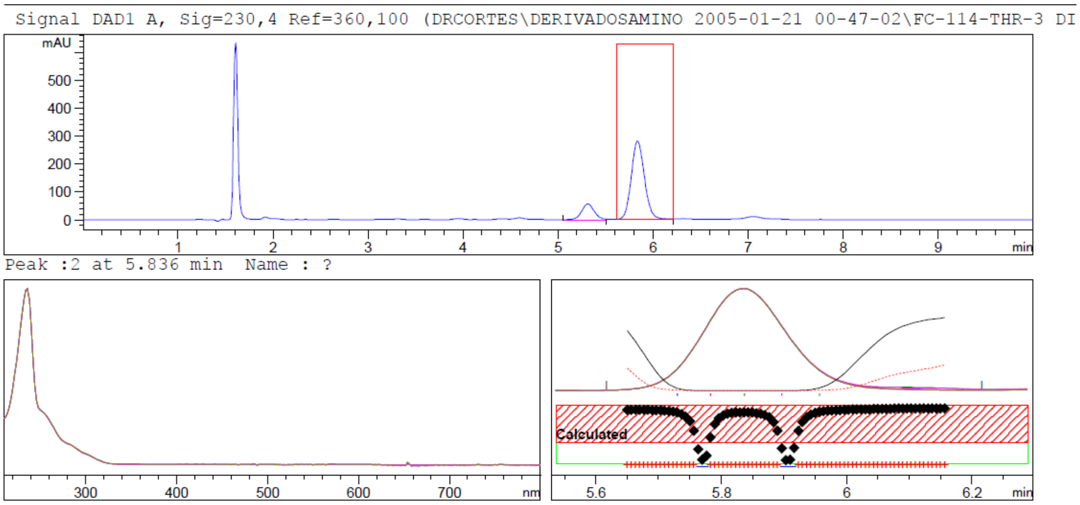

-> The purity factor exceeds the calculated threshold limit. <-

```
Purity factor : 985.492 (70 of 77 spectra exceed the calculated threshold limit.)
Threshold     : 999.533  (Calculated with 70 of 77 spectra)
Reference     : No Reference
Spectra       : 5  (Selection automatic, 5)
Noise Threshol: 0.040  (12 spectra, St.Dev 0.0195 + 3 * 0.0068)
```

Figure S27. Chromatogram of compound **5c**

Signal DAD1 A, Sig=230,4 Ref=360,100 (DRCORTES\DERIVADOSAMINO 2005-01-20 02-43-12\FC-114-SER DIL.

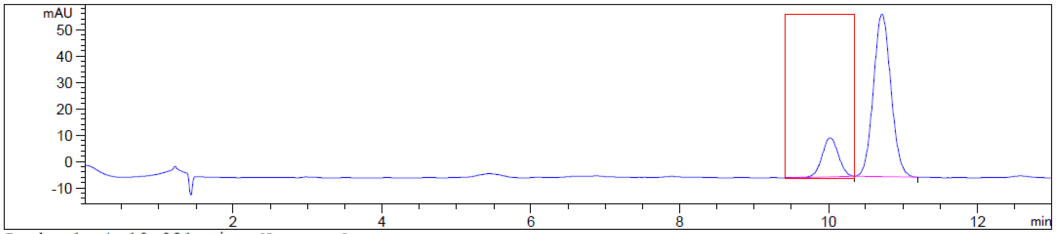

Peak :1 at 10.021 min Name : ?

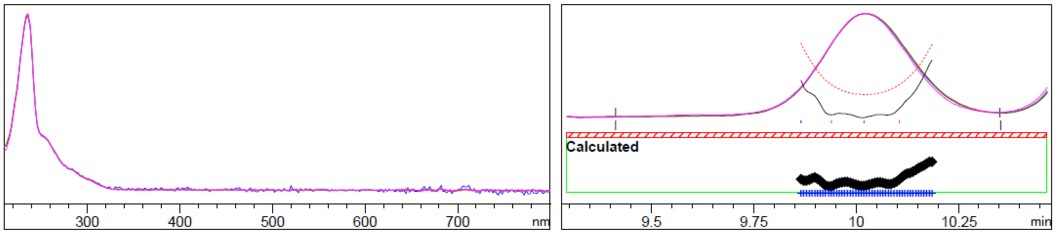

-> The purity factor is within the calculated threshold limit. <-

```
Purity factor : 999.404 (49 of 49 spectra are within the calculated threshold limit.)
Threshold     : 998.023  (Calculated with 49 of 49 spectra)
Reference     : Peak start and end spectra (integrated) (9.412 / 10.352)
Spectra       : 4  (Selection automatic, 5)
Noise Threshol: 0.066  (12 spectra, St.Dev 0.0303 + 3 * 0.0118)
```

Signal DAD1 A, Sig=230,4 Ref=360,100 (DRCORTES\DERIVADOSAMINO 2005-01-20 02-43-12\FC-114-SER DIL.

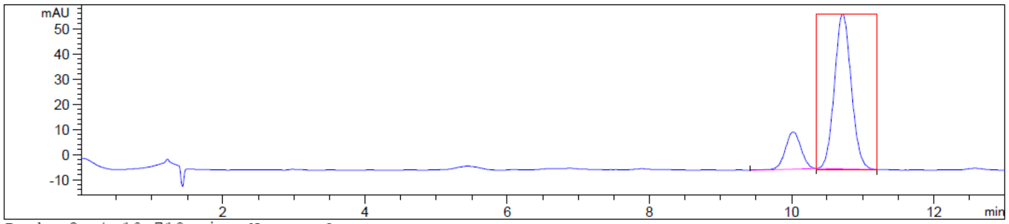

Peak :2 at 10.718 min Name : ?

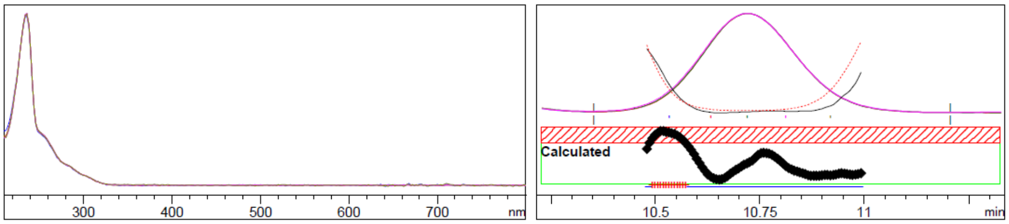

-> The purity factor exceeds the calculated threshold limit. <-

```
Purity factor : 998.644 (13 of 78 spectra exceed the calculated threshold limit.)
Threshold     : 998.927  (Calculated with 13 of 78 spectra)
Reference     : Peak start and end spectra (integrated) (10.352 / 11.206)
Spectra       : 5  (Selection automatic, 5)
Noise Threshol: 0.066  (12 spectra, St.Dev 0.0303 + 3 * 0.0118)
```

Figure S28. Chromatogram of compound **5d**

Inhibitory activity against PTP1B and TCPTP

The methodology for obtaining the recombinant proteins *h*PTP1B$_{1-400}$, *h*PTP1B$_{1-285}$ and *h*TCPTP$_{1-415}$, as well as the determination of IC$_{50}$ values and enzymatic kinetics for GA derivatives and positive controls, was conducted following the procedures established in previous studies performed by our group.[1–3]

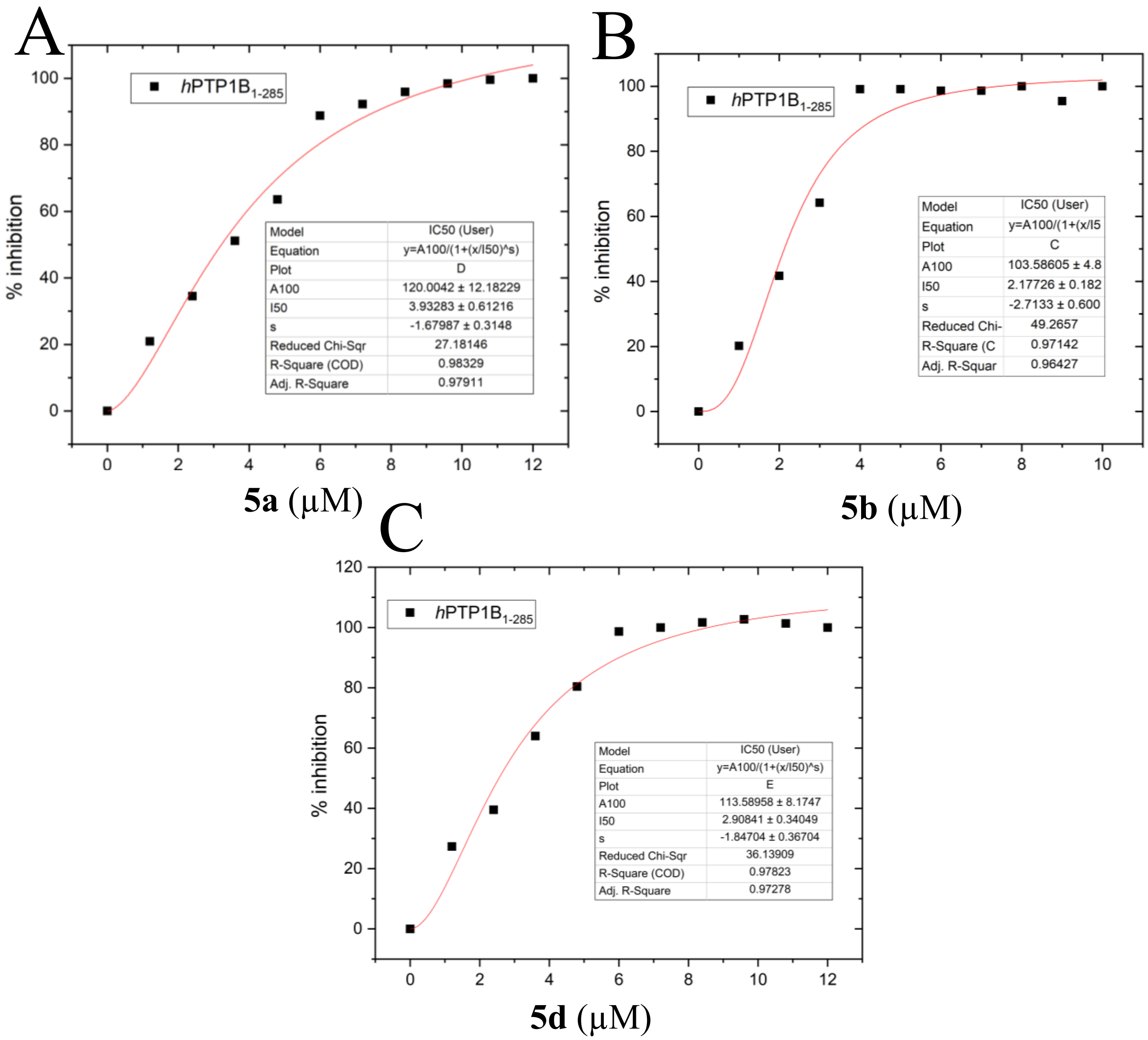

Figure S29. Inhibition of PTP1B$_{1-285}$ for amide derivatives. (**A**) compound **5a**; (**B**) compound **5b**; (**C**) compound **5d**

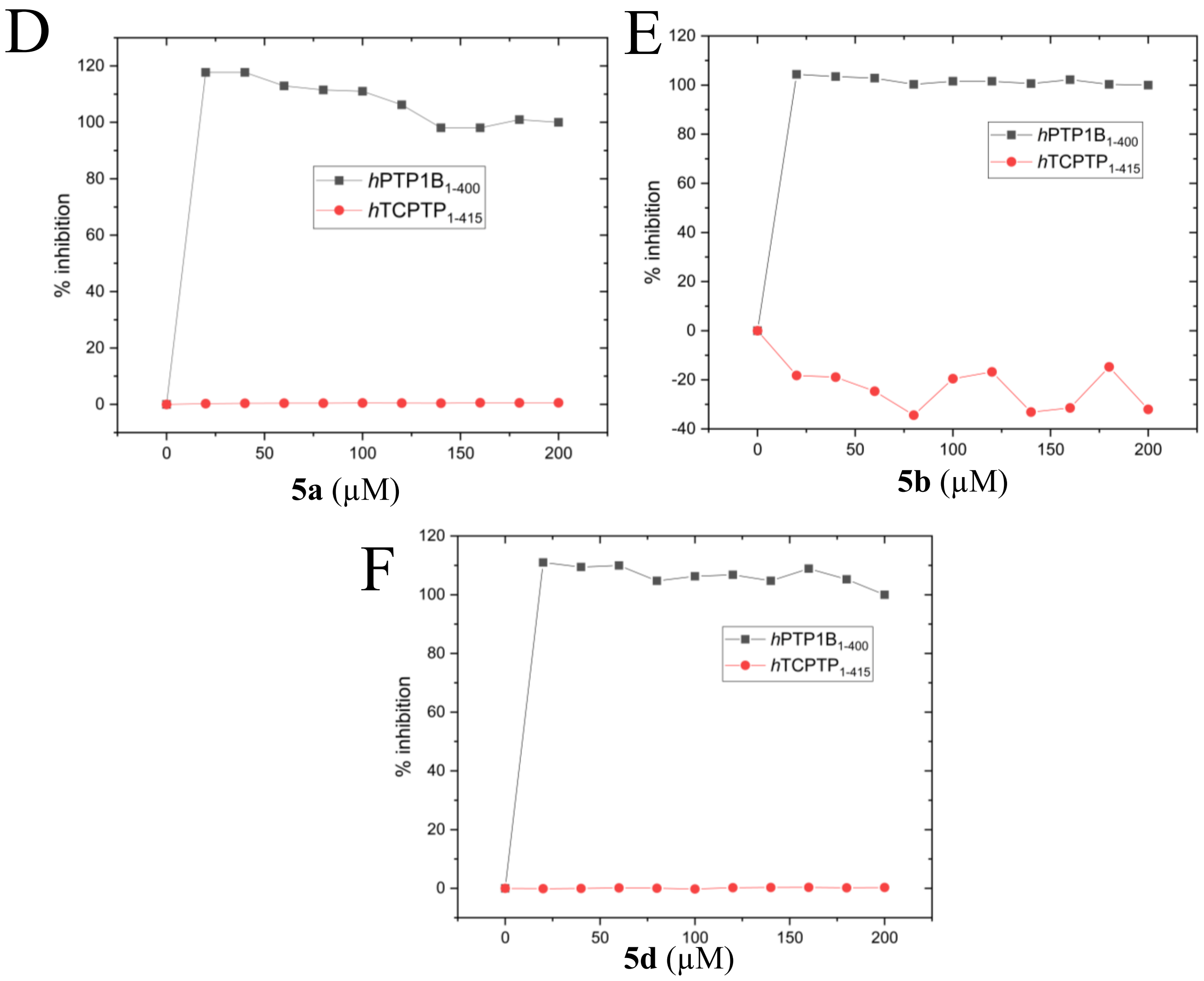

Figure S30. Selectivity of the inhibition for PTP1B over TCPTP for amide derivatives at 200 μM. (**D**) compound **5a**; (**E**) compound **5b**; (**F**) compound **5d**

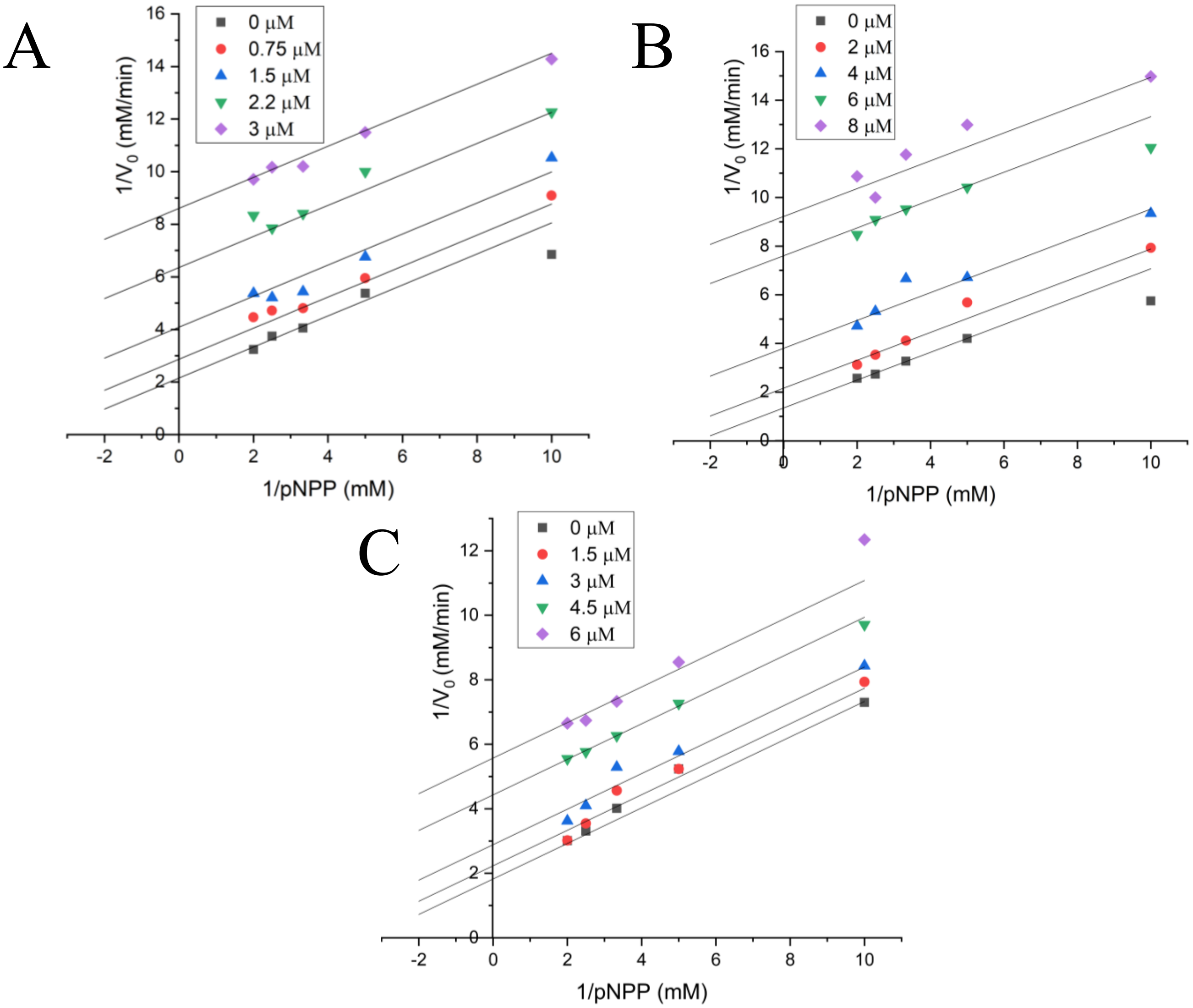

Figure S31. Lineweaver–Burk plots for *h*PTP1B$_{1\text{-}400}$ inhibition by compound **5a** (**A**), compound **5b** (**B**), and compound **5d** (**C**).

Molecular modelling of compounds 4, 5a-5d

The compounds were constructed in AVOGADRO[4] (version 1.2.0) (http://avogadro.cc/) by systematically modifying the structure of GA (with deposition number 1169430[5]), which was retrieved from the Cambridge Crystallographic Data Base (https://www.ccdc.cam.ac.uk/). Subsequently, the protonation states of all compounds were fixed at pH 7.4, and the 3D geometries were optimized using the Universal Force Field (UFF) in AVOGADRO and then saved as *.sdf files.

Molecular Docking

Molecular docking was performed using AutoDock and AutoDock Vina, both integrated into the YASARA Structure suite (version 24.10.5). Additionally, GOLD software (version 2024.3.0) was utilized in this study. The molecular docking simulations were performed using the PTP1B$_{1\text{-}400}$-pNPP model, which was previously reported by our group.[6,7]

For the blind docking simulations, a simulation cell or grid box encompassing the entire PTP1B$_{1\text{-}400}$-pNPP protein, with an additional 5 Å extension, was employed. The protein and simulation cell were saved as a *.sce file. Subsequently, molecular docking was performed using AutoDock and Vina with the protein (*.sce file) and GA ligands (*.sdf file) via the dock_runscreening macro, which was modified to include 200 runs of the Lamarckian Genetic Algorithm. The resulting binding poses indicated that the compounds primarily bind to two specific sites. Consequently, a site-specific molecular docking was conducted at these identified sites using a simulation cell (grid box) with an extension of 5 Å from the docked ligand. The search was conducted using the *.sdf ligand files and the *dock_run* macro, which had been previously modified to perform 100 Lamarckian Genetic Algorithm runs in Autodock. For Autodock Vina, 200 runs were performed. Finally, each ligand with the best cluster size and the lowest binding energy was selected for further analysis.

The PTP1B$_{1\text{-}400}$-pNPP-ligand complexes generated in YASARA structure were then exported to GOLD software. Using the GOLD wizard, the proteins were prepared by adding hydrogens and extracting ligands, which were then further docked at sites 1 or 2 within a 6 Å radius sphere, with the following parameters: 100 genetic algorithm runs and 125,000 operations. CHEMPLP fitness was chosen as the main scoring function, whereas GoldScore fitness was selected as the re-scoring function. The dockings were ranked according to the value of the CHEMPLP and GoldScore fitness function.

The binding poses shown in the figures were derived from Autodock results. However, the binding poses obtained from Vina and GOLD were similar.

Table S1. Results of molecular docking simulations against the PTP1B$_{1\text{-}400}$-pNPP.

	Site 1				Site 2			
	VINA	AD4.2	GOLD		VINA	AD4.2	GOLD	
Compound	Kcal/mol		ChemPLP	Goldscore	Kcal/mol		ChemPLP	Goldscore
**4**	-10.0	-10.0	38.6	28.6	-8.9	-10.7	51.5	37.9
**5a**	-9.7	-11.6	69.8	48.3	-10.0	-12.5	64.7	45.9
**5b**	-9.7	-13.9	69.3	45.8	-9.0	-12.1	67.7	14.9
**5c**	-9.7	-11.5	70.0	40.8	-9.7	-10.7	66.6	38.4
**5d**	-9.7	-11.6	65.5	41.7	-8.8	-10.8	64.3	43.7

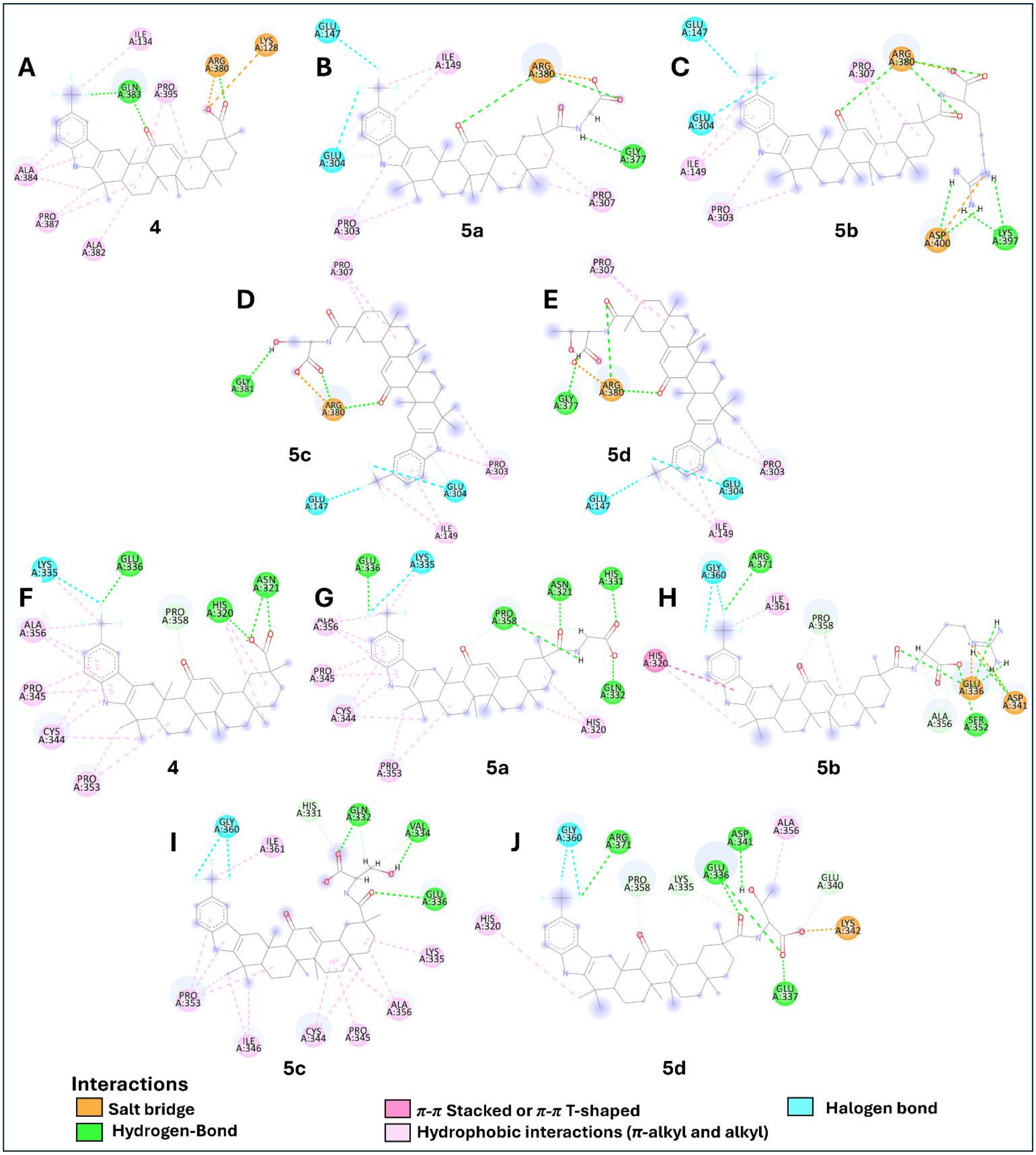

Figure S32. 2D-diagram for the interactions of compounds **4** and **5a–5d** within site 1 (A–E) and site 2 (F–J) of the PTP1B$_{1-400}$–pNPP complex.

Molecular Dynamics Simulations

The protein-ligand complexes were submitted to MDS with YASARA Structure[8,9] version 24.10.5. The simulations started with an optimization of the hydrogen bonding network to increase the solute stability and a pKa prediction to fine-tune the protonation states of protein residues at the chosen pH of 7.4, then NaCl ions were added with a physiological concentration of 0.9%, with an excess of either Na^+ or Cl^- to neutralize the cell. After the steepest descent and simulated annealing minimizations to remove clashes, the simulation was run for 300 nanoseconds using the AMBER11[10] force field for the solute, GAFF2[11] and AM1BCC[12] for ligands and TIP3P[13] for water. The cutoff was 8 Å for van der Waals forces (the default used by AMBER); no cutoff was applied to electrostatic forces (using the Particle Mesh Ewald algorithm[14]). The equations of motion were integrated using the YASARA default parameters: a multiple-timestep of 1.25 fs for bonded interactions and 2.5 fs for non-bonded interactions at a temperature of 310 K and a pressure of 1 atm (NPT ensemble), employing algorithms described in detail previously.[15] After inspecting the solute RMSD as a function of simulation time, the first 100 picoseconds were considered equilibration time and excluded from further analysis. The binding energy study using the MM/PBSA method was performed by running *the md_analyzebindingenergy* macro, which was previously modified with PBS method at a temperature of 310 K. The RMSD, RMSF, and binding energy plots were generated using the codes available at https://github.com/Franciscoqfb87/MDS-graphs.git

Table S2. Average RMSD, RMSF, and Binding Energy values for 300 ns of MD simulations on the PTP1B$_{1-400}$-pNPP and PTP1B$_{1-400}$-pNPP-ligand systems.

		PTP1B$_{1-400}$-pNPP	PTP1B$_{1-400}$-pNPP-**5a**	PTP1B$_{1-400}$-pNPP-**5b**	PTP1B$_{1-400}$-pNPP -**5c**	PTP1B$_{1-400}$-pNPP-**5d**
**Site 1**	Average RMSD (Å)	3.45	3.15	4.43	4.22	3.66
	Average RMSF (Å)	2.19	1.90	2.30	2.52	2.30
	Average Binding Energy (kcal/mol)	-	-52.61	-37.48	-33.76	-25.56
**Site 2**	Average RMSD (Å)	3.45	4.29	2.96	4.33	3.79
	Average RMSF (Å)	2.19	2.22	2.11	2.26	2.37
	Average Binding Energy (kcal/mol)	-	-103.16	-32.59	-120.56	-104.13

Table S3. Results of compounds **4** and **5a-5d** the physicochemical properties predicted SwissADME web tool

Compound		4	5a	5b	5c	5d
**Physicochemical**	Water solubility Log *S* (ESOL)	Poorly soluble -9.48	Poorly soluble -9.21	Poorly soluble -9.22	Poorly soluble -9.27	Poorly soluble -8.92
	Fraction Csp3	0.68	0.67	0.65	0.68	0.68
	Rotatable bonds	2	5	10	6	6
	Molar refractivity	167.63	180.34	208.67	191.12	186.31
	TPSA (Å²)	70.16	99.26	161.16	119.49	119.49
**Drug linkeness Lipinski**	H-bond donors	2	3	6	4	4
	MW g/mol	609.8	666.8	765.9	710.9	696.84
	Consensus Log $P_{o/w}$	7.86	7.12	6.83	7.06	6.92
	H-bond acceptors	6	7	8	8	8
	# violations	2	2	3	2	2
**Pharmacokinetic**	GI absorption	Low	Low	Low	Low	Low
	BBB permeant	No	No	No	No	No
	P-gp substrate	No	No	Yes	No	No
	Inhibitor	No	CYP3A4	CYP3A4	CYP3A4	CYP3A4
**Medicinal Chemistry**	Bioavailability Score	0.85	0.56	0.17	0.56	0.56
	PAINS alerts	0	0	0	0	0
	Brenk alerts	0	0	2	0	0
	Leadlikeness violations	MW>350 XLOGP3>3.5	MW>350 XLOGP3>3.5	MW>350 Rotors>7 XLOGP3>3.5	MW>350 XLOGP3>3.5	MW>350 XLOGP3>3.5
	Synthetic Accessibility	6.15	6.32	7.13	6.77	6.59

Molecule 1

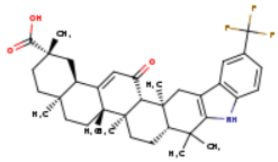

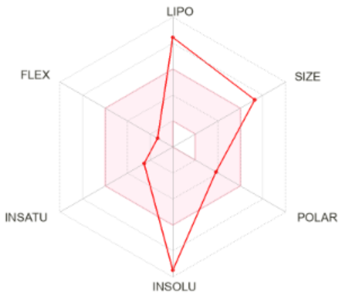

SMILES	O=C1C=C2[C@@H]3C[C@](C)(CC[C@]3(C)CC[C@]2([C@]2([C@H]1[C@@]1(C)Cc3c4cc(ccc4[nH]c3C([C@@H]1CC2)(C)C)C(F)(F)F)C)C)C(=O)O

Physicochemical Properties	
Formula	C37H46F3NO3
Molecular weight	609.76 g/mol
Num. heavy atoms	44
Num. arom. heavy atoms	9
Fraction Csp3	0.68
Num. rotatable bonds	2
Num. H-bond acceptors	6
Num. H-bond donors	2
Molar Refractivity	167.73
TPSA	70.16 Å²

Lipophilicity	
Log $P_{o/w}$ (iLOGP)	4.14
Log $P_{o/w}$ (XLOGP3)	9.27
Log $P_{o/w}$ (WLOGP)	10.42
Log $P_{o/w}$ (MLOGP)	6.54
Log $P_{o/w}$ (SILICOS-IT)	8.95
Consensus Log $P_{o/w}$	7.86

Water Solubility	
Log *S* (ESOL)	-9.48
Solubility	2.02e-07 mg/ml ; 3.31e-10 mol/l
Class	Poorly soluble
Log *S* (Ali)	-10.64
Solubility	1.38e-08 mg/ml ; 2.27e-11 mol/l
Class	Insoluble
Log *S* (SILICOS-IT)	-10.36
Solubility	2.64e-08 mg/ml ; 4.33e-11 mol/l
Class	Insoluble

Pharmacokinetics	
GI absorption	Low
BBB permeant	No
P-gp substrate	No
CYP1A2 inhibitor	No
CYP2C19 inhibitor	No
CYP2C9 inhibitor	No
CYP2D6 inhibitor	No
CYP3A4 inhibitor	No
Log K_p (skin permeation)	-3.44 cm/s

Druglikeness	
Lipinski	No; 2 violations: MW>500, MLOGP>4.15
Ghose	No; 4 violations: MW>480, WLOGP>5.6, MR>130, #atoms>70
Veber	Yes
Egan	No; 1 violation: WLOGP>5.88
Muegge	No; 2 violations: MW>600, XLOGP3>5
Bioavailability Score	0.85

Medicinal Chemistry	
PAINS	0 alert
Brenk	0 alert
Leadlikeness	No; 2 violations: MW>350, XLOGP3>3.5
Synthetic accessibility	6.15

Figure S33. Physicochemical and pharmacokinetic properties of compound **4** calculated using the free web tool SwissADME

Molecule 2

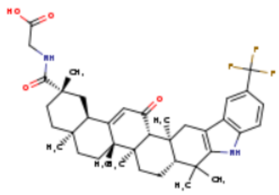

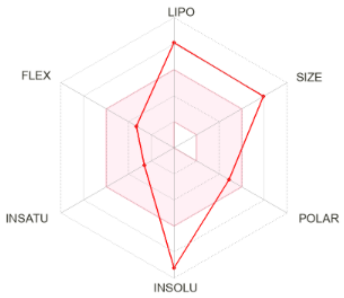

SMILES	OC(=O)CNC(=O)[C@@]1(C)CC[C@]2([C@@H](C1)C1=CC(=O)[C@H]3[C@@]([C@@]1(CC2)C)(C)CC[C@@H]1[C@]3(C)Cc2c3cc(ccc3[nH]c2C1(C)C)C(F)(F)F)C

Physicochemical Properties	
Formula	C39H49F3N2O4
Molecular weight	666.81 g/mol
Num. heavy atoms	48
Num. arom. heavy atoms	9
Fraction Csp3	0.67
Num. rotatable bonds	5
Num. H-bond acceptors	7
Num. H-bond donors	3
Molar Refractivity	180.34
TPSA	99.26 Å²

Lipophilicity	
Log $P_{o/w}$ (iLOGP)	3.31
Log $P_{o/w}$ (XLOGP3)	8.62
Log $P_{o/w}$ (WLOGP)	9.53
Log $P_{o/w}$ (MLOGP)	5.57
Log $P_{o/w}$ (SILICOS-IT)	8.56
Consensus Log $P_{o/w}$	7.12

Water Solubility	
Log *S* (ESOL)	-9.21
Solubility	4.08e-07 mg/ml ; 6.12e-10 mol/l
Class	Poorly soluble
Log *S* (Ali)	-10.58
Solubility	1.75e-08 mg/ml ; 2.63e-11 mol/l
Class	Insoluble
Log *S* (SILICOS-IT)	-10.69
Solubility	1.36e-08 mg/ml ; 2.05e-11 mol/l
Class	Insoluble

Pharmacokinetics	
GI absorption	Low
BBB permeant	No
P-gp substrate	No
CYP1A2 inhibitor	No
CYP2C19 inhibitor	No
CYP2C9 inhibitor	No
CYP2D6 inhibitor	No
CYP3A4 inhibitor	Yes
Log K_p (skin permeation)	-4.25 cm/s

Druglikeness	
Lipinski	No; 2 violations: MW>500, MLOGP>4.15
Ghose	No; 4 violations: MW>480, WLOGP>5.6, MR>130, #atoms>70
Veber	Yes
Egan	No; 1 violation: WLOGP>5.88
Muegge	No; 2 violations: MW>600, XLOGP3>5
Bioavailability Score	0.56

Medicinal Chemistry	
PAINS	0 alert
Brenk	0 alert
Leadlikeness	No; 2 violations: MW>350, XLOGP3>3.5
Synthetic accessibility	6.32

Figure S34. Physicochemical and pharmacokinetic properties of **5a** calculated using the free web tool SwissADME.

Molecule 3

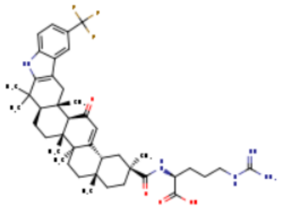

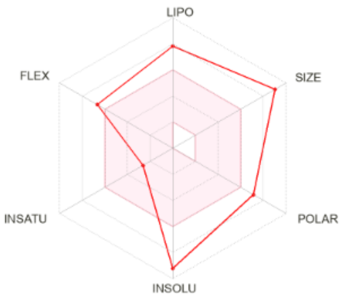

SMILES: NC(=N)NCCC[C@@H](C(=O)O)NC(=O)[C@@]1(C)CC[C@]2([C@@H](C1)C1=CC(=O)[C@H]3[C@@]([C@@]1(CC2)C)(C)CC[C@@H]1[C@]3(C)Cc2c3cc(ccc3[nH]c2C1(C)C)C(F)(F)F)C

Physicochemical Properties	
Formula	C43H58F3N5O4
Molecular weight	765.95 g/mol
Num. heavy atoms	55
Num. arom. heavy atoms	9
Fraction Csp3	0.67
Num. rotatable bonds	10
Num. H-bond acceptors	8
Num. H-bond donors	6
Molar Refractivity	208.67
TPSA	161.16 Å²

Lipophilicity	
Log $P_{o/w}$ (iLOGP)	3.61
Log $P_{o/w}$ (XLOGP3)	8.20
Log $P_{o/w}$ (WLOGP)	9.17
Log $P_{o/w}$ (MLOGP)	5.02
Log $P_{o/w}$ (SILICOS-IT)	8.15
Consensus Log $P_{o/w}$	6.83

Water Solubility	
Log *S* (ESOL)	-9.22
Solubility	4.66e-07 mg/ml ; 6.08e-10 mol/l
Class	Poorly soluble
Log *S* (Ali)	-11.44
Solubility	2.75e-09 mg/ml ; 3.59e-12 mol/l
Class	Insoluble
Log *S* (SILICOS-IT)	-11.18
Solubility	5.03e-09 mg/ml ; 6.56e-12 mol/l
Class	Insoluble

Pharmacokinetics	
GI absorption	Low
BBB permeant	No
P-gp substrate	Yes
CYP1A2 inhibitor	No
CYP2C19 inhibitor	No
CYP2C9 inhibitor	No
CYP2D6 inhibitor	No
CYP3A4 inhibitor	Yes
Log K_p (skin permeation)	-5.15 cm/s

Druglikeness	
Lipinski	No; 3 violations: MW>500, MLOGP>4.15, NHorOH>5
Ghose	No; 4 violations: MW>480, WLOGP>5.6, MR>130, #atoms>70
Veber	No; 1 violation: TPSA>140
Egan	No; 2 violations: WLOGP>5.88, TPSA>131.6
Muegge	No; 4 violations: MW>600, XLOGP3>5, TPSA>150, H-don>5
Bioavailability Score	0.17

Medicinal Chemistry	
PAINS	0 alert
Brenk	2 alerts: imine_1, imine_2
Leadlikeness	No; 3 violations: MW>350, Rotors>7, XLOGP3>3.5

Figure S35. Physicochemical and pharmacokinetics properties of **5b** calculated using the free web tool SwissADME.

Molecule 4

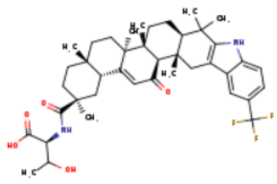

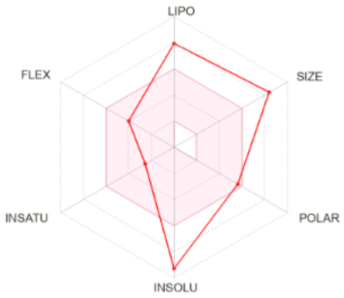

SMILES CC([C@@H](C(=O)O)NC(=O)[C@@]1(C)CC[C@]2([C@@H](C1)C1=CC(=O)[C@H]3[C@@]([C@@]1(CC2)C)(C)CC[C@@H]1[C@]3(C)Cc2c3cc(ccc3[nH]c2C1(C)C)C(F)(F)F)C)O

Physicochemical Properties	
Formula	C41H53F3N2O5
Molecular weight	710.87 g/mol
Num. heavy atoms	51
Num. arom. heavy atoms	9
Fraction Csp3	0.68
Num. rotatable bonds	6
Num. H-bond acceptors	8
Num. H-bond donors	4
Molar Refractivity	191.12
TPSA	119.49 Å²

Lipophilicity	
Log $P_{o/w}$ (iLOGP)	4.02
Log $P_{o/w}$ (XLOGP3)	8.40
Log $P_{o/w}$ (WLOGP)	9.28
Log $P_{o/w}$ (MLOGP)	5.11
Log $P_{o/w}$ (SILICOS-IT)	8.46
Consensus Log $P_{o/w}$	7.06

Water Solubility	
Log *S* (ESOL)	-9.27
Solubility	3.78e-07 mg/ml ; 5.32e-10 mol/l
Class	Poorly soluble
Log *S* (Ali)	-10.78
Solubility	1.19e-08 mg/ml ; 1.67e-11 mol/l
Class	Insoluble
Log *S* (SILICOS-IT)	-10.13
Solubility	5.28e-08 mg/ml ; 7.43e-11 mol/l
Class	Insoluble

Pharmacokinetics	
GI absorption	Low
BBB permeant	No
P-gp substrate	No
CYP1A2 inhibitor	No
CYP2C19 inhibitor	No
CYP2C9 inhibitor	No
CYP2D6 inhibitor	No
CYP3A4 inhibitor	Yes
Log K_p (skin permeation)	-4.67 cm/s

Druglikeness	
Lipinski	No; 2 violations: MW>500, MLOGP>4.15
Ghose	No; 4 violations: MW>480, WLOGP>5.6, MR>130, #atoms>70
Veber	Yes
Egan	No; 1 violation: WLOGP>5.88
Muegge	No; 2 violations: MW>600, XLOGP3>5
Bioavailability Score	0.56

Medicinal Chemistry	
PAINS	0 alert
Brenk	0 alert
Leadlikeness	No; 2 violations: MW>350, XLOGP3>3.5
Synthetic accessibility	6.77

Figure S36. Physicochemical and pharmacokinetics properties of **5c** calculated using the free web tool SwissADME.

Molecule 5

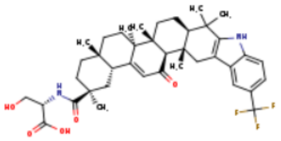

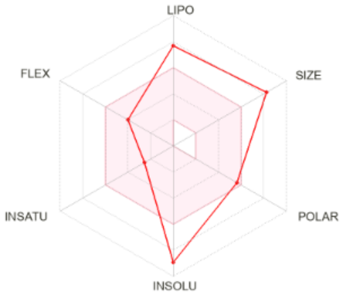

SMILES	OC[C@@H](C(=O)O)NC(=O)[C@@]1(C)CC[C@]2([C@@H](C1)C1=CC(=O)[C@H]3[C@@]([C@@]1(CC2)C)(C)CC[C@@H]1[C@]3(C)Cc2c3cc(ccc3[nH]c2C1(C)C)C(F)(F)F)C
**Physicochemical Properties**	
Formula	C40H51F3N2O5
Molecular weight	696.84 g/mol
Num. heavy atoms	50
Num. arom. heavy atoms	9
Fraction Csp3	0.68
Num. rotatable bonds	6
Num. H-bond acceptors	8
Num. H-bond donors	4
Molar Refractivity	186.31
TPSA	119.49 Å²
**Lipophilicity**	
Log $P_{o/w}$ (iLOGP)	4.60
Log $P_{o/w}$ (XLOGP3)	7.97
Log $P_{o/w}$ (WLOGP)	8.90
Log $P_{o/w}$ (MLOGP)	4.94
Log $P_{o/w}$ (SILICOS-IT)	8.21
Consensus Log $P_{o/w}$	6.92

**Water Solubility**	
Log *S* (ESOL)	-8.92
Solubility	8.40e-07 mg/ml ; 1.21e-09 mol/l
Class	Poorly soluble
Log *S* (Ali)	-10.33
Solubility	3.25e-08 mg/ml ; 4.67e-11 mol/l
Class	Insoluble
Log *S* (SILICOS-IT)	-10.12
Solubility	5.30e-08 mg/ml ; 7.60e-11 mol/l
Class	Insoluble
**Pharmacokinetics**	
GI absorption	Low
BBB permeant	No
P-gp substrate	No
CYP1A2 inhibitor	No
CYP2C19 inhibitor	No
CYP2C9 inhibitor	No
CYP2D6 inhibitor	No
CYP3A4 inhibitor	Yes
Log K_p (skin permeation)	-4.89 cm/s
**Druglikeness**	
Lipinski	No; 2 violations: MW>500, MLOGP>4.15
Ghose	No; 4 violations: MW>480, WLOGP>5.6, MR>130, #atoms>70
Veber	Yes
Egan	No; 1 violation: WLOGP>5.88
Muegge	No; 2 violations: MW>600, XLOGP3>5
Bioavailability Score	0.56
**Medicinal Chemistry**	
PAINS	0 alert
Brenk	0 alert
Leadlikeness	No; 2 violations: MW>350, XLOGP3>3.5
Synthetic accessibility	6.59

Figure S37. Physicochemical and pharmacokinetics properties of **5d** calculated using the free web tool SwissADME.

In Vivo Assay: Animals

Male CD-1 mice weighing between 30–40 g, were maintained at a temperature of 25 ± 2 °C, under a ups 12-hour light/dark cycle and 45–65% humidity throughout the experimental period. These animals were obtained from the UMADI (Animal Handling Unit for Teaching and Research) at the Metropolitan Autonomous University. The animals were given a standard rodent diet (Harlan Laboratories, Indianapolis, IN, USA) and had access to water *ad libitum*. The experimental protocol adhered to the International Guidelines for the Care and Use of Laboratory Animals (NOM-062-ZOO-1999, 2001 revision).

In Vivo Assay: Groups and Treatments

Animals were categorized into six groups, each consisting of four mice (n = 4). The experimental groups received an oral suspension of compounds **4**, **5a**, **5b,** and **5d**, prepared in a 10% Tween 20/saline solution, at a dosage of 50 mg/kg body weight. A control group was treated with only 10% Tween 20 saline solution. The final group was administered pioglitazone at a dose of 45 mg/kg, which is recognized as a reference drug for insulin sensitization.

In Vivo Assay: Oral Glucose Tolerance Test (OGTT)

An oral glucose tolerance test (OGTT) was performed to evaluate the anti-hyperglycemic effect. The experimental treatments were administered 30 min before oral glucose administration (2 g/kg): control Tween 20 at 10% (Karal S. A. de CV, León, Mexico), pioglitazone (45 mg/kg), and the experimental groups (**4** and its derivatives (**5b**, **5b** and **5d**, 50 mg/kg). Glycemia was measured with an Accu-Chek Performa glucometer (Roche, Mexico City, México) at 0, 30, 60, 90, and 120 min. The percentage variation in glycemia was calculated by comparing the selected post-administration glycemia (G_x) with the initial value (G_0) using the following formula:

$$\% \text{ Variation of Glycemia} = [(G_x - G_0) / G_0] \times 100,$$

where G_0 represents the initial glycemia values and Gx indicates the glycemia values at +30, +60, +90, and +120 minutes, respectively.

In Vivo Assay: Induction of experimental diabetes

After 10 hours of fasting, experimental diabetes mellitus mouse model was induced in mice through a single intraperitoneal injection of Streptozotocin (STZ, 100 mg/kg) followed by an intraperitoneal injection of Nicotinamide (20 mg/kg). Hyperglycemia was confirmed one week later by measuring blood glucose levels with a glucometer (Accu-Chek Performa; Roche). Mice exhibiting blood glucose concentrations greater than 200 mg/dL were selected for the next assays.

In Vivo Assay: Insulin Tolerance Test (ITT)

To evaluate the ability of compound **4** derivatives to enhance insulin sensitivity in STZ-induced diabetic CD-1 mice, compounds **4**, **5a**, **5b**, and **5d** were tested. GA and Pioglitazone served as positive controls. The compound **4** derivatives and the positive controls were administered to the diabetic animals, followed by the administration of human rapid-acting insulin (0.75 IU) after 30 minutes. Subsequently, glucose levels were measured every 15 minutes for up to 60 minutes. Glycemia was measured with an Accu-Chek Performa glucometer (Roche, Mexico City, México) at 0, 15, 30, 45, and 60 min. The percentage variation in glycemia was calculated by comparing the selected post-administration glycemia (G_x) with the initial value (G_0) using the following formula:

$$\% \text{ Variation of Glycemia} = [(G_x - G_0) / G_0] \times 100,$$

where G_0 represents the initial glycemia values and Gx indicates the glycemia values at +15, +30, +45, and +60 minutes, respectively.

***In Vitro* Pharmacologic Assays: Effect of GA, 4, 5a, 5b, and 5d Compounds on Cell Functionality.**

The cellular functionality test was performed using the MTT assay. GA, **4**, **5a**, **5b**, and **5d** compounds (0.2, 0.4, 0.8, and 1 µM), DMSO (1 %), and Pioglitazone (1, 5, 10, and 100 µM) as controls were evaluated to determine their non-toxic optimal concentration in C2C12 cells. **5b** and **5d** compounds reduced cellular functionality by 80 % at 1µM, while GA, **4**, and **5a** did not reduce cellular functionality at 1 µM (**Figure S35**). Therefore, in the following experiments, the concentration of 1 µM (GA, compound **4**, and its amide derivatives) and of 5 µM (Pioglitazone) were chosen.

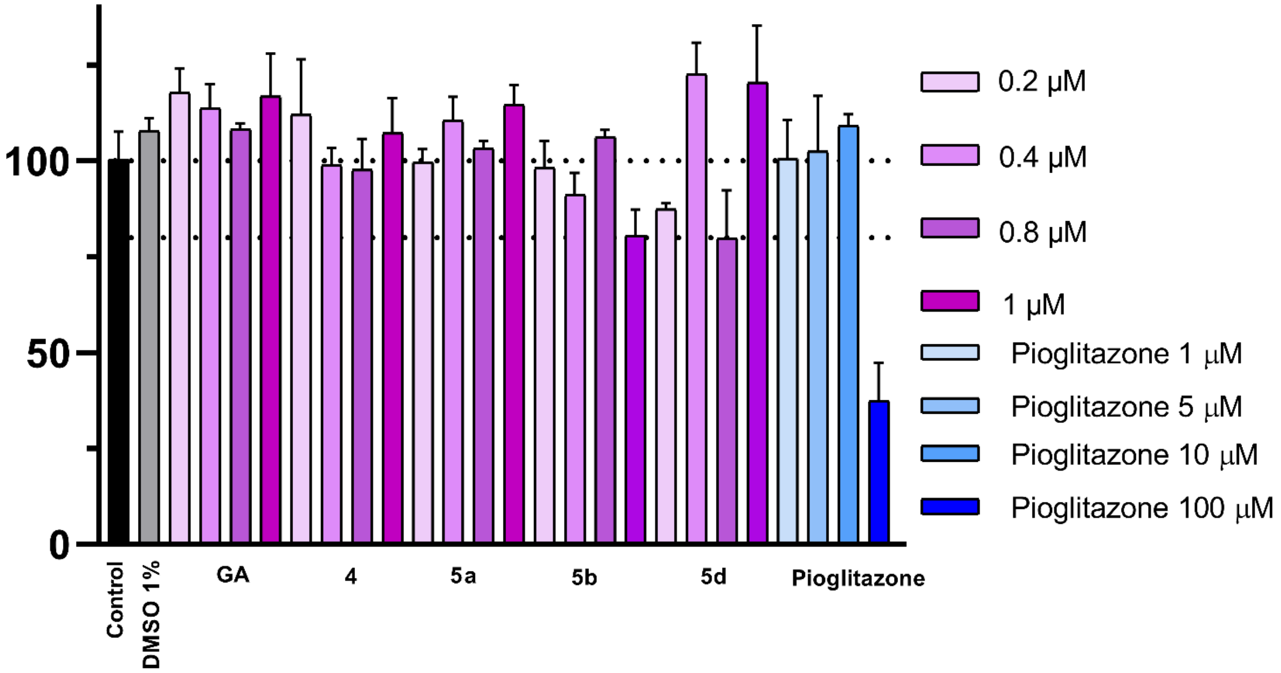

Figure 38. Effect of **GA**, **4**, and its amide derivatives on cell functionality of C2C12 myoblasts. Cells were treated with 0.2, 0.4, 0.8, and 1 µM for 24 h, and functionality was quantified by MTT assay. Mean ± SEM. (n = 4).

***In Vitro* Pharmacologic Assays:** mRNA Expression Analysis of GLUT4

C2C12 myoblasts were cultured under standard conditions and treated with compounds **GA**, **4**, **5a**, **5b**, or **5d**; pioglitazone (reference drug for PPARγ; 5 µM) and dissolved in DMSO 1%. Two experimental conditions were established: in the presence of insulin (0.8 µM) and in its absence, Following the treatments, RNA was isolated by the Trizol method (Invitrogen, Los Angeles, CA, USA).[16] Total RNA was reverse transcribed (RT) using the ImProm II kit (Promega, Madison, WI, USA).[17] One microgram of RNA was run using the electrophoresis method into a 2% agarose gel dyed with Eco-Stain colorant (BIOBASIC, Markham, ON, Canada). This gel was visualized on an imaging analyzer (BioRad, Redwood City, CA, USA) to confirm the RNA integrity. The complementary DNA (cDNA) was used to perform the PCR using SYBRGreen (Thermo Scientific, Waltham, MA, USA) with specific primers: 36B4 Forward 5′-AAGCGCGTCCTGGCATTGTCT-3′ Reverse 5′-CCGCAGGGGCAGCAGTGGT-3′ NM_007475.2; GLUT-4 Forward 5′-GATTCTGCTGCCCTTCTGTC-3′ Reverse 5′-ATTGGACGCTCTCTCTCCAA-3′ NM_009204.2. The expression level quantification was made as described by Giacoman-Martínez et al., 2017[18,19]

REFERENCES

(1) De-La-cruz-martínez, L.; Duran-Becerra, C.; González-Andrade, M.; Páez-Franco, J. C.; Germán-Acacio, J. M.; Espinosa-Chávez, J.; Torres-Valencia, J. M.; Pérez-Villanueva, J.; Palacios-Espinosa, J. F.; Soria-Arteche, O.; Cortés-Benítez, F. Indole-and Pyrazole-Glycyrrhetinic Acid Derivatives as Ptp1b Inhibitors: Synthesis, in Vitro and in Silico Studies. *Molecules* **2021**, *26* (14). https://doi.org/10.3390/molecules26144375.

(2) Álvarez-Almazán, S.; Solís-Domínguez, L. C.; Duperou-Luna, P.; Fuerte-Gómez, T.; González-Andrade, M.; Aranda-Barradas, M. E.; Palacios-Espinosa, J. F.; Pérez-Villanueva, J.; Matadamas-Martínez, F.; Miranda-Castro, S. P.; Mercado-Márquez, C.; Cortés-Benítez, F. Anti-Diabetic Activity of Glycyrrhetinic Acid Derivatives FC-114 and FC-122: Scale-Up, In Silico, In Vitro, and In Vivo Studies. *Int. J. Mol. Sci.* **2023**, *24* (16). https://doi.org/10.3390/ijms241612812.

(3) Mendoza-Jasso, M. E.; Pérez-Villanueva, J.; Alvarado-Rodríguez, J. G.; González-Andrade, M.; Cortés-Benítez, F. 3-Benzylaminomethyl Lithocholic Acid Derivatives Exhibited Potent and Selective Uncompetitive Inhibitory Activity Against Protein Tyrosine Phosphatase 1B (PTP1B). *ACS Omega* **2024**. https://doi.org/10.1021/acsomega.4c04948.

(4) Hanwell, M. D.; Curtis, D. E.; Lonie, D. C.; Vandermeersch, T.; Zurek, E.; Hutchison, G. R. Avogadro: An Advanced Semantic Chemical Editor, Visualization, and Analysis Platform. *J. Cheminform.* **2012**, *4* (1), 17. https://doi.org/10.1186/1758-2946-4-17.

(5) Campsteyn, H.; Dupont, L.; Lamotte, J.; Dideberg, O.; Vermeire, M. Crystal and Molecular Structure of Glycyrrhetinic Acid Acetone Monohydrate. *Acta Crystallogr. B* **1977**, *33* (11), 3443–3448. https://doi.org/10.1107/S0567740877011169.

(6) López-Sánchez, M.; Mendoza-Mota, H.; De-la-Cruz-Martínez, L.; Matadamas-Martínez, F.; Torres-Chacón, D. L.; Martínez-Arellano, R.; Palacios-Espinosa, J. F.; Pérez-Villanueva, J.; González-Andrade, M.; Páez-Franco, J. C.; Almanza-Pérez, J. C.; Cortés-Benítez, F. Optimization of Indole- and Pyrazole-Fused Glycyrrhetinic Acid Derivatives as Potent PTP1B Inhibitors: In Silico, In Vitro, In Vivo, and Metabolomic Studies. *ACS Bio & Med Chem Au* **2025**. https://doi.org/10.1021/acsbiomedchemau.5c00164.

(7) De-la-Cruz-Martínez, L.; Martínez-Arellano, R.; López-Sánchez, M.; Alvarado-Rodríguez, J. G.; Torres-Valencia, J. M.; Equihua-González, D.; Almanza-Pérez, J. C.; Pérez-Villanueva, J.; González-Andrade, M.; Páez-Franco, J. C.; Cortés-Benítez, F. Impact of C18 Epimerization of Indole- and Pyrazole-Fused 18β-Glycyrrhetinic Acid Derivatives on PTP1B and TCPTP Inhibitory Activity: Synthesis, In Vitro, and In Silico Studies. *ChemMedChem* **2025**. https://doi.org/10.1002/cmdc.202500350.

(8) Krieger, E.; Joo, K.; Lee, J.; Lee, J.; Raman, S.; Thompson, J.; Tyka, M.; Baker, D.; Karplus, K. Improving Physical Realism, Stereochemistry, and Side-chain Accuracy in Homology Modeling: Four Approaches That Performed Well in CASP8. *Proteins: Structure, Function, and Bioinformatics* **2009**, *77* (S9), 114–122. https://doi.org/10.1002/prot.22570.

(9) Krieger, E.; Vriend, G. YASARA View—Molecular Graphics for All Devices—from Smartphones to Workstations. *Bioinformatics* **2014**, *30* (20), 2981–2982. https://doi.org/10.1093/bioinformatics/btu426.

(10) Lindorff-Larsen, K.; Piana, S.; Palmo, K.; Maragakis, P.; Klepeis, J. L.; Dror, R. O.; Shaw, D. E. Improved Side-chain Torsion Potentials for the Amber Ff99SB Protein Force Field. *Proteins: Structure, Function, and Bioinformatics* **2010**, *78* (8), 1950–1958. https://doi.org/10.1002/prot.22711.

(11) Wang, J.; Wolf, R. M.; Caldwell, J. W.; Kollman, P. A.; Case, D. A. Development and Testing of a General Amber Force Field. *J. Comput. Chem.* **2004**, *25* (9), 1157–1174. https://doi.org/10.1002/jcc.20035.

(12) Jakalian, A.; Jack, D. B.; Bayly, C. I. Fast, Efficient Generation of High-quality Atomic Charges. AM1-BCC Model: II. Parameterization and Validation. *J. Comput. Chem.* **2002**, *23* (16), 1623–1641. https://doi.org/10.1002/jcc.10128.

(13) Mark, P.; Nilsson, L. Structure and Dynamics of the TIP3P, SPC, and SPC/E Water Models at 298 K. *J. Phys. Chem. A* **2001**, *105* (43), 9954–9960. https://doi.org/10.1021/jp003020w.

(14) Essmann, U.; Perera, L.; Berkowitz, M. L.; Darden, T.; Lee, H.; Pedersen, L. G. A Smooth Particle Mesh Ewald Method. *J. Chem. Phys.* **1995**, *103* (19), 8577–8593. https://doi.org/10.1063/1.470117.

(15) Krieger, E.; Vriend, G. New Ways to Boost Molecular Dynamics Simulations. *J. Comput. Chem.* **2015**, *36* (13), 996–1007. https://doi.org/10.1002/jcc.23899.

(16) Chomczynski, P. *A Reagent for the Single-Step Simultaneous Isolation of RNA, DNA and Proteins from Cell and Tissue Samples*. https://www.researchgate.net/publication/15506061.

(17) Sharma, P.; Hajam, Y. A.; Kumar, R.; Rai, S. Complementary and Alternative Medicine for the Treatment of Diabetes and Associated Complications: A Review on Therapeutic Role of Polyphenols. *Phytomedicine Plus*. Elsevier B.V. February 1, 2022. https://doi.org/10.1016/j.phyplu.2021.100188.

(18) Giacoman-Martínez, A.; Alarcón-Aguilar, F. J.; Zamilpa, A.; Hidalgo-Figueroa, S.; Navarrete-Vázquez, G.; García-Macedo, R.; Román-Ramos, R.; Almanza-Pérez, J. C. Triterpenoids from Hibiscus Sabdariffa L. with PPAR δ / γ Dual Agonist Action: In Vivo, in Vitro and in Silico Studies. *Planta Med.* **2019**, *85* (5), 412–423. https://doi.org/10.1055/a-0824-1316.

(19) Ortiz-Barragán, E.; Estrada-Soto, S.; Giacoman-Martínez, A.; Alarcón-Aguilar, F. J.; Fortis-Barrera, Á.; Marquina-Rodríguez, H.; Gaona-Tovar, E.; Lazzarini-Lechuga, R.; Suárez-Alonso, A.; Almanza-Pérez, J. C. Antihyperglycemic and Hypolipidemic Activities of Flavonoids Isolated from Smilax Dominguensis Mediated by Peroxisome Proliferator-Activated Receptors. *Pharmaceuticals* **2024**, *17* (11). https://doi.org/10.3390/ph17111451.
